# Supplementary material for: Childhood Acute Illness and Nutrition (CHAIN) Network: a protocol for a multi-site prospective cohort study to identify modifiable risk factors for mortality among acutely ill children in Africa and Asia
Source: BMJ Open. 2019 May 5;9(5):e028454. doi: 10.1136/bmjopen-2018-028454 (PMC6502050; doi:10.1136/bmjopen-2018-028454)
Supplement: Supplementary data [file bmjopen-2018-028454supp001.pdf]

# CHAIN Enrolment CRF v1.63
# CHAIN Number [1][0] [0][0][3] [ ][ ][ ]

| Eligibility Checklist | | |
|---|---|---|
| **Age between 2 months and before 2nd birthday** | Y | N - ineligible |
| **Being admitted to hospital because of acute illness** | Y | N- ineligible |
| **Parent or guardian able and available to consent** | Y | N- ineligible |
| **Able to feed orally in usual state of health** | Y | N- ineligible |
| **Known congenital syndrome** | Y- ineligible | N |
| **Cleft palate** | Y- ineligible | N |
| **Known congenital cardiac disease** | Y- ineligible | N |
| **Known terminal illness e.g. cancer** | Y- ineligible | N |
| **Admission for surgery, or likely to require surgery within 6m** | Y- ineligible | N |
| **Admission for trauma?** | Y- ineligible | N |
| **Sibling enrolled in study** | Y- ineligible | N |
| **Previously enrolled** | Y- ineligible | N |

## Part 1

| Admission to Hospital and Study Enrolment | | | | | |
|---|---|---|---|---|---|
| **DATE arrived at the hospital** | __ __/__ __/__ __ __ __ *D D / M M / Y Y Y Y* | **TIME arrived at the hospital** | __ __: __ __ *24h Clock* | ☐ Arrival time unknown | |
| **DATE of enrolment** i.e. date consented and seen by research team | __ __/__ __/__ __ __ __ *D D / M M / Y Y Y Y* | **TIME of enrolment** | __ __: __ __ *24h Clock* | **Sex** | ☐ Male ☐ Female |
| **DOB** | __ __/__ __/__ __ __ __ *D D / M M / Y Y Y Y* | **Is the DOB:** | ☐ True ☐ Estimated* | **Child's Initials** | ___ ___ ___ |
| **Brought into hospital by:** *Select all that apply* | ☐ Mother | ☐ Father | ☐ Grandparent | ☐ Aunt/Uncle | |
| | ☐ Sibling <18 | ☐ Sibling >18 | ☐ Carer (care home) | ☐ Other__________ | |

**if DOB is estimated, and the day is uncertain, write '15' for DD*

## Presenting Complaints

☐ Fever / Hotness of body
☐ Difficulty breathing
☐ Cough<14 days
☐ Cough>14days
☐ Poor feeding/ Weight loss
☐ Rash/ skin lesion
☐ Vomiting
☐ Diarrhoea <14 days
☐ Diarrhoea >14 days
☐ Blood in stool
☐ Developmental delay
☐ Other *(only one complaint, if not covered by above options)* ______________________________________________
☐ Lethargy
☐ Convulsions
☐ Altered consciousness
☐ Not feeding
☐ Body swelling / limb swelling/ Oedema

# CHAIN Enrolment CRF v1.63
# CHAIN Number [1][0] [0][0][3] [ ][ ][ ]

| Initial Observations (to be taken at time of examination by research team) | | | |
|---|---|---|---|
| **Axillary temperature** | ____ ____. ____ °C | **Respiratory rate** *Count for 1 minute* | ____ ____ ____/minute |
| **Heart rate** *Count for 1 minute* | ____ ____ ____/minute | | |
| **SaO2** *To be taken from finger or toe using pulse oximeter* | ____ ____ ____ % *Leave blank if unrecordable* | ☐ Measured in Oxygen ☐ Measured in Room Air | ☐ Unrecordable |

| Anthropometry | | | | | |
|---|---|---|---|---|---|
| **Weight** *to be taken using SECA scales for CHAIN study* | ___ ___ . ___ ___kg | | **Length** *to be taken using SECA 416 infantometer provided for CHAIN study* | Measurer 1 | ____ ____. ____ cm |
| | | | | Measurer 2 | ____ ____ . ____ cm |
| **MUAC** *To be taken using MUAC tape for CHAIN study* | Measurer 1 | ____ ____ . ____ cm | **Head circumference** *To be taken using CHAIN measuring tape* | Measurer 1 | ____ ____. _____cm |
| | Measurer 2 | ____ ____ . ____ cm | | Measurer 2 | ____ ____. _____cm |
| **Oedema** | ☐ None ☐ + ☐ ++ ☐ +++ | | **Initials** | Measurer 1 __ __ __ | Measurer 2 __ __ __ |

*NB: If the child is unwell the Length and Head Circumference can be taken at a later time.*

| Current Health | |
|---|---|
| **Previously admitted to hospital.** *Include other hospitals / health centres. Select 1* | ☐ No ☐ < 1 week ago ☐ 1 weeks-1month ago ☐ >1month ago |
| **Any medication last 7 days.** *Select all that apply* | ☐ No medication ☐ Antibiotic ☐ Antimalarial ☐Traditional ☐ Deworming ☐ Vitamin ☐ Paracetamol or Ibuprofen ☐ Yes, but unknown ☐Other |
| **Urine volume in last 24hrs?** *Select 1* | ☐ Not passing urine ☐ Less than normal ☐ Normal or greater ☐ Unknown |

## Examination

*Examination should be performed by CHAIN study clinician trained in clinical examination of children, and able to formulate a diagnosis based on clinical history and findings. Refer to Clinical Examination SOP*

| | | | | |
|---|---|---|---|---|
| **Airway** *(select one)* | ☐ **Clear** | ☐ Needs active support | ☐ Obstructed/Stridor | |
| **Breathing** *(select all that apply)* | ☐ **Normal – no concerns**, (move to circulation) | | | |
| | ☐ Central cyanosis | ☐ Nasal flaring | ☐ Reduced air-entry | |
| | ☐ Wheeze | ☐ Acidotic Breathing | ☐ Grunting | |
| | ☐ Lower chest wall indrawing | ☐ Crackles | ☐ Dull to percussion<br>☐ Head nodding | |
| **Circulation:** | | | | |
| **Cap Refill** (select one) | ☐ >3s | ☐ 2-3s | ☐ <2s | |
| **Cold Peripheries**(select one) | ☐ Shoulder | ☐ Elbow | ☐ Hand | ☐ Warm peripheries |
| **Disability:** | | | | |
| **Conscious level**(select one) | ☐ **Alert** | ☐ Voice | ☐ Pain | ☐ Unresponsive |
| **Fontanelle**(select one) | ☐ **Normal** | ☐ Bulging | ☐ Sunken | ☐ Not present |
| **Tone**(select one) | ☐ **Normal** | ☐ Hypertonic | | ☐ Hypotonic |
| **Posture**(select one) | ☐ **Normal** | ☐ Decorticate | | ☐ Decerebrate |
| **Activity**(select one) | ☐ **Normal** | ☐ Irritable/Agitated | | ☐ Lethargic |
| **Dehydration**: | | | | |
| **Sunken eyes?** | ☐ Y | ☐ N | | |
| **Skin pinch** *(select one)* | ☐ >2 seconds | ☐ <2 seconds | ☐ Immediate | |
| **Drinking/Breastfeeding** *(Select one)* | ☐ **Normal** | ☐ Poorly | ☐ Not drinking | ☐ Eager / Thirsty |
| **Abdomen** *(select any that apply)* | ☐ **Normal – no concerns** | ☐ Distension | ☐ Hepatomegaly | |
| | ☐ Tenderness | ☐ Splenomegaly | ☐ Other abdominal mass | |
| **Signs of Rickets** | ☐ **None** | ☐ Wrist widening<br>☐ Rachitic rosary | ☐ Swollen knees<br>☐ Bow legs | ☐ Frontal bossing |
| **Jaundice** *(Select one)* | ☐ **Not jaundiced** | ☐ + | ☐ ++ | ☐ +++ |
| **ENT/Oral/Eyes** *(select any that apply)* | ☐ Mouth Normal | ☐ Ears Normal | ☐ Eyes Normal | |
| | ☐ Oral ulceration | ☐ Pus from ear | ☐ Conjunctivitis | |
| | ☐ Oral candidiasis | ☐ Tender swelling behind ear (mastoiditis) | ☐ Eye discharge | |
| | ☐ Stomatitis | ☐ Lymphadenopathy | ☐ Visual impairment | |
| **Skin** *(select any that apply)* | ☐ Normal | ☐ Hyperpigmentation | ☐ Depigmentation | |
| | ☐ Broken skin | ☐ Dermatitis | ☐ 'Flaky paint' | |
| | ☐ Cellulitis | ☐ Impetigo | ☐ Pustules | |
| | ☐ Vesicles | ☐ Desquamation | ☐ Macular or papular | |
| **Site of skin lesions.** *(select any that apply)* | ☐ Not applicable (No rash) | ☐ Trunk | ☐ Face / scalp | ☐ Legs |
| | ☐ Palms / soles | ☐ Buttocks | ☐ Arms | ☐ Perineum |

# CHAIN Enrolment CRF v1.63
# CHAIN Number [1][0] [0][0][3] [ ][ ][ ]

| Suspected Chronic Conditions | | | |
|---|---|---|---|
| *Select confirmed, suspected or none for all conditions:* | Confirmed *(diagnosed previously/ recorded)* | Suspected *(clinician's impression)* | None |
| **Cerebral palsy/neurological problem/ epilepsy** | ☐ | ☐ | ☐ |
| **Sickle Cell disease** *family history, crisis* | ☐ | ☐ | ☐ |
| **Thalassaemia** | ☐ | ☐ | ☐ |
| **Visual problem / Blindness** *Not fixing and following* | ☐ | ☐ | ☐ |
| **Losing weight or not gaining weight** | ☐ | ☐ | ☐ |

| TB Screening | | | |
|---|---|---|---|
| Known TB (on treatment) | Child has cough >14 days | Household contact has TB, or cough >14 days | Child has suspected extra-pulmonary TB |
| Y N | Y N | Y N | Y N |

| Feeding | | | | |
|---|---|---|---|---|
| **Currently in outpatient nutrition program?** *Select one.* | ☐ Supplementary *(corn soy blend, RUSF, khichuri, halwa)* | ☐ Therapeutic *(RUTF, Plumpy-nut)* | ☐ None | |
| **Has the child eaten these nutrition products in the last 3 days?** | ☐ Supplementary | ☐ Therapeutic | ☐ None | |
| **Currently Breastfeeding?** | ☐ Y ☐ N | **If yes is the child taking anything else (exclude medicine)?** | ☐ Y ☐ N ☐ N/A | |
| **If NO breastfeeding at all, age stopped in months?** *(select one)* | ☐ 0-3m ☐ 4-6m ☐ 7-12m ☐ >12m ☐ Unknown ☐ N/A | | | |
| **What did the child receive other than breast milk in the first 3 days of life?** *Select all that apply* *Do not include medications e.g. ARV.* | ☐ Sweetened/sugar water | ☐ Formula/powder milk | ☐ Animal milk | |
| | ☐ Fruit Juice | ☐ Tea | ☐ Other | |
| | ☐ Water | ☐ Porridge/pulp | ☐ Gutthi / gripe water | |
| | ☐ Pure Honey | ☐ Glycerine | ☐ Nothing | |

| **Vaccinations** – *Ask carer or check book / card if available* | | | | | | | |
|---|---|---|---|---|---|---|---|
| **BCG scar** | ☐ Yes ☐ No | **Rotavirus** | ☐ Book | ☐ Self report | ☐ Not received | **Doses received:** | 3 2 1 ☐ Unknown |
| **Measles** | ☐ Book ☐ Self report | **Pneumococcus** | ☐ Book | ☐ Self report | ☐ Not received | **Doses received:** | 3 2 1 ☐ Unknown |
| | ☐ Not received ☐ Unknown | **DTP/Penta** | ☐ Book | ☐ Self report | ☐ Not received | **Doses received:** | 3 2 1 ☐ Unknown |
| | | **Polio** | ☐ Book | ☐ Self report | ☐ Not received | ☐ Unknown | |

| CLINICIANS IMPRESSION OF RISK | | | | | | |
|---|---|---|---|---|---|---|
| *How likely does the clinical team think this child is to die during this admission? Select one* | | | | | | |
| ☐ Almost certainly not | ☐ Very unlikely | ☐ Quite unlikely | ☐ Unsure | ☐ Quite likely | ☐ Very likely | ☐ Almost certainly |

| Immediate Clinical Investigations and HIV status | | | | | | |
|---|---|---|---|---|---|---|
| **Malaria RDT** *circle result* | Positive | | Negative | | Not done | |
| **Blood glucose** | ___ ___ . ___ mmol/L | | **Time glucose measured** | ___ ___:___ ___ *24h clock* ☐ Unknown | | |
| **Urine Dipstick** *(can be done at any time during admission)* **Urine sample stored?** Y N | Protein | Nitrites | Leucocytes | Blood | Ketones | Glucose |
| ☐ Not done ☐ Bag ☐ Clean catch | None + ++ +++ | Pos Neg | None + ++ +++ | None + ++ +++ | None + ++ +++ | None + ++ +++ |

| | | | | |
|---|---|---|---|---|
| **HIV status known?** | ☐ Yes, known PCR positive | ☐ Yes, antibody positive, unknown PCR status | ☐ Yes, known exposed, known PCR negative (children under 18m with PCR result SEEN BY RESEARCH TEAM. If not seen select below and perform HIV RDT | |
| | ☐ No, known to be HIV exposed, but child untested | | ☐ No, child not tested, not known to be exposed | |

| | | | | |
|---|---|---|---|---|
| **If child known HIV positive or exposed** | **On any ART?** | ☐Y ☐N ☐ Unknown | **If on treatment,** ARV 1____________ ARV 2____________ ARV 3____________ | **If on prophylaxis** ☐Nevirapine prophylaxis only ☐AZT + NVP prophylaxis ☐Caregiver unsure |
| | **Co-trimoxazole** *select one* | ☐ On prophylactic dose co-trimoxazole ☐On high dose co-trimoxazole | ☐ Not on co-trimoxazole | ☐Caregiver unsure |
| **If not known positive** | **HIV RDT now** *select one* | ☐Reactive / positive PCR sent: ☐Y ☐ N | ☐Non-Reactive / Negative | ☐Declined |

| | | | | | | |
|---|---|---|---|---|---|---|
| **HIV test offered to caregiver?** | ☐Yes, Reactive | ☐Yes, Non-reactive | ☐Yes, but Declined | ☐No, Caregiver is known positive | ☐Missed | ☐ N/A child in care home |
| **Did the mother have interventions or medication during delivery to prevent transmission of HIV to baby?** | ☐Yes | | ☐No | | ☐Unknown | |

CHAIN Number [1][0] [0][0][3] [ ][ ][ ]

| INITIAL TREATMENT | | | |
|---|---|---|---|
| **Admitted to:** *select one* | ☐ Admission to ward | ☐ Admission to HDU | ☐ Admission to ICU |
| **Date and time First antibiotics given** | __ __/ __ __/ __ __ __ __ | ___ ___:___ ___ *24h clock* | ☐Not given |
| **Intravenous Antibiotics Given?**<br><br>☐ **Not given** | ☐ Benzylpenicillin | ☐ Gentamicin | ☐ Ceftriaxone / Cefotaxime |
| | ☐ Co-amoxiclav/ Augmentin | ☐ Flu/Cloxacillin | ☐ Chloramphenicol |
| | ☐ Ampicillin | ☐ Amikacin | ☐ Meropenem / Imipenem |
| | ☐ Levofloxacin | ☐ Vancomycin | ☐ Metronidazole |
| | ☐ Ceftazidime | ☐ Pivmecillinam | |
| | ☐ Other___________________________________________ | | |
| **Oral Antibiotics Given?**<br><br>☐ **Not given** | ☐ Amoxicillin | ☐ Erythromycin | ☐ Azithromycin |
| | ☐ Co-trimoxazole | ☐ Metronidazole | ☐ Ciprofloxacin |
| | ☐ Cefalexin / cefaclor | ☐ Co-amoxiclav / Augmentin | ☐ Nalidixic acid |
| | ☐ Penicillin | ☐ Flucloxacillin | ☐ Levofloxacin |
| | ☐ Other _____________ | | |
| **Initial treatment <u>given</u>**<br>*First 6 hours.*<br>*Select any that apply.*<br>*For IV fluid bolus, and IV fluids specify type and volume in ml, and duration* | ☐ IV Fluid Bolus | ☐ IV Maintenance Fluids | |
| | ☐ Oxygen | ☐ CPAP | |
| | ☐ IV Glucose ☐ Oral Glucose | ☐ Warmth (heater, warmed fluids) | |
| | ☐ Blood transfusion | ☐ Commercial F75 | |
| | ☐ Phenobarbitone | ☐ Commercial F100 | |
| | ☐ Diazepam | ☐ Locally prepared F75/ milk suji | |
| | ☐ Paracetamol | ☐ Local prepared F100 / milk suji 100 | |
| | ☐ Ibuprofen | ☐ Expressed breast milk | |
| | ☐ Diclofenac | ☐ Dilute F100/ dilute milk or formula | |
| | ☐ Salbutamol / atrovent / other bronchodilator | ☐ Other milk/ formula/ feed<br>☐ RUTF | |
| | ☐ Prednisolone/ dexamethasone/ hydrocortisone | ☐ Nasogastric tube | |
| | ☐ Adrenaline | ☐ Multivitamin | |
| | ☐ Zinc | ☐ Micronutrients | |
| | ☐ Folic acid | ☐ Vitamin A | |
| | ☐ Antimalarial (any) | ☐ Albendazole / deworming | |
| | ☐ ReSoMal | ☐ Other<br>______________________________ | |
| | ☐ ORS | | |

**Suspected Initial Diagnoses:**
*Clinical diagnosis should be based on examination and investigation findings.*
*Tick the three most likely diagnoses.*

| **Respiratory** | **Infection** | **CNS** |
|---|---|---|
| ☐ LRTI/pneumonia | ☐ Gastroenteritis | ☐ Febrile convulsions |
| ☐ Bronchiolitis | ☐ Sepsis | ☐ Epilepsy |
| ☐ URTI | ☐ Malaria | ☐ Probable meningitis |
| ☐ Pulmonary TB | ☐ Extra pulmonary TB | ☐ Other encephalopathy |
| ☐ Otitis media | ☐ Soft tissue infection | ☐ Hydrocephalus |
| ☐ Asthma | ☐ UTI | ☐ Developmental delay |
| **General** | ☐ HIV related illness | ☐ Cerebral palsy |
| ☐ Anaemia | ☐ Measles | **Other suspected diagnosis:** |
| ☐ Sickle Cell Disease | ☐ Varicella | ☐ Other |
| ☐ Thalassaemia | ☐ Osteomyelitis | ☐ Unknown |
| ☐ Renal impairment | ☐ Febrile illness unspecified | ☐ Failed appetite test only |
| ☐ Nephrotic syndrome | ☐ Enteric fever | |
| ☐ Nephritis | | |
| ☐ Liver dysfunction | | |
| ☐ Ileus | | |
| ☐ Congenital cardiac disease | | |

# CHAIN Enrolment CRF v1.63
# CHAIN Number [1][0] [0][0][3] [ ][ ][ ]

| Admission Core Cohort Investigations and Sample Collection | | | | | |
|---|---|---|---|---|---|
| **CBC taken** | ☐ Y | ☐ N | **Plain Blood (serum)** | ☐ Y | ☐ N |
| **Clinical chemistry taken** | ☐ Y | ☐ N | **Blood spot taken** | ☐ Y | ☐ N |
| **EDTA 2ml blood taken** | ☐ Y | ☐ N | **Blood culture taken** *(if available at site)* | ☐ Y BEFORE ABX<br>☐ Y AFTER ABX | ☐ N |
| **EDTA 0.5ml blood taken** | ☐ Y | ☐ N | **Blood gas taken** *(if available at site)* | ☐ Capillary<br>☐ Venous | ☐ N |
| **Unable to take blood samples, why?** | ☐ Difficult venepuncture | ☐ Child uncooperative | ☐ Parent refused | ☐ Other | |

| | | | | |
|---|---|---|---|---|
| **Rectal swabs taken** | ☐ Y BEFORE ABX<br>☐ Y AFTER ABX | ☐ N | Number taken ☐1 ☐2 | Time taken___ ___: ___ ___ |
| **Stool sample** | Taken in first 24h? | ☐ Y | ☐ N | Time taken___ ___: ___ ___ |

| | | | | |
|---|---|---|---|---|
| **Chest x-ray indicated** *(respiratory signs symptoms)* | ☐ Yes, but too unwell | ☐ Yes, done | ☐ Indicated but not done, unclear | ☐ Not indicated |
| **Lumbar puncture indicated** *(signs of meningitis documented)* | ☐ Yes, but too unwell | ☐ Yes, done | | ☐ Not indicated |

| | |
|---|---|
| **Blood Samples taken by (initials)** | __ __ __ |
| **Rectal Swabs taken by (initials)** | __ __ __ |

| | | | |
|---|---|---|---|
| **CRF Completed by (Initials) – to be signed when complete.** *Do not sign if any fields are empty* | __ __ __ | Date<br>__ __/__ __/__ __ __ __<br>*D D / M M / Y Y Y Y* | Time<br>__ __: __ __ |

## PART 2

| CHAIN ADMISSION CRF: SOCIAL INFORMATION. |
|---|
| *To be completed within 48h of admission when child is stable. This should ideally be done in a conversational and unhurried way, with the interviewer sitting with the caregiver.* |

| Initials of person interviewing caregiver and completing part 2<br><br>___ ___ ___<br><br>☐ Doctor ☐ Clinical officer ☐ Nurse ☐ Field worker ☐ Research Assistant ☐ Other | Date<br><br>__ __/ __ __/ __ __ __ __<br>*D D/ M M/ Y Y Y Y*<br><br>Time<br><br>___ ___: ___ ___ |
|---|---|

| **Who is being interviewed?** | | | | | |
|---|---|---|---|---|---|
| ☐ Primary caregiver only | ☐ Care home staff | ☐ Primary caregiver and one other person | ☐ Primary caregiver and more than one other person | ☐ One person who is not the primary caregiver | ☐ More than one person who is not the primary caregiver |

| **Care-seeking Behaviour** | | | |
|---|---|---|---|
| **Was the child in generally good health before this illness?** | ☐ Y | ☐ N | ☐ Unknown |
| **If No, how long has the child had this problem of generally bad health?** | __ __ weeks | ☐ N/A | |
| **Does the child have health insurance?** | ☐ Y | ☐ N | ☐ Unknown |

**What was the main reason for bringing the child to this hospital today?** *Reasons given, select one*

| | | |
|---|---|---|
| ☐ Referred by health care worker | ☐ Caregiver concern of child's condition | ☐ Received money for transport to hospital (e.g. from family, neighbour, paid work)? |
| ☐ Relative / neighbour concern of child's condition | ☐ Primary caregiver returned home e.g. if working away | ☐ Other |

**How did you travel to the hospital?** *Select all that apply*

☐Car/ Taxi ☐ Ambulance ☐ Bus ☐Motorbike ☐ Tuk-tuk /CNG ☐ Cycle rickshaw ☐ Train ☐ Walking ☐ Other

| **How long did it take you to travel to hospital?** | ☐ <1h | ☐ 1- < 2h | ☐ 2-4h | ☐ >4h | ☐ > 1 day |
|---|---|---|---|---|---|

| **How much did it cost the family to travel to hospital today (in local currency?** *Estimate amount. If walked or free ambulance write 0* | ___ ___ ___ ___ |
|---|---|

**Have you sought treatment for this illness prior to coming to hospital?** *Select all that apply*

☐ No treatment sought ☐ Shop ☐ Government hospital ☐ Government dispensary ☐ Traditional Healer

☐ Pharmacy ☐ Private Medical Facility/ NGO ☐ Herbalist ☐ Homeopathist ☐ Other

| **Received treatment from traditional healer, homeopathist or herbalist in last 4 weeks?** | Y | N |
|---|---|---|

**Child's Health Status Before Admission**

**Before this illness, how did this child's health compare to other children of similar age in your neighbourhood?** *Select one*

☐Similar ☐Better ☐Worse ☐Don't know

**Before this illness, how did this child's health compare to his/her siblings at a similar age?** *Select one*

☐Similar ☐Better ☐Worse ☐ Don't know ☐ N/A only child

| Birth History | |
|---|---|
| **Source of information** | ☐ Maternal/caregiver recall ☐ Book/medical records |
| **Birth weight** | ___ .___ ___kg ☐Unknown |
| **Birth details**<br>*Select any that apply* | ☐ Premature ☐ Born small <2.5kg ☐Twin/multiple birth ☐ Born at term ☐ Unknown |
| **Delivery location**<br>*Select one* | ☐ Born in hospital ☐ Community facility/clinic with midwife/nurse midwife/doctor<br>☐ Home without birth attendant ☐ Home with traditional birth attendant (untrained) ☐ Home with midwife/nurse<br>☐ Other ☐ Unknown |
| **Delivery details**<br>*Select all that apply* | ☐ Normal, spontaneous vaginal delivery ☐ Assisted delivery (forceps, ventouse) ☐ Caesarean section<br>☐ Admitted neonatal unit ☐ Mother admitted to hospital >48h ☐ Unknown |
| **Mother's age at first pregnancy** | ___ ___years ☐ unknown |
| **Mother's age now** | ___ __years ☐ unknown |
| **Participant birth order** | ___ ___ of ___ ___ total live births<br>(*e.g. if youngest of 3 children 3 of 3, if oldest of 3 children 1 of 3)* |
| **Are the biological parents of this child consanguineous?**<br>*Ask if parents have relatives in common or are related.* | ☐ Yes ☐ No ☐ Unknown |

# CHAIN Enrolment CRF v1.63
# CHAIN Number [1][0] [0][0][3] [ ][ ][ ]

| **Primary Caregiver Information**<br>***This is the person who has responsibility for day to day care of the child, but is not a substitute carer such as childminder or grandparent who cares for child whilst, for example, mother is at work.*** | | | |
|---|---|---|---|
| **Who is the Primary Caregiver?** *Select one* | ☐Biological Parent ☐Grandparent ☐Sibling ☐Aunt / Uncle / Cousin<br>☐ Stepmother / father ☐ Care home /orphanage ☐Other/ Unclear | | |
| **Is the child's biological father alive?** | ☐ Y ☐ N ☐ Unknown | **Is the child's biological mother alive?** | ☐ Y ☐ N ☐ Unknown |
| **Primary Care Giver Age** *Select one* | ☐ <18years ☐ >=18 years ☐ >50years ☐ N/A (care home or unclear) | | |
| **Primary Care Giver Sex** *Select one* | ☐ Male ☐ Female ☐ N/A | **Primary caregiver present at admission?** | ☐ Y ☐ N |
| **Has the primary caregiver lived in the same household as the child for the last 2 months?** | | | ☐ Y ☐ N<br>☐ N/A (care home) |
| **Marital status of primary caregiver** *Select one* | ☐ Married/ monogamous ☐ Married polygamous ☐ Single ☐ Separated / divorced ☐ Widowed ☐ N/A | | |
| **If not present at admission, where is the primary caregiver?** *Select one* | | | |
| ☐ Home ☐ Work ☐ School ☐ Unknown ☐ Other_______ ☐ N/A | | | |
| **If the primary caregiver is present, caregiver anthropometry:**<br>*Use locally available adult scales and stadiometer, and adult MUAC tapes provided by CHAIN.* | | | |
| ☐ **Primary caregiver not present during admission, or care home** | | | |
| **Weight** ___ ___. ___ kg | **MUAC** ___ ___ .____.cm | **Height:** ___ ___ ___cm | |
| **Education:** *Select highest level of education achieved* | ☐ None ☐ Primary ☐ Secondary ☐ Above secondary ☐ Unknown ☐ N/A care home | | |
| **Able to read?** | ☐ Y ☐ N ☐Unknown | **Is the primary caregiver primarily responsible for financial support and providing for the child?** | ☐ Y ☐ N |
| **Primary caregiver HIV status in last 6 months** *Select one* | ☐ Tested Positive ☐ Tested Negative ☐ Not tested or unknown | | |

**Have there been ANY changes to the child's social situation in the last 2 MONTHS?** *Select any that apply,*

| | | | |
|---|---|---|---|
| **Child moved to a different household** | **Y N** | **Relocation from rural to urban setting** *Select 'yes' even if this is temporary* | **Y N** |
| | | **Relocation from urban to rural setting** *Select 'yes' even if this is temporary* | **Y N** |
| | | **Relocation to live with different caregiver** *Select 'yes' even if this is temporary* | **Y N** |
| **Mother sick** | **Y N** | **Mother Died** | **Y N** |
| **Father sick** | **Y N** | **Father Died** | **Y N** |
| **Other primary caregiver sick** | **Y N N/A** | **Other primary caregiver died** | **Y N N/A** |
| **Primary caregiver changed** | **Y N** | **Child went into care home** | **Y N** |
| **Primary caregiver started employment / returned to school** | **Y N** | **Person providing for the child has lost income** | **Y N** |
| **Primary caregiver divorced / separated from partner** | **Y N** | **Primary caregiver in new relationship** | **Y N** |
| **Mother is pregnant** | **Y N** | **Mother gave birth** | **Y N** |
| **Other primary caregiver pregnant?** | **Y N N/A** | **Other primary caregiver gave birth** | **Y N N/A** |

**If primary caregiver has changed in the last 2 months, who was the child's previous primary caregiver?** *Select one*

☐Biologic Mother ☐Biologic Father ☐Sibling ≥18 years old ☐Sibling <18 years old

☐Grandparent ☐Aunt/Uncle/Cousin ☐Other ☐ N/A

| **Primary caregiver earns an income now?** *Ask the person accompanying the child and select one* | |
|---|---|
| ☐ Employed full time by someone else | ☐ Employed part time by someone else |
| ☐ Works for self | ☐ No work income |
| ☐ Works casually/irregularly for someone | ☐ Don't know |
| If works casually, Occupation: | ☐ N/A care home |

| **How many days worked a week?** *Select one* | ☐ **<3** | ☐ **3-5** | ☐ **>5** | ☐ N/A, does not work for income |
|---|---|---|---|---|

| **If the primary caregiver earns, main source of income?** *Select one* | | | |
|---|---|---|---|
| ☐ Farmer | ☐ Business/trader | ☐ Labourer | ☐ Domestic work |
| ☐ Other private sector employment | ☐ Public sector employment | ☐ Retired with pension income | |
| ☐ Begging | ☐ Other__________________ | ☐ N/A | |

| **If the primary caregiver works (earning or non-earning), main place of work?** *Select one* | | |
|---|---|---|
| ☐In/around home (where child lives) | ☐ Away for <4 hours per day | ☐Away >4 hours but comes home daily |
| ☐Away > 8h a day but returns home daily | ☐Away >1 day, comes home weekly | ☐ Away comes home, less than weekly |
| ☐Primary caregiver lives and works away | ☐ Don't know | ☐ N/A |

| **The person primarily providing financial support to this child is this child's:** *Select one* | | | |
|---|---|---|---|
| ☐ Biologic Mother | ☐ Biologic Father | ☐ Stepfather | ☐ Stepmother |
| ☐ Grandparent | ☐ Sibling ≥18 years old | ☐ Sibling <18 years old | ☐ Aunt/Uncle/Cousin |
| ☐ More than one person responsible, unclear | ☐ Unsupported / care home | ☐ Other -specify________________ | |

| **Person responsible for providing financial support to child, place of usual residence**? *Select one* | |
|---|---|
| ☐ Always sleeps at home | ☐ Sleeps away but returns weekly |
| ☐ Sleeps away for > two months per year | ☐ Works and lives abroad, contact with child once a year or less |
| ☐ Sleeps away but return monthly or less often | ☐ Don't know |
| ☐ Other_______________________________________ | ☐ N/A (e.g. care home, unsupported) |

| **What is the Father or person responsible for providing financial support to child source of income**?<br>*Select one. If the primary carer is also the person providing financial support do not complete this section.* | | | |
|---|---|---|---|
| ☐ Farmer | ☐ Business/trader | ☐ Labourer | ☐ Domestic work |
| ☐ Other private sector employment | ☐ Public sector employment | ☐ Retired with pension income | |
| ☐ Begging ☐None ☐ Unknown | ☐ Other ____________________ | ☐ N/A | |

| **Substitute Care:** *Who usually looks after child when primary caretaker is working or away? Select all that apply* | | | | | | |
|---|---|---|---|---|---|---|
| ☐ Not applicable, caregiver looks after child full time | | ☐ Not applicable, child accompanies caregiver to work | | | | |
| ☐ No substitute care, child left alone | | ☐ No substitute care / unclear | | ☐ Child in care home | | |
| ☐ Biological Mother | ☐ Biological Father | ☐ Sibling <18 years old | | ☐ Sibling ≥18 years old | | |
| ☐ Grandparent | ☐ Aunt/Uncle/Cousin | ☐ Childcare facility outside home | | ☐ Childminder/ day care at home | | |
| **How many days a week is the child in day care?** | | ☐ N/A | ☐ 1-2 | ☐ 3-4 | ☐ 5-6 | ☐ >6 |
| **How many hours per day is the child in day care?** | | ☐ N/A | ☐ 1-4h | ☐ 5-8h | ☐ 9-12h | ☐ >12h |
| **How many children are looked after at this day care?** | | ☐ <3 | ☐ 4-6 | ☐ 7-10 | ☐ >10 | ☐Unknown ☐ N/A |
| **How many of these are under 2y?** | | ☐ <3 | ☐ 4-6 | ☐ 7-10 | ☐ >10 | ☐Unknown ☐ N/A |
| **How many adults look after these children?** | | ☐ 1 | ☐2-4 | ☐5-10 | ☐ >10 | ☐ N/A |
| **Do you feel the day care is good?** | | ☐ Y | ☐ N | ☐ N/A | | |
| **Who provides food for the child at day care?** *Select one* | | | | | | |
| ☐ Caregiver provides food for the child | ☐ Day care provides food for the child | ☐ Someone else provides food for the child | ☐ Don't know | ☐ N/A | | |
| **Is feeding supervised / assisted at day care?** | ☐ Y | ☐ N | ☐ Unknown | ☐ N/A | | |

| **Household Food Security** (if child in care home include **children** in the care home only) | | | |
|---|---|---|---|
| **During the past 7 DAYS** has ANY member of the household missed a meal due to food shortage? | ☐ Y | ☐ N | ☐ Unknown |
| **During the past 4 WEEKS** | | | |
| Did you worry that your household would not have enough food? | ☐ Y | ☐ N | ☐ Unknown |
| Were any of your household unable to eat the kinds of food preferred because of a lack of resources? | ☐ Y | ☐ N | ☐ Unknown |
| Have any of your household had to eat a limited variety of food due to lack of resources? | ☐ Y | ☐ N | ☐ Unknown |
| Have any of your household eaten some foods that you really didn't want to eat because of lack of resources? | ☐ Y | ☐ N | ☐ Unknown |
| Have any of your household eaten fewer meals in a day because there was not enough food? | ☐ Y | ☐ N | ☐ Unknown |
| Did household members go to sleep at night hungry because there was not enough food? | ☐ Y | ☐ N | ☐ Unknown |
| Did you or your household members go a whole day and night without eating anything because there was not enough food? | ☐ Y | ☐ N | ☐ Unknown |

| Child Dietary Diversity |
|---|
| ***What does the child eat on a typical day?***<br>• *Ask this as an open question and select all that the caregiver mentions.*<br>• *Do not present the caregiver with this list.*<br>• *You may prompt the caregiver with open questions, e.g. What does your child usually eat for breakfast* |
| ☐ **Milk and Milk Products**: Fresh/fermented milk, cheese, yogurt, or other milk products |
| ☐ **Breast milk** |
| ☐ **Cereals and Cereal Products**: Maize, rice, pasta, porridge, bread, biscuits, millet, sorghum, wheat, locally available grains |
| ☐ **Fish and Sea Foods**: fresh or dried fish or shellfish |
| ☐ **Roots and Tubers**: potatoes, sweet potatoes, yams, cassava, or foods made from roots or wild roots and tubers |
| ☐ **Vegetables**: Cabbages, carrots, spinach, and any other locally available vegetables including wild vegetables |
| ☐ **Fruits**: Oranges, bananas, mangoes, avocados, apples, grapes etc |
| ☐ **Meats and Poultry**: Camel, beef, lamb, goat, rabbit, wild game, chicken or other birds, liver, kidney, heart or other organ meats or blood-based foods |
| ☐ **Eggs**: Hen or other bird eggs |
| ☐ **Pulses / Legumes / Nuts and Seeds**: Beans, peas, lentils, nuts, seeds or foods made from these |
| ☐ **Fats and Oils**: Oil, fats, ghee, margarine or butter added to food or used for cooking |
| ☐ **Sugars / Honey and Commercial Juices**: Sugar in tea, honey, sweetened soda, juices, chocolates, sweets or candies |
| ☐ **Miscellaneous**: Spices, unsweetened beverages |
| ☐ **UNKNOWN** |

| Feeding practices | |
|---|---|
| **How is food USUALLY given to the child? *Select one*** | |
| ☐ Fed by adult | ☐ Child feeds self, unsupervised |
| ☐ Child feeds self, supervised by adult | ☐ Fed from common plate or bowl |
| ☐ Child feeds self, supervised by older children | ☐ Child exclusively breastfed |
| ☐ Unknown | ☐ Other |

| Assessment of household wealth<br>(DHS 7 questionnaire. Please answer all questions, for all participants, including children in care homes) | | |
|---|---|---|
| **What is the main source of drinking water for members of your household?** *Choose one* | | |
| ☐ Piped water to dwelling | ☐ Cart with small tank | ☐ from vendor |
| ☐ Piped water to yard / plot | ☐ Tanker truck | ☐ Rainwater |
| ☐ Piped to neighbour | ☐ Bottled water | ☐ Stream/river/lake/pond/dam |
| ☐ Public tap/ Standpipe | ☐ Protected spring | ☐ Unknown |
| ☐ Protected well / borehole | ☐ Unprotected spring | |
| ☐ Unprotected well | ☐ Other | |
| **What is the MAIN source of water used by your household for other purposes such as cooking and handwashing? SELECT ONE ONLY** | | |
| ☐ Piped water to dwelling | ☐ Cart with small tank | ☐ Bought from vendor |
| ☐ Piped water to yard / plot | ☐ Tanker truck | ☐ Rainwater |
| ☐ Piped to neighbour | ☐ Bottled water | ☐ Stream/river/lake/pond/dam |
| ☐ Public tap/ Standpipe | ☐ Protected spring | ☐ Unknown |
| ☐ Protected well / borehole | ☐ Unprotected spring | |
| ☐ Unprotected well | ☐ Other | |

| | | |
|---|---|---|
| **How long does it take to get DRINKING water and come back? (State 0 if water supplied within home or compound)** | __ __ __minutes | ☐ Don't know |
| **In the past 2 weeks was the water from this source not available for at least one full day?** | ☐ Y ☐ N | ☐ Unknown |

| **Do you usually do anything to the water to make it safer to drink?** *Select all that apply* | | | |
|---|---|---|---|
| ☐ None | ☐ Bleach/ chlorine | ☐ Strain through a cloth | ☐ Let it stand and settle |
| ☐ Use water filter (ceramic/sand/composite etc) | ☐ Solar disinfection | ☐ Boil | ☐ Other |

| | | | |
|---|---|---|---|
| **What kind of toilet facility do members of your household usually use?** *Select one* | | | |
| ☐ Flush or pour flush toilet to piped sewer | ☐ Flush to septic tank | ☐ Ventilated improved pit latrine | |
| ☐ Flush to pit latrine | ☐ Flush to somewhere else | ☐ Open pit / Pit latrine without slab | |
| ☐ Flush don't know where | ☐ Composting toilet | ☐ Bucket toilet | |
| ☐ Pit latrine with slab | ☐ Hanging toilet / hanging latrine | ☐ No facility / bush/ field | |
| ☐ Unknown | | | |
| **Do you share this toilet facility with other households?** | ☐ Y | ☐ N | ☐ Unknown |
| **If Yes, including your own household, how many households use this toilet facility?** | Number if <10__ | ☐ >10 households | ☐ Unknown ☐ N/A |
| **Where is this toilet facility located?** | ☐ In own dwelling | ☐ In own yard / plot | ☐ Elsewhere |
| **How many rooms are there in the household for SLEEPING?** | ☐ **1** | ☐ **2** | ☐ **>2** |
| **What is the MAIN FLOOR material of the rooms in this household?** *Select one only* | | | |
| ☐ Cement | ☐ Earth/sand | ☐ Wood | |
| ☐ Dung | ☐ Lives on boat | ☐ Tiles | |
| ☐ Carpet | ☐ Other (specify)____________________ | ☐ Unknown | |
| **What is the MAIN WALL material of the rooms in this household?** *Select one only* | | | |
| ☐ Grass/straw/makuti | ☐ Stone | ☐ Wood | ☐ Unknown |
| ☐ Corrugated iron sheet/ Tin | ☐ Mud/wood | ☐ Brick/block | |
| ☐ Planks/shingles | ☐ No wall | ☐ Other (specify) __________________ | |
| **What is the MAIN ROOF material of the house in this household?** *Select one only* | | | |
| ☐ Grass/Thatch | ☐ Tiles/Asbestos sheets | ☐ Corrugated iron/ Tins | |
| ☐ Mud | ☐ Nylon papers/clothes | ☐ Concrete | |
| ☐ Other (specify) _________________________ | | ☐ Unknown | |
| **What is the MAIN cooking fuel used in this household?** *Select one only* | | | |
| ☐ Electricity | ☐ LPG /Natural gas/Biogas | ☐ Paraffin | |
| ☐ Coal / Lignite | ☐ Charcoal | ☐ Firewood | |
| ☐ Straw/shrubs/grass | ☐ Agricultural crop | ☐ Animal Dung | |
| ☐ No food cooked in household | ☐ Other (specify)_____________________ | ☐ Unknown | |
| **Do you have a separate room which is used as a kitchen?** | ☐ Y | ☐ N | ☐ Unknown |
| **Where is this household's cooking area located?** | | | |
| ☐ In the house ☐ Outdoors | ☐ In a separate building | ☐ Other ______________ | ☐ Unknown |

| | | | | |
|---|---|---|---|---|
| **Does this household own any livestock, herds, other farm animals or poultry** | | ☐ Y | ☐ N | ☐ Unknown |
| **If yes, how many of the following animals does this household own?** | | | | |
| Cows/bulls__ __ Sheep__ __<br>Horses/Donkeys/Mules__ __ Goats__ __<br>Chickens or Ducks__ __ Other ______________ number ___ ___ | | | | ☐ N/A |
| **Does any member of this this household own land?** | | ☐ Y | ☐ N | ☐ Unknown |
| **If "Yes" How many acres of land does this household own?** | | _____Acres | ☐ Unknown | ☐ N/A |
| **Does this household have a bank account?** | | ☐ Y | ☐ N | ☐ Unknown |
| **Does this household have electricity** | | ☐ Y | ☐ N | ☐ Unknown |
| **Does this household own a radio?** | | ☐ Y | ☐ N | ☐ Unknown |
| **Does this household own a television?** | | ☐ Y | ☐ N | ☐ Unknown |
| **Does this household own a computer?** | | ☐ Y | ☐ N | ☐ Unknown |
| **Does this household own a refrigerator?** | | ☐ Y | ☐ N | ☐ Unknown |
| **Does any member of this household own:** | | | | |
| **A watch** | | ☐ Y | ☐ N | ☐ Unknown |
| **A mobile phone?** | ☐ Y Standard phone | ☐ Y smartphone | ☐ N | ☐ Unknown |
| **An animal-drawn cart?** | | ☐ Y | ☐ N | ☐ Unknown |
| **A bicycle?** | | ☐ Y | ☐ N | ☐ Unknown |
| **A motorcycle / scooter?** | | ☐ Y | ☐ N | ☐ Unknown |
| **A car or truck?** | | ☐ Y | ☐ N | ☐ Unknown |
| **A boat with a motor?** | | ☐ Y | ☐ N | ☐ Unknown |

| | | | |
|---|---|---|---|
| **CRF Completed by (Initials) – to be signed when complete.**<br>*Do not sign if any fields are empty* | __ __ __ | Date<br>__ __/__ __/__ __ __ __<br>*D D / M M / Y Y Y Y* | Time<br>___ ___: ___ ___ |

END
