# Supplementary material for: Childhood Acute Illness and Nutrition (CHAIN) Network: a protocol for a multi-site prospective cohort study to identify modifiable risk factors for mortality among acutely ill children in Africa and Asia
Source: BMJ Open. 2019 May 5;9(5):e028454. doi: 10.1136/bmjopen-2018-028454 (PMC6502050; doi:10.1136/bmjopen-2018-028454)
Supplement: Author's manuscript [file bmjopen-2018-028454.draft_revisions.pdf]

BMJ Open is committed to open peer review. As part of this commitment we make the peer review history of every article we publish publicly available.

When an article is published we post the peer reviewers' comments and the authors' responses online. We also post the versions of the paper that were used during peer review. These are the versions that the peer review comments apply to.

The versions of the paper that follow are the versions that were submitted during the peer review process. They are not the versions of record or the final published versions. They should not be cited or distributed as the published version of this manuscript.

BMJ Open is an open access journal and the full, final, typeset and author-corrected version of record of the manuscript is available on our site with no access controls, subscription charges or pay-per-view fees (http://bmjopen.bmj.com).

If you have any questions on BMJ Open's open peer review process please email info.bmjopen@bmj.com

The Childhood Acute Illness and Nutrition Network Cohort Study

Journal:	*BMJ Open*
Manuscript ID	bmjopen-2018-028454
Article Type:	Protocol
Date Submitted by the Author:	08-Dec-2018

Complete List of Authors:	The Childhood Acute Illness and Nutrition (CHAIN), Network; University of Washington System, Global Health<br>Walson, Judd; University of Washington, Department of Global Health

Keywords:	Acute illness, hospitalisation, post-discharge mortality, malnutrition

SCHOLARONE™
Manuscripts

**Title:**	The Childhood Acute Illness and Nutrition Network Cohort Study
**Running head:**	CHAIN Cohort Study
**Authors:**	The Childhood Acute Illness and Nutrition (CHAIN) Network<br>* (authors listed in appendix as collaborators)
**Corresponding author (s)**	Judd Walson; walson@uw.edu ; +1 206 7443695<br>James A Berkley; JBerkley@kemri-wellcome.org; +254 720867011
**Affiliations:**	Centre for Tropical Medicine & Global Health, The University of Oxford, Oxford, UK; The Department of Global Health, The University of Washington, Seattle, USA; KEMRI-Wellcome Trust Research Programme, Kilifi, Kenya; The Childhood Acute Illness & Nutrition (CHAIN) Network
**Funded by:**	The Bill and Melinda Gates Foundation [OPP1131320]
**Trial registration**	Clinicaltrials.gov, NCT03208725
**Key words:**	Acute illness; malnutrition; post-discharge mortality; hospitalisation
**Word count:**	~4000

For peer review only

ABSTRACT

Introduction:

Children admitted to hospital in resource-poor settings remain at risk of both inpatient and post-discharge mortality. While known risk factors such as young age and nutritional status can identify children at risk, these factors do not provide clear mechanistic targets for intervention. The Childhood Acute Illness and Nutrition (CHAIN) Cohort Study aims to characterise biomedical and social risk factors for mortality in acutely ill children in hospital and after discharge to identify targeted interventions to reduce mortality.

Methods and analysis:

The CHAIN Network is undertaking a multisite, prospective, observational cohort enrolling children aged one week to two years at admission to hospitals at nine sites located in four African and two South Asian countries. CHAIN supports sites to provide care according to national and international guidelines. Enrolment is stratified by anthropometric status and children are followed throughout hospitalisation and for six months after discharge. Detailed clinical, demographic, anthropometric, laboratory and social exposures are assessed. Scheduled visits are conducted at 45, 90 and 180 days after discharge. Blood, stool and rectal swabs are collected at enrolment, hospital discharge, and follow-up. The primary outcome is inpatient or post-discharge death. Secondary outcomes include readmission to hospital and nutritional status after discharge. Cohort analysis will identify modifiable risks, children with distinct phenotypes, relationships between factors and mechanisms underlying poor outcomes that may be targets for intervention. A nested case-control study examining infectious, immunologic, metabolic, nutritional and other biological factors will be undertaken.

Ethics and dissemination:

This study protocol was reviewed and approved primarily by the Oxford Tropical Research Ethics Committee, and the institutional review boards of all partner sites. The study is being externally monitored. Results will be published in open access peer-reviewed scientific journals and presented to academic and policy stakeholders.

Trial Registration Number: NCT03208725.

Key words: Acute illness; malnutrition; post-discharge; mortality; hospitalisation; resource-poor; child survival; children; infants

For peer review only

ARTICLE SUMMARY

- The CHAIN cohort is a multi-country study collecting comprehensive data on clinical, laboratory, social, economic and behavioural exposures at multiple sites in Africa and Asia.
- Heterogeneity across sites (geography, rural/urban, varying HIV and malaria prevalence etc). increases generalisability and may help identify context-independent and dependent interventions.
- Strict harmonisation of procedures, processes and tools allows for comparability between sites.
- This study is deliberately focused on hospitalised children and is not designed to evaluate factors associated with pre-hospital care and timing of presentation to hospital.
- Bias may be introduced by loss-to-follow-up after hospital discharge if this occurs in more vulnerable children, or if participation in the study alters outcomes.

For peer review only

For peer review only - http://bmjopen.bmj.com/site/about/guidelines.xhtml

INTRODUCTION

Despite impressive global reductions in child mortality, more than five million children under age five die each year.(1) These deaths occur disproportionately in low and middle-income countries (LMICs).(2) Factors underlying mortality risk are complex and overlapping, including undernutrition, acute and chronic co-morbidities, lack of access to care, poor social support systems and poverty.(3, 4) For many vulnerable children, these factors contribute to an unstable health trajectory, with repeated episodes of illness interspersed with short periods of incomplete recovery (Figure 1). In most areas of the world, acutely ill children may not access health care during episodes of illness.(5) Of those that do, studies have shown that children who are hospitalised have dramatically higher risk of death both during hospitalisation and in the months following discharge than their community peers, even when guidelines for treatment and clinical follow up are followed.(6-9) Thus, contact with the health system resulting in hospitalisation serves as an indicator of vulnerability and a time-point where children are readily accessible for intervention.(10-13) While many interventions may be available in hospitals, there are no clear interventions proven to reduce post-discharge mortality. Undernutrition, HIV infection young age, and a history of repeated hospitalisation have emerged as important risk factors for post-discharge death.(3, 9, 14)

Figure 1: A model of unstable health trajectory of a child characterised by repeated illness and incomplete recovery

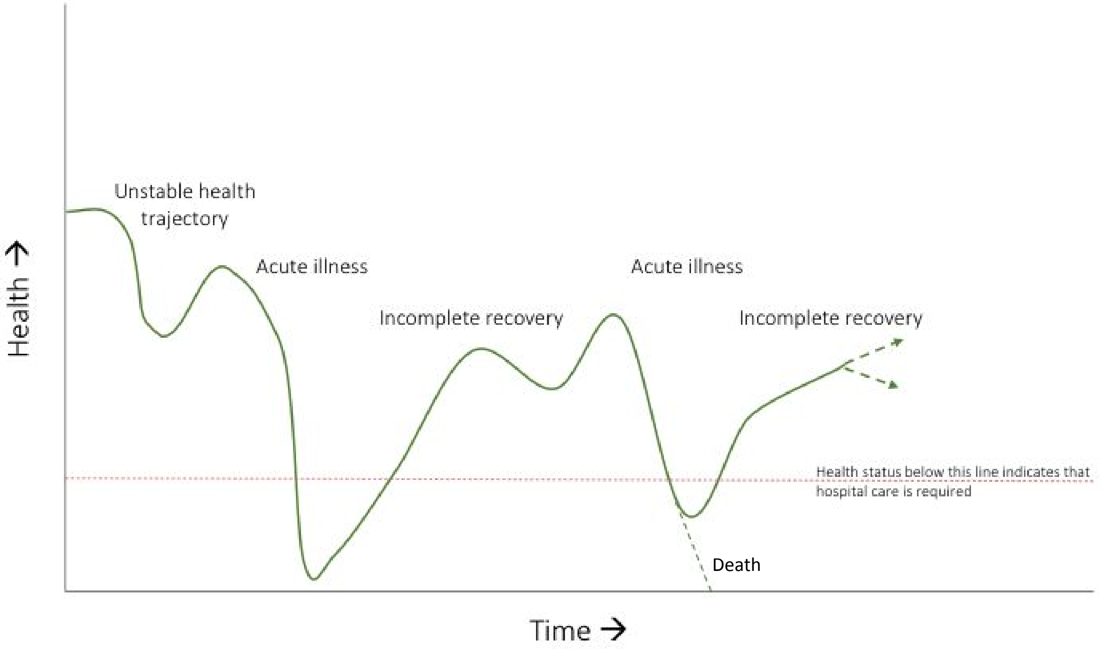

Approximately half of all childhood mortality is associated with one or more forms of undernutrition, typically identified using simple anthropometry.(2) Mortality rates among children with severe acute malnutrition (SAM) are markedly elevated compared to the general population,(15) but with effective treatment in the community without infectious complications, case fatality may be as low as 1-2%.(16, 17) However, when children with SAM are acutely ill, inpatient case fatality is often 15-20% in resource-poor countries, with many additional deaths occurring after discharge, even among children who appear to be recovering anthropometrically.(18) Although case fatality rates for children with moderate acute malnutrition (MAM) or stunting in hospital and after discharge have not been widely reported, MAM and stunting are more prevalent than SAM and may contribute to a greater number of child deaths.(2, 19)

Current guidelines for the clinical management of acutely ill children with SAM, MAM or stunting are not supported by strong evidence.(20-23) In addition, while measures of mid-upper arm circumference, weight-for-length and length-for-age can identify children as malnourished, anthropometry does not identify the mechanisms that link undernutrition to mortality nor its underlying causes. In typical settings outside humanitarian emergencies, it is unclear to what extent malnutrition is driven by factors other than food intake, including recurrent or chronic infection, intestinal or systemic inflammation, malabsorption, disability, social vulnerability, parental ill-health, maternal education and agency, lack of financial resources, poor social support and limited access to health care.(24) Identifying markers of vulnerability and the underlying mechanisms that drive mortality may offer opportunity to target interventions to improve outcomes in many low-resource settings. In addition, understanding relationships between biological and social risk factors may allow optimisation of intervention packages within specific groups to achieve the largest mortality reductions.

The Childhood Acute Illness and Nutrition Network (CHAIN) is a collaborative group of investigators working across nine sites in four African and two South Asian countries committed to improving child survival and optimising growth and development outcomes in low-resource settings. CHAIN is conducting a prospective cohort study with the objective of determining phenotypes of groups of children at highest risk, defining the mechanisms underlying such risk and identifying modifiable targets for intervention.

METHODS

Objectives

The CHAIN Network is conducting a prospective cohort study of approximately 4500 children aged one week to two years of age presenting to health care facilities with acute illness deemed severe enough to require admission to hospital at nine sites in six low- or middle-income countries. Children are followed throughout hospitalisation, discharge and for six months post-discharge. The general objective of the study is to characterise acutely ill children and their outcomes in hospital and after discharge in order to identify modifiable pathways leading to death. Using these data, the CHAIN network will prioritise interventions based on deliverability and potential impact.

Specific Objectives

1) To determine factors associated with i) mortality in hospital; ii) mortality after discharge; iii) readmission to hospital; and iv) poor nutritional recovery.

For peer review only - http://bmjopen.bmj.com/site/about/guidelines.xhtml

2) To determine differences in clinical, social, and pathophysiologic (e.g., metabolic, infectious, and immune) phenotypes between children of different nutritional status at admission, discharge and follow-up.
3) To identify modifiable risk factors and potential interventions to reduce mortality amongst vulnerable children.

The goal of the CHAIN Network is to identify interventions that can reduce child mortality beyond the current recommendations for standard of care. Hence, prior to participant enrolment, all study sites underwent a formal assessment of their capacity and adherence to national and international guidelines, including a survey of human resources, materials, equipment, pharmaceutical stock and laboratory capacity. All sites were provided with support to ensure that the minimum standard of care achieved was in line with national and international care guidelines. All sites participate in an audit and feedback cycle of guideline adherence using the individual patient data collected by CHAIN.

Locations

Participating sites include rural and urban hospitals in Bangladesh (icddr,b Dhaka Hospital and Matlab Hospital), Burkina Faso (Banfora Regional Referral Hospital), Kenya (Kilifi County Hospital, Mbagathi sub-County Hospital and Migori County Referral Hospital), Malawi (Queen Elizabeth Central Hospital), Pakistan (Civil Hospital, Karachi), and Uganda (Mulago Hospital).

Eligibility

Children aged seven days to 23 months who are admitted to hospital with an acute illness are eligible for enrolment. Prior to October 2018, the lower age limit of enrolment was 60 days.

Exclusion criteria are;

- Unable to tolerate oral feeds in the 48 hours prior to the onset of the current acute illness
- Underlying terminal illness that in the opinion of the treating physician is likely to lead to death within 6 months
- Diagnosed with a condition that in the opinion of the treating physician is likely to require surgery within 6 months
- Diagnosed chromosomal abnormality (syndromically or genetically diagnosed abnormality)
- Primary reason for admission is trauma or surgery
- Requiring immediate resuscitation at admission defined by on-going cardiac or pulmonary arrest or judged to be peri-arrest by the attending physician
- Previous enrolment in the study
- Sibling enrolled in the study
- Caregiver plans to move outside of the hospital catchment area within six months
- Caregiver is unwilling to attend study visits
- Lack of informed consent

Screening

Each site has nominated a day on which weekly recruitment begins. The first eligible child in each enrolment group admitted to the hospital is identified and approached for consent. This is repeated for every eligible child admitted subsequently until weekly targets are met for each enrolment stratum (see Table 1 below). These procedures aim to ensure that the study population adequately represents the spectrum of nutritional states observed over the recruitment period.

For peer review only - http://bmjopen.bmj.com/site/about/guidelines.xhtml

Potential participants are identified by clinical staff and brought to the attention of the study team who verify eligibility. If eligible, the study is explained to caregivers in their primary language and written informed consent to participate is sought. Caregivers who are unable to write are asked to provide a witnessed thumbprint. If consent is obtained, the child is enrolled and given a unique study number is allocated. If a child is deemed eligible but is too sick for consent to be immediately sought, study staff obtain verbal assent to collect both research and clinical samples at that time to avoid multiple needle insertions. If a caregiver who gave assent then chooses not to provide full written consent, all research data and samples are destroyed. Children classified as orphans or those living in alternative care homes are eligible for enrolment if an appropriate caregiver is present to provide consent on the child's behalf are eligible for enrolment.

Enrolment

Enrolment is stratified by nutritional status, aiming at a ratio of 2:2:1 (A: B: C) with group targets of 200 group A: 200 group B: 100 group C per site (Table 1).

Table 1: Enrolment groups by age and nutritional status

Group	Target N	Age >6 months	Age 1 - 6 months	Age < 1 month
A	1800	MUAC <11.5cm or kwashiorkor	MUAC <11cm or kwashiorkor	MUAC <9.5cm or kwashiorkor
B	1800	MUAC 11.5 to <12.5cm	MUAC 11 to <12cm	MUAC 9.5-10.4cm
C	900	MUAC ≧12.5cm	MUAC ≧12cm	MUAC ≧10.5cm

Mid-upper arm circumference (MUAC) was chosen as the optimal measure for participant selection as it is strongly associated with mortality, captures stunted children, varies less with dehydration than weight-based indices and is easily measured in sick children. (25, 26)

Procedures

All sites completed standardised training on variable definitions, identification of clinical signs, measurement of anthropometry, case report form (CRF) completion and data entry prior to starting the study, and training is repeated regularly. An independent study monitor (WESTAT) was hired to conduct site assessments to ensure harmonisation of study procedures across and between sites, as well as to ensure compliance with regulatory standards.

Upon satisfaction of the inclusion criteria and completion of informed consent forms, a unique study ID is allocated. Baseline data, including demographic and social information, a detailed clinical examination and measurement of vital signs, including pulse oximetry, are collected using the standardised CRF. Anthropometry is performed (head circumference, MUAC, weight, and length). At admission, biological samples, including blood (up to 5mL for research purposes), rectal swabs and faecal samples are obtained. All children are offered provider-initiated counselling and testing for HIV, and a malaria rapid diagnostic test is done. Results of investigations performed for clinical care (complete blood count (CBC), biochemistry, glucose or any other laboratory investigations collected) are abstracted and recorded for study purposes. After treatment is initiated, data on the child's diet, social circumstances and if the mother is present, maternal mental health screening is undertaken.

For peer review only - http://bmjopen.bmj.com/site/about/guidelines.xhtml

During admission, hospitalised children are reviewed daily and specific clinical features indicating illness progression and treatments are recorded on a structured daily CRF that is entered into the CHAIN database. In the event of death in hospital, a standard mortality audit questionnaire is completed by a designated member of the study team.

At discharge, the same clinical assessment as at admission, including anthropometry, is conducted and blood and faecal sampling are repeated. A home visit is conducted within three days of discharge. The location of the household is recorded by Global Positioning System (GPS) and CRFs are used to capture information on the number of people living in the homestead, access to clean water and improved sanitation, occupation, household assets, income and food security. Parents and legal guardians are also interviewed about their home and social situation, including challenges experienced when keeping their child and family healthy.

Follow-up procedures

Children are followed-up at 45, 90 and 180 days after discharge at the study facilities, irrespective of scheduled or unscheduled outpatient visits for medical or nutritional care. A standardised questionnaire ascertaining vital status, care-seeking and re-hospitalisation history and recent dietary intake (up to seven days before contact with the hospital) is collected and anthropometry, rectal swabs, faecal specimens and blood samples are repeated at each follow-up visit. Maternal mental health is screened again at day 45.

Children judged to have significant illness at any follow up visit or at the home visit are referred to an appropriate hospital, clinic or nutrition programme. The study staff share any test result relevant to the patient's care with the clinical management team and families.

Parents and legal guardians are asked to bring their child to the study clinic if they are concerned that the child is unwell. Financial reimbursement for transport and lost earnings at standard local rates is provided at the clinic visit. Study participants who are re-admitted to study hospitals undergo the standard clinical assessment delivered at enrolment. Participants who are admitted to hospitals other than the study site hospital have medical data abstracted onto standardised hospital re-admission forms. For deaths occurring outside the hospital, a verbal autopsy to evaluate the cause of death is completed within 28 days of study staff becoming aware of a death, using select questions from the WHO standard verbal autopsy tool.(27)

Community participants

To establish community norms, 125 children at each site living in the same community as hospitalised participants are recruited as community participants. absence of known untreated HIV or TB; no hospital admission in the 14 days prior to contact with the study team; and no previously participation in the study. The exclusion criteria listed for hospitalised children also apply to community participants.

At every site, one in four hospital participants has a child enrolled from their community. Selection of hospitalised participants is either every fourth participant, or, in periods when enrolment in hospital was lower, for example during health care workers strikes, one community participant was retrospectively enrolled for every second hospitalised participant. Community participants are identified randomly from the hospitalised participants home; a random number x (1-4) and direction (north, south, east, west) are generated using an online tool prior to visiting the home. Random number selection was done using a web-based application, Random Number Generator/ Picker.(28) Once at the home of the hospitalised participant the research team begin by visiting the xth house in the generated direction and attempt to obtain consent to enrol a child within the eligible

For peer review only - http://bmjopen.bmj.com/site/about/guidelines.xhtml

age range from that household. If not successful they continue in the same direction to the next x[th] house. This is repeated until a child is enrolled. Children with severe illness requiring hospital admission identified during community screening are referred for appropriate care.

When an eligible community participant is identified, their carer is given information about the study and invited to the study clinic for assessment. Following confirmation of informed consent, a clinical examination and anthropometry are completed and documented, and blood and stool samples collected as in the hospitalised children. Household and demographic questionnaires are also administered. Children in this group requiring non-urgent medical care receive basic treatment in the study clinic and/or are referred to appropriate treatment centres after enrolment procedures are completed. These children remain eligible for inclusion as community participants. Financial reimbursement for transport and lost earnings at standard local rates is provided to each carer at the clinic visit. No follow-up is done on community participants.

Specimen collection

To ensure comparability, standard operating procedures (SOPs) are followed at each site. Blood samples are collected at enrolment, each day of the hospitalisation, in the event of clinical deterioration (defined by the onset of a new Integrated Management of Childhood Illness (IMCI) danger sign), at discharge, at all follow-up points, and at any hospital readmission. Blood is collected into a BD Vacutainer Hemogard™ K_2EDTA (spray dried) and a red top with clot activator (spray dried)) at each time point and three dried blood spots on Whatman filter paper cards are prepared. At each time point, a complete blood count and clinical biochemistry, including sodium, potassium, calcium, magnesium, urea, creatinine, albumin, bilirubin, alanine aminotransferase, inorganic phosphate and alkaline phosphate are performed. Blood glucose, HIV testing (Alere Determine or Uni-Gold HIV) and malaria rapid diagnostics tests (CareStart HRP2/pLDH) are also performed on all children at enrolment and at additional time points if clinically indicated. Caregivers may refuse any sample collection during the study without being excluded from further follow-up. The schedule for blood collection is detailed in Table 2.

Stool collected directly or from child diapers (if fresh) is transferred into standardised stool collection pots by study staff, aliquoted and stored at -80˚C. Advanced pathogen detection using Taqman Array Card, sequencing, microbiome analysis, metabolomics and markers of enteric inflammation and dysfunction will be conducted on a subset of stool samples in the CHAIN nested case control study.

For peer review only

For peer review only - http://bmjopen.bmj.com/site/about/guidelines.xhtml

Table 2: Schedule of blood samples and tests performed at each collection time point

		ADMISSION	DISCHARGE	DAY 45	DAY 90	DAY 180	RE-ADMISSION
**Results used for care**	CBC	X	X	X	X	X	X
	Biochemistry	X					X
	Glucose	X					X
	Blood gas + lactate*	X					X
	Blood culture*	X					X
	Malaria RDT	X					X
	HIV test	X					
**Processed or stored for research assays only**	Dried blood Spot	X	X	X	X	X	X
	Serum + plasma for storage	X	X	X	X	X	X
	Whole blood for storage	X	X	X	X	X	X
	Functional immunology*	X	X	X	X	X	X
	PBMC extraction*	X	X	X	X	X	X
	Rectal swab & whole stool	X	X	X	X	X	X

* done at a subset of sites with capacity, thus these are sub-studies.

For peer review only - http://bmjopen.bmj.com/site/about/guidelines.xhtml

Data Management and Confidentiality

Data recorded on standardised CRFs are de-identified and entered in a secured central database and housed on servers in Nairobi with secure offsite backup. Prior to and after data entry, paper records are kept in a locked room with locked filing cabinet at each site, with access limited to investigators and study staff directly involved in data collection and entry. For de-identification, only participant initials are recorded and any potential identifier such as date of birth and GPS location are stored separately. Site principal investigators or their delegates conduct regular internal quality assurance on completion, data transfer and storage of the CRFs. A central data management system generates automated queries and review incoming data weekly. Any missing or implausible data is queried and site teams are given specific timeframe to resolve raised issues.

Analysis

Data from all Network sites will be combined as a single cohort. Outcomes will be classified as one or more of the following, the proportion of children with each endpoint described with 95% confidence intervals:

- Death at any time during the period of observation (primary endpoint)
- Death in hospital
- Death after discharge
- Readmitted to hospital within 180 days
- Nutritional status at 180 days
- Recovery as defined by survival without readmission or development of severe acute malnutrition during 180 days after discharge.

Primary cohort analysis - mortality

The hazard of mortality during the study will be calculated in multivariable survival models including i) clinical signs and symptoms; ii) anthropometric markers of wasting and stunting; iii) markers of organ function; iv) birth history and prior health; and v) maternal, household and social factors. The stratified design by location and nutritional category will be accounted for with multilevel fixed and random effects. Sub-group analyses will be conducted for inpatient and post-discharge mortality, including time-to-event survival analyses. Predictive algorithms will be built using methods such as classification and regression trees (CART), boosted regression trees and other machine learning methods. Mechanistic pathways will be interrogated using structured equation modelling, latent class analysis and other methods.

Secondary cohort analysis

Secondary endpoints are re-hospitalisation and the presence of SAM or MAM after 6 months. These will be examined using generalised linear models accounting for the competing risk of mortality. Growth trajectories post discharge will be compared to WHO standard growth curves and modelled in relation to risk factors and outcomes as panel data. Differences in mortality between sites will be examined in relation to case-mix and underlying risk factors such as HIV, and socio-economic factors.

For peer review only

Nested case-control study

A nested case control design will be utilised to investigate mechanisms with advanced laboratory testing on stored samples for efficiency. Cases will be defined as i) children who died during follow up and ii) children who are readmitted to hospital. Controls will be selected from children who survive to 6 months without readmission ("pure controls"), matched by site and nutritional strata to reflect the study design.

The primary analyses will estimate the association between the exposures of interest and the odds of poor outcomes during follow-up. The exposures of interest in these analyses will include plasma and faecal markers of intestinal dysfunction, systemic inflammation, systemic and enteric pathogens, 'maturity' and diversity of the enteric microbiome, proteomic and metabolomic markers of energy metabolism, and macro- and micronutrient status.

If mortality in the study is lower than expected (500 observed deaths), the primary case definition will be expanded to include children who were enrolled but were re-hospitalised post-discharge. Samples from these children may also be used in a sensitivity analysis to determine if associations with re-admission with severe illness ("near-miss") differ from those for mortality.

Sample size

Sample size calculations are based on expected events (deaths) in the cohort. We anticipate based on prior information from the sites that recruiting approximately 4,500 children in the specified strata will result in 500 deaths in this population. The sample size estimation is based on detecting differences in the proportion who die post-discharge between moderately malnourished and non-malnourished groups. Among non-malnourished children, we assumed an inpatient case fatality proportion of 5.0%, a cumulative post-discharge mortality of 2.5% compared to 7.5% inpatient case fatality among moderately malnourished children and allowing for 10% loss-to follow-up. A two-sided hypothesis that Ha: p2 ≠ p1, with an alpha of 0.05, a power of 80% is attained for a post-discharge case fatality of 4.8% or above.

For laboratory analyses on stored samples, a nested case control approach will compare cases (children who die) and controls (children with good recovery) in a 1:2 ratio matched by site and nutritional strata. We expect approximately 500 deaths will occur, with 1000 controls. For a two-sided hypothesis that Ha: p2 ≠ p1 at 80% power and a significance level of 0.05, this allows determination of an odds ratio of 1.8 for risk factors with a prevalence of 5% among controls, and of 1.4 for risk factors with a prevalence of 25%. Further analyses using a combined secondary endpoint of death and/or re-hospitalisation will ensure adequate power to detect risk factors with lower prevalence.

At each site, inclusion of 125 community participants will permit the calculation of descriptive percentages as integer values and estimates of community norms of continuous variables. (29) Across the whole study, 1,025 community participants will be recruited. For a two-sided hypothesis that Ha: p2 ≠ p1, at 80% power and a significance level of 0.05 and assuming clustering by site, this sample size allows determination of a prevalence ratio of 1.5 for risk factors with a prevalence of 5% among community participants, and of 1.25 for risk factors with a prevalence of 25%.

Study timeline

The CHAIN Cohort began enrolling on 20th November 2016 and participant recruitment and follow up is expected to occur through August 2019.

For peer review only - http://bmjopen.bmj.com/site/about/guidelines.xhtml

Potential challenges and limitations

The study is designed and powered to detect associations with mortality, the primary outcome. Prior data from other studies conducted at the CHAIN sites suggest that sufficient numbers of events will occur to achieve adequate power. However, an interim analysis will be conducted in 2018 to confirm that the cohort study is adequately powered to detect effects of covariates of interest. In the event that mortality or enrolment rates are not sufficient, we will include re-hospitalisations as a combined primary endpoint. This cohort study is being conducted in low-resource environments where the risk of civil, political or military disruption, and industrial action affecting hospitals are significant. The inclusion of multiple sites across a wide geographic range allows for some sites to strategically increase enrolment if other sites are unable to achieve planned targets.

Bias may occur if children lost to follow up are not representative of the study population, it is anticipated that non-attenders may be more vulnerable. There is also the risk of the Hawthorne effect where involvement in the study alters outcomes.

Ethics and dissemination of results

This study protocol was reviewed and approved by the Oxford Tropical Research Ethics Committee, UK; the Kenya Medical Research Institute, Kenya; the University of Washington and Oregon Health and Science University, USA; Makerere University School of Biomedical Sciences Research Ethics Committee and The Uganda National Council for Science and Technology, Uganda; Aga Khan University, Pakistan; the International Centre for Diarrheal Disease Research, Bangladesh; The University of Malawi; The University of Ouagadougou and Centre Muraz, Burkina Faso; the Hospital for Sick Children, Canada; and University of Amsterdam, The Netherlands. This study is registered with clinicaltrials.gov (NCT03208725).

Prior to project inception, key stakeholders at each site were engaged including those from relevant Ministries of Health, local academic institutions, hospitals hosting the study and the community engaged in the research. Community Advisory Boards have been assembled at each site. Study progress and results are shared with these Key Stakeholders as well as disseminated through workshops and written materials. The CHAIN Network has both ethics and policy advisory boards to providence guidance on how to tailor research activities and help disseminate key findings with enough reach and power to influence high level policy decisions.

Acknowledgments

The CHAIN Network thanks all the patients and their families for participating in this study. We acknowledge all leadership and staff at CHAIN hospital sites, and especially the clinical staff for sharing their resources and time to both care for CHAIN patients and collect pertinent data for the study.

Author Contributions

All authors contributed to the proposal and protocol development or implementation, which was led by Professors James Berkley and Judd Walson. Details of author contributions are listed in the supplementary appendix.

For peer review only

Funding Statement

This work was supported by the Bill and Melinda Gates Foundation, grant number OPP1131320.

Data Statement

At the end of the study, a technical appendix, statistical code, and restricted dataset will available on the CHAIN Network website: http://chainnetwork.org/.

Competing Interests Statement

None of the authors or study co-investigators have any competing interests to declare.

For peer review only

REFERENCES

1. WHO. Children: reducing mortality World Health Organization: WHO; 2018 [updated 9/18/2018; cited 2018 10/24/2018]. Available from: http://www.who.int/news-room/fact-sheets/detail/children-reducing-mortality.
2. Black RE, Victora CG, Walker SP, Bhutta ZA, Christian P, de Onis M, et al. Maternal and child undernutrition and overweight in low-income and middle-income countries. Lancet. 2013;382(9890):427-51.
3. Wiens MO, Pawluk S, Kissoon N, Kumbakumba E, Ansermino JM, Singer J, et al. Pediatric post-discharge mortality in resource poor countries: a systematic review. PloS one. 2013;8(6):e66698.
4. Man WD, Weber M, Palmer A, Schneider G, Wadda R, Jaffar S, et al. Nutritional status of children admitted to hospital with different diseases and its relationship to outcome in The Gambia, West Africa. Tropical medicine & international health : TM & IH. 1998;3(8):678-86.
5. CHERG. Underlying Causes of Child Death CHERG [cited 2018 10/24/2018]. Available from: http://cherg.org/projects/underlying_causes.html.
6. Kerac M, Bunn J, Seal A, Thindwa M, Tomkins A, Sadler K, et al. Probiotics and prebiotics for severe acute malnutrition (PRONUT study): a double-blind efficacy randomised controlled trial in Malawi. Lancet. 2009;374(9684):136-44.
7. Maitland K, Berkley JA, Shebbe M, Peshu N, English M, Newton CR. Children with severe malnutrition: can those at highest risk of death be identified with the WHO protocol? PLoS medicine. 2006;3(12):e500.
8. Talbert A, Thuo N, Karisa J, Chesaro C, Ohuma E, Ignas J, et al. Diarrhoea complicating severe acute malnutrition in Kenyan children: a prospective descriptive study of risk factors and outcome. PloS one. 2012;7(6):e38321.
9. Fergusson P, Tomkins A. HIV prevalence and mortality among children undergoing treatment for severe acute malnutrition in sub-Saharan Africa: a systematic review and meta-analysis. Trans R Soc Trop Med Hyg. 2009;103(6):541-8.
10. Kerac M, Bunn J, Chagaluka G, Bahwere P, Tomkins A, Collins S, et al. Follow-up of post-discharge growth and mortality after treatment for severe acute malnutrition (FuSAM study): a prospective cohort study. PloS one. 2014;9(6):e96030.
11. Moisi JC, Gatakaa H, Berkley JA, Maitland K, Mturi N, Newton CR, et al. Excess child mortality after discharge from hospital in Kilifi, Kenya: a retrospective cohort analysis. Bulletin of the World Health Organization. 2011;89(10):725-32, 32A.
12. Chisti MJ, Graham SM, Duke T, Ahmed T, Faruque AS, Ashraf H, et al. Post-discharge mortality in children with severe malnutrition and pneumonia in Bangladesh. PloS one. 2014;9(9):e107663.
13. Chang CY, Trehan I, Wang RJ, Thakwalakwa C, Maleta K, Deitchler M, et al. Children successfully treated for moderate acute malnutrition remain at risk for malnutrition and death in the subsequent year after recovery. J Nutr. 2013;143(2):215-20.
14. Heikens GT, Bunn J, Amadi B, Manary M, Chhagan M, Berkley JA, et al. Case management of HIV-infected severely malnourished children: challenges in the area of highest prevalence. Lancet. 2008;371(9620):1305-7.
15. Chen LC, Chowdhury AA, Huffman SL. Anthropometric assessment of energy-protein malnutrition and subsequent risk of mortality among preschool aged children. The American journal of clinical nutrition. 1980;33(8):1836-45.
16. Isanaka S, Langendorf C, Berthé F, Gnegne S, Li N, Ousmane N, et al. Routine amoxicillin for uncomplicated severe acute malnutrition in children. New England Journal of Medicine. 2016;374(5):444-53.
17. Isanaka S, Langendorf C, Berthe F, Gnegne S, Li N, Ousmane N, et al. Routine Amoxicillin for Uncomplicated Severe Acute Malnutrition in Children. The New England journal of medicine. 2016;374(5):444-53.
18. Ngari MM, Mwalekwa L, Timbwa M, Hamid F, Ali R, Iversen PO, et al. Changes in susceptibility to life-threatening infections after treatment for complicated severe malnutrition in Kenya. The American journal of clinical nutrition. 2018;107(4):626-34.
19. Bhutta ZA, Das JK, Rizvi A, Gaffey MF, Walker N, Horton S, et al. Evidence-based interventions for improvement of maternal and child nutrition: what can be done and at what cost? The lancet. 2013;382(9890):452-77.
20. WHO. Guideline: Updates on the management of severe acute malnutrition in infants and children. WHO Guidelines Approved by the Guidelines Review Committee. Geneva2013.
21. Picot J, Hartwell D, Harris P, Mendes D, Clegg AJ, Takeda A. The effectiveness of interventions to treat severe acute malnutrition in young children: a systematic review. Health Technol Assess. 2012;16(19):1-316.

For peer review only

For peer review only - http://bmjopen.bmj.com/site/about/guidelines.xhtml

22. WHO. Recommendations for Management of Common Childhood Conditions: Evidence for Technical Update of Pocket Book Recommendations: Newborn Conditions, Dysentery, Pneumonia, Oxygen Use and Delivery, Common Causes of Fever, Severe Acute Malnutrition and Supportive Care. Recommendations for Management of Common Childhood Conditions: Evidence for Technical Update of Pocket Book Recommendations: Newborn Conditions, Dysentery, Pneumonia, Oxygen Use and Delivery, Common Causes of Fever, Severe Acute Malnutrition and Supportive Care. WHO Guidelines Approved by the Guidelines Review Committee. Geneva 2012.
23. Angood C, McGrath M, Mehta S, Mwangome M, Lung'aho M, Roberfroid D, et al. Research Priorities to Improve the Management of Acute Malnutrition in Infants Aged Less Than Six Months (MAMI). PLoS medicine. 2015;12(4):e1001812.
24. Bhutta ZA, Berkley JA, Bandsma RHJ, Kerac M, Trehan I, Briend A. Severe childhood malnutrition. Nat Rev Dis Primers. 2017;3:17067.
25. Berkley J, Mwangi I, Griffiths K, Ahmed I, Mithwani S, English M, et al. Assessment of severe malnutrition among hospitalized children in rural Kenya: comparison of weight for height and mid upper arm circumference. JAMA : the journal of the American Medical Association. 2005;294(5):591-7.
26. Myatt M, Khara T, Collins S. A review of methods to detect cases of severely malnourished children in the community for their admission into community-based therapeutic care programs. Food and nutrition bulletin. 2006;27(3 Suppl):S7-23.
27. WHO. Verbal autopsy standards: the 2012 WHO verbal autopsy instrument. Geneva: WHO. 2012.
28. Hedges A. Random Number Generator / Picker 2008 [cited 2018 10/24/2018]. Available from: https://andrew.hedges.name/e/random/.
29. Horowitz GL, Altaie S, Boyd JC. Defining, establishing, and verifying reference intervals in the clinical laboratory; approved guideline: CLSI; 2010.

For peer review only

For peer review only

For peer review only - http://bmjopen.bmj.com/site/about/guidelines.xhtml

STROBE (Strengthening The Reporting of OBservational Studies in Epidemiology) Checklist

A checklist of items that should be included in reports of observational studies. You must report the page number in your manuscript where you consider each of the items listed in this checklist. If you have not included this information, either revise your manuscript accordingly before submitting or note N/A.

Note: An Explanation and Elaboration article discusses each checklist item and gives methodological background and published examples of transparent reporting. The STROBE checklist is best used in conjunction with this article (freely available on the Web sites of PLoS Medicine at http://www.plosmedicine.org/, Annals of Internal Medicine at http://www.annals.org/, and Epidemiology at http://www.epidem.com/). Information on the STROBE Initiative is available at www.strobe-statement.org.

Section and Item	Item No.	Recommendation	Reported on Page No.
**Title and Abstract**	1	(*a*) Indicate the study's design with a commonly used term in the title or the abstract	1
		(*b*) Provide in the abstract an informative and balanced summary of what was done and what was found	1
**Introduction**			
Background/Rationale	2	Explain the scientific background and rationale for the investigation being reported	
Objectives	3	State specific objectives, including any prespecified hypotheses	
**Methods**			
Study Design	4	Present key elements of study design early in the paper	4
Setting	5	Describe the setting, locations, and relevant dates, including periods of recruitment, exposure, follow-up, and data collection	5
Participants	6	(*a*) *Cohort study*—Give the eligibility criteria, and the sources and methods of selection of participants. Describe methods of follow-up	4-6
		Case-control study—Give the eligibility criteria, and the sources and methods of case ascertainment and control selection. Give the rationale for the choice of cases and controls	NA
		Cross-sectional study—Give the eligibility criteria, and the sources and methods of selection of participants	NA
		(*b*) *Cohort study*—For matched studies, give matching criteria and number of exposed and unexposed	NA
		Case-control study—For matched studies, give matching criteria and the number of controls per case	NA
Variables	7	Clearly define all outcomes, exposures, predictors, potential confounders, and effect modifiers. Give diagnostic criteria, if applicable	6-8

For peer review only

Section and Item	Item No.	Recommendation	Reported on Page No.
Data Sources/ Measurement	8*	For each variable of interest, give sources of data and details of methods of assessment (measurement). Describe comparability of assessment methods if there is more than one group	6-9
Bias	9	Describe any efforts to address potential sources of bias	NA
Study Size	10	Explain how the study size was arrived at	11
Quantitative Variables	11	Explain how quantitative variables were handled in the analyses. If applicable, describe which groupings were chosen and why	10-11
Statistical Methods	12	(*a*) Describe all statistical methods, including those used to control for confounding	10-11
		(*b*) Describe any methods used to examine subgroups and interactions	10-11
		(*c*) Explain how missing data were addressed	NA
		(*d*) *Cohort study*—If applicable, explain how loss to follow-up was addressed	10-11
		Case-control study—If applicable, explain how matching of cases and controls was addressed	NA
		Cross-sectional study—If applicable, describe analytical methods taking account of sampling strategy	NA
		(*e*) Describe any sensitivity analyses	NA
**Results**			
Participants	13*	(a) Report numbers of individuals at each stage of study—eg numbers potentially eligible, examined for eligibility, confirmed eligible, included in the study, completing follow-up, and analysed	NA
		(b) Give reasons for non-participation at each stage	NA
		(c) Consider use of a flow diagram	NA
Descriptive Data	14*	(a) Give characteristics of study participants (eg demographic, clinical, social) and information on exposures and potential confounders	NA
		(b) Indicate number of participants with missing data for each variable of interest	NA
		(c) *Cohort study*—Summarise follow-up time (eg, average and total amount)	NA
Outcome Data	15*	*Cohort study*—Report numbers of outcome events or summary measures over time	NA
		Case-control study—Report numbers in each exposure category, or summary measures of exposure	NA
		Cross-sectional study—Report numbers of outcome events or summary measures	NA

For peer review only

Section and Item	Item No.	Recommendation	Reported on Page No.
Main Results	16	(*a*) Give unadjusted estimates and, if applicable, confounder-adjusted estimates and their precision (eg, 95% confidence interval). Make clear which confounders were adjusted for and why they were included	NA
		(*b*) Report category boundaries when continuous variables were categorized	NA
		(*c*) If relevant, consider translating estimates of relative risk into absolute risk for a meaningful time period	NA
Other Analyses	17	Report other analyses done—eg analyses of subgroups and interactions, and sensitivity analyses	NA
**Discussion**			
Key Results	18	Summarise key results with reference to study objectives	NA
Limitations	19	Discuss limitations of the study, taking into account sources of potential bias or imprecision. Discuss both direction and magnitude of any potential bias	NA
Interpretation	20	Give a cautious overall interpretation of results considering objectives, limitations, multiplicity of analyses, results from similar studies, and other relevant evidence	NA
Generalisability	21	Discuss the generalisability (external validity) of the study results	NA
**Other Information**			
Funding	22	Give the source of funding and the role of the funders for the present study and, if applicable, for the original study on which the present article is based	13

*Give information separately for cases and controls in case-control studies and, if applicable, for exposed and unexposed groups in cohort and cross-sectional studies.

Once you have completed this checklist, please save a copy and upload it as part of your submission. DO NOT include this checklist as part of the main manuscript document. It must be uploaded as a separate file.

For peer review only

BMJ Open

The Childhood Acute Illness and Nutrition (CHAIN) Network: A Multi-site Prospective Cohort Study to Identify Modifiable Risk Factors for Mortality Among Acutely Ill Children in Africa and Asia

Journal:	*BMJ Open*
Manuscript ID	bmjopen-2018-028454.R1
Article Type:	Protocol
Date Submitted by the Author:	08-Feb-2019

Complete List of Authors:	The Childhood Acute Illness and Nutrition (CHAIN), Network; University of Washington System, Global Health<br>Walson, Judd; University of Washington, Department of Global Health

**Primary Subject Heading**:	Paediatrics
Secondary Subject Heading:	Global health, Epidemiology, Evidence based practice, Nutrition and metabolism, Public health
Keywords:	Acute illness, hospitalisation, post-discharge mortality, malnutrition

SCHOLARONE™ Manuscripts

**Title:**	The Childhood Acute Illness and Nutrition (CHAIN) Network: A Multi-site Prospective Cohort Study to Identify Modifiable Risk Factors for Mortality Among Acutely Ill Children in Africa and Asia.
**Running head:**	CHAIN Cohort Study
**Authors:**	The Childhood Acute Illness and Nutrition (CHAIN) Network<br>* (authors listed in appendix as collaborators)
**Corresponding author (s)**	Judd Walson; walson@uw.edu ; +1 206 7443695<br>James A Berkley; JBerkley@kemri-wellcome.org; +254 720867011
**Affiliations:**	Centre for Tropical Medicine & Global Health, The University of Oxford, Oxford, UK; The Department of Global Health, The University of Washington, Seattle, USA; KEMRI-Wellcome Trust Research Programme, Kilifi, Kenya; The Childhood Acute Illness & Nutrition (CHAIN) Network
**Funded by:**	The Bill and Melinda Gates Foundation [OPP1131320]
**Trial registration**	Clinicaltrials.gov, NCT03208725
**Key words:**	Acute illness; malnutrition; post-discharge mortality; hospitalisation
**Word count:**	~4000

For peer review only

ABSTRACT

Introduction:

Children admitted to hospital in resource-poor settings remain at risk of both inpatient and post-discharge mortality. While known risk factors such as young age and nutritional status can identify children at risk, these factors do not provide clear mechanistic targets for intervention. The Childhood Acute Illness and Nutrition (CHAIN) Cohort Study aims to characterise biomedical and social risk factors for mortality in acutely ill children in hospital and after discharge to identify targeted interventions to reduce mortality.

Methods and analysis:

The CHAIN Network is undertaking a multisite, prospective, observational cohort enrolling children aged one week to two years at admission to hospitals at nine sites located in four African and two South Asian countries. CHAIN supports sites to provide care according to national and international guidelines. Enrolment is stratified by anthropometric status and children are followed throughout hospitalisation and for six months after discharge. Detailed clinical, demographic, anthropometric, laboratory and social exposures are assessed. Scheduled visits are conducted at 45, 90 and 180 days after discharge. Blood, stool and rectal swabs are collected at enrolment, hospital discharge, and follow-up. The primary outcome is inpatient or post-discharge death. Secondary outcomes include readmission to hospital and nutritional status after discharge. Cohort analysis will identify modifiable risks, children with distinct phenotypes, relationships between factors and mechanisms underlying poor outcomes that may be targets for intervention. A nested case-control study examining infectious, immunologic, metabolic, nutritional and other biological factors will be undertaken.

Ethics and dissemination:

This study protocol was reviewed and approved primarily by the Oxford Tropical Research Ethics Committee, and the institutional review boards of all partner sites. The study is being externally monitored. Results will be published in open access peer-reviewed scientific journals and presented to academic and policy stakeholders.

Trial Registration Number: NCT03208725.

Key words: Acute illness; malnutrition; post-discharge; mortality; hospitalisation; resource-poor; child survival; children; infants

For peer review only

ARTICLE SUMMARY

- The CHAIN cohort is a multi-country study collecting comprehensive data on clinical, laboratory, social, economic and behavioural exposures at multiple sites in Africa and Asia.
- Heterogeneity across sites (geography, rural/urban, varying HIV and malaria prevalence etc). increases generalisability and may help identify context-independent and dependent interventions.
- Strict harmonisation of procedures, processes and tools allows for comparability between sites.
- This study is deliberately focused on hospitalised children and is not designed to evaluate factors associated with pre-hospital care and timing of presentation to hospital.
- Bias may be introduced by loss-to-follow-up after hospital discharge if this occurs in more vulnerable children, or if participation in the study alters outcomes.

For peer review only

INTRODUCTION

Despite impressive global reductions in child mortality, more than five million children under age five die each year.(1) These deaths occur disproportionately in low and middle-income countries (LMICs).(2) Factors underlying mortality risk are complex and overlapping, including undernutrition, acute and chronic co-morbidities, lack of access to care, poor social support systems and poverty.(3, 4) For many vulnerable children, these factors contribute to an unstable health trajectory, with repeated episodes of illness interspersed with short periods of incomplete recovery (Figure 1). In most areas of the world, acutely ill children may not access health care during episodes of illness.(5) Of those that do, studies have shown that children who are hospitalised have dramatically higher risk of death both during hospitalisation and in the months following discharge than their community peers, even when guidelines for treatment and clinical follow up are followed.(6-9) Thus, contact with the health system resulting in hospitalisation serves as an indicator of vulnerability and a time-point where children are readily accessible for intervention.(10-13) While many interventions may be available in hospitals, there are no clear interventions proven to reduce post-discharge mortality. Undernutrition, HIV infection young age, and a history of repeated hospitalisation have emerged as important risk factors for post-discharge death.(3, 9, 14)

Figure 1: A model of unstable health trajectory of a child characterised by repeated illness and incomplete recovery

Death

For peer review only

For peer review only - http://bmjopen.bmj.com/site/about/guidelines.xhtml

Approximately half of all childhood mortality is associated with one or more forms of undernutrition, typically identified using simple anthropometry.(2) Mortality rates among children with severe acute malnutrition (SAM) are markedly elevated compared to the general population,(15) but with effective treatment in the community without infectious complications, case fatality may be as low as 1-2%.(16, 17) However, when children with SAM are acutely ill, inpatient case fatality is often 15-20% in resource-poor countries, with many additional deaths occurring after discharge, even among children who appear to be recovering anthropometrically.(18) Although case fatality rates for children with moderate acute malnutrition (MAM) or stunting in hospital and after discharge have not been widely reported, MAM and stunting are more prevalent than SAM and may contribute to a greater number of child deaths.(2, 19)

Current guidelines for the clinical management of acutely ill children with SAM, MAM or stunting are not supported by strong evidence.(20-23) In addition, while measures of mid-upper arm circumference, weight-for-length and length-for-age can identify children as malnourished, anthropometry does not identify the mechanisms that link undernutrition to mortality nor its underlying causes. In typical settings outside humanitarian emergencies, it is unclear to what extent malnutrition is driven by factors other than food intake, including recurrent or chronic infection, intestinal or systemic inflammation, malabsorption, disability, social vulnerability, parental ill-health, maternal education and agency, lack of financial resources, poor social support and limited access to health care.(24) Identifying markers of vulnerability and the underlying mechanisms that drive mortality may offer opportunity to target interventions to improve outcomes in many low-resource settings. In addition, understanding relationships between biological and social risk factors may allow optimisation of intervention packages within specific groups to achieve the largest mortality reductions.

The Childhood Acute Illness and Nutrition Network (CHAIN) is a collaborative group of investigators working across nine sites in four African and two South Asian countries committed to improving child survival and optimising growth and development outcomes in low-resource settings. CHAIN is conducting a prospective cohort study with the objective of determining phenotypes of groups of children at highest risk, defining the mechanisms underlying such risk and identifying modifiable targets for intervention.

METHODS

Objectives

The CHAIN Network is conducting a prospective cohort study of approximately 4500 children aged one week to two years of age presenting to health care facilities with acute illness deemed severe enough to require admission to hospital at nine sites in six low- or middle-income countries. Children are followed throughout hospitalisation, discharge and for six months post-discharge. The general objective of the study is to characterise acutely ill children and their outcomes in hospital and after discharge in order to identify modifiable pathways leading to death. Using these data, the CHAIN network will prioritise interventions based on deliverability and potential impact.

Specific Objectives

1) To determine factors associated with i) mortality in hospital; ii) mortality after discharge; iii) readmission to hospital; and iv) poor nutritional recovery.

For peer review only - http://bmjopen.bmj.com/site/about/guidelines.xhtml

2) To determine differences in clinical, social, and pathophysiologic (e.g., metabolic, infectious, and immune) phenotypes between children of different nutritional status at admission, discharge and follow-up.
3) To identify modifiable risk factors and potential interventions to reduce mortality amongst vulnerable children.

The goal of the CHAIN Network is to identify interventions that can reduce child mortality beyond the current recommendations for standard of care. Hence, prior to participant enrolment, all study sites underwent a formal assessment of their capacity and adherence to national and international guidelines, including a survey of human resources, materials, equipment, pharmaceutical stock and laboratory capacity. All sites were provided with support to ensure that the minimum standard of care achieved was in line with national and international care guidelines. All sites participate in an audit and feedback cycle of guideline adherence using the individual patient data collected by CHAIN.

Locations

Participating sites include rural and urban hospitals in Bangladesh (icddr,b Dhaka Hospital and Matlab Hospital), Burkina Faso (Banfora Regional Referral Hospital), Kenya (Kilifi County Hospital, Mbagathi sub-County Hospital and Migori County Referral Hospital), Malawi (Queen Elizabeth Central Hospital), Pakistan (Civil Hospital, Karachi), and Uganda (Mulago Hospital).

Eligibility

Children aged seven days to 23 months who are admitted to hospital with an acute illness are eligible for enrolment. Prior to October 2018, the lower age limit of enrolment was 60 days.

Exclusion criteria are;

- Unable to tolerate oral feeds in the 48 hours prior to the onset of the current acute illness
- Underlying terminal illness that in the opinion of the treating physician is likely to lead to death within 6 months
- Diagnosed with a condition that in the opinion of the treating physician is likely to require surgery within 6 months
- Diagnosed chromosomal abnormality (syndromically or genetically diagnosed abnormality)
- Primary reason for admission is trauma or surgery
- Requiring immediate resuscitation at admission defined by on-going cardiac or pulmonary arrest or judged to be peri-arrest by the attending physician
- Previous enrolment in the study
- Sibling enrolled in the study
- Caregiver plans to move outside of the hospital catchment area within six months
- Caregiver is unwilling to attend study visits
- Lack of informed consent

Screening

Each site has nominated a day on which weekly recruitment begins. The first eligible child in each enrolment group admitted to the hospital is identified and approached for consent. This is repeated for every eligible child admitted subsequently until weekly targets are met for each enrolment stratum (see Table 1 below). Although this is a pseudo-random approach, all the procedures aim

For peer review only

to ensure that the study population adequately represents the spectrum of nutritional states observed over the recruitment period.

Potential participants are identified by clinical staff and brought to the attention of the study team who verify eligibility. If eligible, the study is explained to caregivers in their primary language and written informed consent to participate is sought. Caregivers who are unable to write are asked to provide a witnessed thumbprint. If consent is obtained, the child is enrolled and given a unique study number is allocated. If a child is deemed eligible but is too sick for consent to be immediately sought, study staff obtain verbal assent to collect both research and clinical samples at that time to avoid multiple needle insertions. If a caregiver who gave assent then chooses not to provide full written consent, all research data and samples are destroyed. Children classified as orphans or those living in alternative care homes are eligible for enrolment if an appropriate caregiver is present to provide consent on the child's behalf are eligible for enrolment.

Enrolment

Enrolment is stratified by nutritional status, aiming at a ratio of 2:2:1 (A: B: C) with group targets of 200 group A: 200 group B: 100 group C per site (Table 1).

Table 1: Enrolment groups by age and nutritional status

Group	Target N	Age >6 months	Age 1 - 6 months	Age < 1 month
A	1800	MUAC <11.5cm or kwashiorkor	MUAC <11cm or kwashiorkor	MUAC <9.5cm or kwashiorkor
B	1800	MUAC 11.5 to <12.5cm	MUAC 11 to <12cm	MUAC 9.5-10.4cm
C	900	MUAC ≧12.5cm	MUAC ≧12cm	MUAC ≧10.5cm

Mid-upper arm circumference (MUAC) was chosen as the optimal measure for participant selection as it is strongly associated with mortality, captures stunted children, varies less with dehydration than weight-based indices and is easily measured in sick children. (25, 26)

Procedures

All sites completed standardised training on variable definitions, identification of clinical signs, measurement of anthropometry, case report form (CRF) completion and data entry prior to starting the study, and training is repeated regularly. An independent study monitor (WESTAT) was hired to conduct site assessments to ensure harmonisation of study procedures across and between sites, as well as to ensure compliance with regulatory standards. Results from WESTAT's monitoring visits are for internal purposes and will be made available to the principal investigators at each site, the study funder and the CHAIN Network leadership and coordination teams.

Upon satisfaction of the inclusion criteria and completion of informed consent forms, a unique study ID is allocated. Baseline data, including demographic and social information, a detailed clinical examination and measurement of vital signs, including pulse oximetry, are collected using the standardised CRF. Anthropometry is performed (head circumference, MUAC, weight, and length). At admission, biological samples, including blood (up to 5mL for research purposes), rectal swabs and faecal samples are obtained. All children are offered provider-initiated counselling and testing

For peer review only - http://bmjopen.bmj.com/site/about/guidelines.xhtml

for HIV, and a malaria rapid diagnostic test is done. Results of investigations performed for clinical care (complete blood count (CBC), biochemistry, glucose or any other laboratory investigations collected) are abstracted and recorded for study purposes. After treatment is initiated, data on the child's diet, social circumstances and if the mother is present, maternal mental health screening is undertaken. Other data on maternal characteristics collected include maternal MUAC, height, weight and demographic data. Additional maternal variables are listed in the study's enrolment CRF which is included as a supplementary file.

During admission, hospitalised children are reviewed daily and specific clinical features indicating illness progression and treatments are recorded on a structured daily CRF that is entered into the CHAIN database. In the event of death in hospital, a standard mortality audit questionnaire is completed by a designated member of the study team.

At discharge, the same clinical assessment as at admission, including anthropometry, is conducted and blood and faecal sampling are repeated.

Follow-up procedures

A home visit is conducted within three days of discharge. The location of the household is recorded by Global Positioning System (GPS) and CRFs are used to capture information on the number of people living in the homestead, access to clean water and improved sanitation, occupation, household assets, income and food security. Parents and legal guardians are also interviewed about their home and social situation, including challenges experienced when keeping their child and family healthy.

Children are followed-up again at 45, 90 and 180 days after discharge at the study facilities, irrespective of scheduled or unscheduled outpatient visits for medical or nutritional care. A standardised questionnaire ascertaining vital status, care-seeking and re-hospitalisation history and recent dietary intake (up to seven days before contact with the hospital) is collected and anthropometry, rectal swabs, faecal specimens and blood samples are repeated at each follow-up visit. Maternal mental health is screened again at day 45.

Children judged to have significant illness at any follow up visit or at the home visit are referred to an appropriate hospital, clinic or nutrition programme. The study staff share any test result relevant to the patient's care with the clinical management team and families.

Parents and legal guardians are asked to bring their child to the study clinic if they are concerned that the child is unwell. Financial reimbursement for transport and lost earnings at standard local rates is provided at the clinic visit. Study participants who are re-admitted to study hospitals undergo the standard clinical assessment delivered at enrolment. Participants who are admitted to hospitals other than the study site hospital have medical data abstracted onto standardised hospital re-admission forms. For deaths occurring outside the hospital, a verbal autopsy to evaluate the cause of death is completed within 28 days of study staff becoming aware of a death, using select questions from the WHO standard verbal autopsy tool.(27)

Community participants

To establish community norms, 125 children at each site living in the same community as hospitalised participants are recruited as community participants. Absence of known untreated HIV or TB; no hospital admission in the 14 days prior to contact with the study team; and no previously

For peer review only - http://bmjopen.bmj.com/site/about/guidelines.xhtml

participation in the study. The exclusion criteria listed for hospitalised children also apply to community participants.

At every site, one in four hospital participants has a child enrolled from their community. Selection of hospitalised participants is either every fourth participant, or, in periods when enrolment in hospital was lower, for example during health care workers strikes, one community participant was retrospectively enrolled for every second hospitalised participant. Community participants are identified randomly from the hospitalised participants home; a random number x (1-4) and direction (north, south, east, west) are generated using an online tool prior to visiting the home. Random number selection was done using a web-based application, Random Number Generator/ Picker.(28) Once at the home of the hospitalised participant the research team begin by visiting the xth house in the generated direction and attempt to obtain consent to enrol a child within the eligible age range from that household. If not successful they continue in the same direction to the next x^{th} house. This is repeated until a child is enrolled. Children with severe illness requiring hospital admission identified during community screening are referred for appropriate care.

When an eligible community participant is identified, their carer is given information about the study and invited to the study clinic for assessment. Following confirmation of informed consent, a clinical examination and anthropometry are completed and documented, and blood and stool samples collected as in the hospitalised children. Household and demographic questionnaires are also administered. Children in this group requiring non-urgent medical care receive basic treatment in the study clinic and/or are referred to appropriate treatment centres after enrolment procedures are completed. These children remain eligible for inclusion as community participants. Financial reimbursement for transport and lost earnings at standard local rates is provided to each carer at the clinic visit. No follow-up is done on community participants.

Specimen collection

To ensure comparability, standard operating procedures (SOPs) are followed at each site. Blood samples are collected at enrolment, each day of the hospitalisation, in the event of clinical deterioration (defined by the onset of a new Integrated Management of Childhood Illness (IMCI) danger sign), at discharge, at all follow-up points, and at any hospital readmission. Blood is collected into a BD Vacutainer Hemogard™ K_2EDTA (spray dried) and a red top with clot activator (spray dried)) at each time point and three dried blood spots on Whatman filter paper cards are prepared. At each time point, a complete blood count and clinical biochemistry, including sodium, potassium, calcium, magnesium, urea, creatinine, albumin, bilirubin, alanine aminotransferase, inorganic phosphate and alkaline phosphate are performed. Blood glucose, HIV testing (Alere Determine or Uni-Gold HIV) and malaria rapid diagnostics tests (CareStart HRP2/pLDH) are also performed on all children at enrolment and at additional time points if clinically indicated. Caregivers may refuse any sample collection during the study without being excluded from further follow-up. The schedule for blood collection is detailed in Table 2.

Stool collected directly or from child diapers (if fresh) is transferred into standardised stool collection pots by study staff, aliquoted and stored at -80˚C. Advanced pathogen detection using Taqman Array Card, sequencing, microbiome analysis, metabolomics and markers of enteric inflammation and dysfunction will be conducted on a subset of stool samples in the CHAIN nested case control study.

For peer review only - http://bmjopen.bmj.com/site/about/guidelines.xhtml

Table 2: Schedule of blood samples and tests performed at each collection time point

		ADMISSION	DISCHARGE	DAY 45	DAY 90	DAY 180	RE-ADMISSION
**Results used for care**	CBC	X	X	X	X	X	X
	Biochemistry	X					X
	Glucose	X					X
	Blood gas + lactate*	X					X
	Blood culture*	X					X
	Malaria RDT	X					X
	HIV test	X					
**Processed or stored for research assays only**	Dried blood Spot	X	X	X	X	X	X
	Serum + plasma for storage	X	X	X	X	X	X
	Whole blood for storage	X	X	X	X	X	X
	Functional immunology*	X	X	X	X	X	X
	PBMC extraction*	X	X	X	X	X	X
	Rectal swab & whole stool	X	X	X	X	X	X

* done at a subset of sites with capacity, thus these are sub-studies.

For peer review only - http://bmjopen.bmj.com/site/about/guidelines.xhtml

Data Management and Confidentiality

Data recorded on standardised CRFs are de-identified and entered in a secured central database and housed on servers in Nairobi with secure offsite backup. Prior to and after data entry, paper records are kept in a locked room with locked filing cabinet at each site, with access limited to investigators and study staff directly involved in data collection and entry. For de-identification, only participant initials are recorded and any potential identifier such as date of birth and GPS location are stored separately. Site principal investigators or their delegates conduct regular internal quality assurance on completion, data transfer and storage of the CRFs. A central data management system generates automated queries and review incoming data weekly. Any missing or implausible data is queried and site teams are given specific timeframe to resolve raised issues.

Analysis

Data from all Network sites will be combined as a single cohort. Outcomes will be classified as one or more of the following, the proportion of children with each endpoint described with 95% confidence intervals:

- Death at any time during the period of observation (primary endpoint)
- Death in hospital
- Death after discharge
- Readmitted to hospital within 180 days
- Nutritional status at 180 days
- Recovery as defined by survival without readmission or development of severe acute malnutrition during 180 days after discharge.

Primary cohort analysis - mortality

The hazard of mortality during the study will be calculated in multivariable survival models including i) clinical signs and symptoms; ii) anthropometric markers of wasting and stunting; iii) markers of organ function; iv) birth history and prior health; and v) maternal, household and social factors. The stratified design by location and nutritional category will be accounted for with multilevel fixed and random effects. Sub-group analyses will be conducted for inpatient and post-discharge mortality, including time-to-event survival analyses. Predictive algorithms will be built using methods such as classification and regression trees (CART), boosted regression trees and other machine learning methods. Mechanistic pathways will be interrogated using structured equation modelling, latent class analysis and other methods.

Secondary cohort analysis

Secondary endpoints are re-hospitalisation and the presence of SAM or MAM after 6 months. These will be examined using generalised linear models accounting for the competing risk of mortality. Growth trajectories post discharge will be compared to WHO standard growth curves and modelled in relation to risk factors and outcomes as panel data. Differences in mortality between sites will be examined in relation to case-mix and underlying risk factors such as HIV, and socio-economic factors.

For peer review only - http://bmjopen.bmj.com/site/about/guidelines.xhtml

Nested case-control study

A nested case control design will be utilised to investigate mechanisms with advanced laboratory testing on stored samples for efficiency. Cases will be defined as i) children who died during follow up and ii) children who are readmitted to hospital. Controls will be selected from children who survive to 6 months without readmission ("pure controls"), matched by site and nutritional strata to reflect the study design.

The primary analyses will estimate the association between the exposures of interest and the odds of poor outcomes during follow-up. The exposures of interest in these analyses will include plasma and faecal markers of intestinal dysfunction, systemic inflammation, systemic and enteric pathogens, 'maturity' and diversity of the enteric microbiome, proteomic and metabolomic markers of energy metabolism, and macro- and micronutrient status.

If mortality in the study is lower than expected (500 observed deaths), the primary case definition will be expanded to include children who were enrolled but were re-hospitalised post-discharge. Samples from these children may also be used in a sensitivity analysis to determine if associations with re-admission with severe illness ("near-miss") differ from those for mortality.

Sample size

Sample size calculations are based on expected events (deaths) in the cohort. We anticipate based on prior information from the sites that recruiting approximately 4,500 children in the specified strata will result in 500 deaths in this population. The sample size estimation is based on detecting differences in the proportion who die post-discharge between moderately malnourished and non-malnourished groups. Among non-malnourished children, we assumed an inpatient case fatality proportion of 5.0%, a cumulative post-discharge mortality of 2.5% compared to 7.5% inpatient case fatality among moderately malnourished children and allowing for 10% loss-to follow-up. A two-sided hypothesis that Ha: $p2 \neq p1$, with an alpha of 0.05, a power of 80% is attained for a post-discharge case fatality of 4.8% or above.

For laboratory analyses on stored samples, a nested case control approach will compare cases (children who die) and controls (children with good recovery) in a 1:2 ratio matched by site and nutritional strata. We expect approximately 500 deaths will occur, with 1000 controls. For a two-sided hypothesis that Ha: $p2 \neq p1$ at 80% power and a significance level of 0.05, this allows determination of an odds ratio of 1.8 for risk factors with a prevalence of 5% among controls, and of 1.4 for risk factors with a prevalence of 25%. Further analyses using a combined secondary endpoint of death and/or re-hospitalisation will ensure adequate power to detect risk factors with lower prevalence.

At each site, inclusion of 125 community participants will permit the calculation of descriptive percentages as integer values and estimates of community norms of continuous variables. (29) Across the whole study, 1,025 community participants will be recruited. For a two-sided hypothesis that Ha: $p2 \neq p1$, at 80% power and a significance level of 0.05 and assuming clustering by site, this sample size allows determination of a prevalence ratio of 1.5 for risk factors with a prevalence of 5% among community participants, and of 1.25 for risk factors with a prevalence of 25%.

Study timeline

The CHAIN Cohort began enrolling on 20th November 2016 and participant recruitment and follow up is expected to occur through August 2019.

For peer review only - http://bmjopen.bmj.com/site/about/guidelines.xhtml

Potential challenges and limitations

The study is designed and powered to detect associations with mortality, the primary outcome. Prior data from other studies conducted at the CHAIN sites suggest that sufficient numbers of events will occur to achieve adequate power. However, an interim analysis will be conducted in 2019 to confirm that the cohort study is adequately powered to detect effects of covariates of interest. In the event that mortality or enrolment rates are not sufficient, we will include re-hospitalisations as a combined primary endpoint. This cohort study is being conducted in low-resource environments where the risk of civil, political or military disruption, and industrial action affecting hospitals are significant. The inclusion of multiple sites across a wide geographic range allows for some sites to strategically increase enrolment if other sites are unable to achieve planned targets.

Bias may occur if children lost to follow up are not representative of the study population, it is anticipated that non-attenders may be more vulnerable. There is also the risk of the Hawthorne effect where involvement in the study alters outcomes.

Ethics and dissemination of results

This study protocol was reviewed and approved by the Oxford Tropical Research Ethics Committee, UK; the Kenya Medical Research Institute, Kenya; the University of Washington and Oregon Health and Science University, USA; Makerere University School of Biomedical Sciences Research Ethics Committee and The Uganda National Council for Science and Technology, Uganda; Aga Khan University, Pakistan; the International Centre for Diarrheal Disease Research, Bangladesh; The University of Malawi; The University of Ouagadougou and Centre Muraz, Burkina Faso; the Hospital for Sick Children, Canada; and University of Amsterdam, The Netherlands. This study is registered with clinicaltrials.gov (NCT03208725).

Prior to project inception, key stakeholders at each site were engaged including those from relevant Ministries of Health, local academic institutions, hospitals hosting the study and the community engaged in the research. Community Advisory Boards have been assembled at each site. Study progress and results are shared with these Key Stakeholders as well as disseminated through workshops and written materials. The CHAIN Network has both ethics and policy advisory boards to providence guidance on how to tailor research activities and help disseminate key findings with enough reach and power to influence high level policy decisions.

Patient and Public Involvement

The investigators who form the CHAIN Network leadership have extensive experience in the design and conduct of trials focused on improving growth and survival in children in Africa and Asia. The study was designed specifically to reflect patient priorities (child survival, reductions in hospitalization, improved growth. Working closely with Community Advisory Boards at each site, as well as with a robust social science team of researchers in Kenya and Bangladesh, patient priorities, experience and preferences were carefully considered in the design and development of the protocol and data collection instruments. The research questions were also discussed with a number of internal CHAIN Network and external experts in child survival at a convening organized by the funder. Caregivers were included in the Community Advisory Boards to provide input and into the conduct of the study. At the conclusion of the study, results will be disseminated to participants via seminars and outreach events led by the site PIs and the Community Advisory Boards at each site.

For peer review only - http://bmjopen.bmj.com/site/about/guidelines.xhtml

Acknowledgments

The CHAIN Network thanks all the patients and their families for participating in this study. We acknowledge all leadership and staff at CHAIN hospital sites, and especially the clinical staff for sharing their resources and time to both care for CHAIN patients and collect pertinent data for the study.

Author Contributions

Judd Walson and the CHAIN Network participated in proposal and protocol development.

Funding Statement

This work was supported by the Bill and Melinda Gates Foundation, grant number OPP1131320.

Data Statement

At the end of the study, a technical appendix, statistical code, and restricted dataset will available on the CHAIN Network website: http://chainnetwork.org/.

Competing Interests Statement

None of the authors or study co-investigators have any competing interests to declare.

For peer review only

REFERENCES

1. WHO. Children: reducing mortality World Health Organization: WHO; 2018 [updated 9/18/2018; cited 2018 10/24/2018]. Available from: http://www.who.int/news-room/fact-sheets/detail/children-reducing-mortality.
2. Black RE, Victora CG, Walker SP, Bhutta ZA, Christian P, de Onis M, et al. Maternal and child undernutrition and overweight in low-income and middle-income countries. Lancet. 2013;382(9890):427-51.
3. Wiens MO, Pawluk S, Kissoon N, Kumbakumba E, Ansermino JM, Singer J, et al. Pediatric post-discharge mortality in resource poor countries: a systematic review. PloS one. 2013;8(6):e66698.
4. Man WD, Weber M, Palmer A, Schneider G, Wadda R, Jaffar S, et al. Nutritional status of children admitted to hospital with different diseases and its relationship to outcome in The Gambia, West Africa. Tropical medicine & international health : TM & IH. 1998;3(8):678-86.
5. CHERG. Underlying Causes of Child Death CHERG [cited 2018 10/24/2018]. Available from: http://cherg.org/projects/underlying_causes.html.
6. Kerac M, Bunn J, Seal A, Thindwa M, Tomkins A, Sadler K, et al. Probiotics and prebiotics for severe acute malnutrition (PRONUT study): a double-blind efficacy randomised controlled trial in Malawi. Lancet. 2009;374(9684):136-44.
7. Maitland K, Berkley JA, Shebbe M, Peshu N, English M, Newton CR. Children with severe malnutrition: can those at highest risk of death be identified with the WHO protocol? PLoS medicine. 2006;3(12):e500.
8. Talbert A, Thuo N, Karisa J, Chesaro C, Ohuma E, Ignas J, et al. Diarrhoea complicating severe acute malnutrition in Kenyan children: a prospective descriptive study of risk factors and outcome. PloS one. 2012;7(6):e38321.
9. Fergusson P, Tomkins A. HIV prevalence and mortality among children undergoing treatment for severe acute malnutrition in sub-Saharan Africa: a systematic review and meta-analysis. Trans R Soc Trop Med Hyg. 2009;103(6):541-8.
10. Kerac M, Bunn J, Chagaluka G, Bahwere P, Tomkins A, Collins S, et al. Follow-up of post-discharge growth and mortality after treatment for severe acute malnutrition (FuSAM study): a prospective cohort study. PloS one. 2014;9(6):e96030.
11. Moisi JC, Gatakaa H, Berkley JA, Maitland K, Mturi N, Newton CR, et al. Excess child mortality after discharge from hospital in Kilifi, Kenya: a retrospective cohort analysis. Bulletin of the World Health Organization. 2011;89(10):725-32, 32A.
12. Chisti MJ, Graham SM, Duke T, Ahmed T, Faruque AS, Ashraf H, et al. Post-discharge mortality in children with severe malnutrition and pneumonia in Bangladesh. PloS one. 2014;9(9):e107663.
13. Chang CY, Trehan I, Wang RJ, Thakwalakwa C, Maleta K, Deitchler M, et al. Children successfully treated for moderate acute malnutrition remain at risk for malnutrition and death in the subsequent year after recovery. J Nutr. 2013;143(2):215-20.
14. Heikens GT, Bunn J, Amadi B, Manary M, Chhagan M, Berkley JA, et al. Case management of HIV-infected severely malnourished children: challenges in the area of highest prevalence. Lancet. 2008;371(9620):1305-7.
15. Chen LC, Chowdhury AA, Huffman SL. Anthropometric assessment of energy-protein malnutrition and subsequent risk of mortality among preschool aged children. The American journal of clinical nutrition. 1980;33(8):1836-45.
16. Isanaka S, Langendorf C, Berthé F, Gnegne S, Li N, Ousmane N, et al. Routine amoxicillin for uncomplicated severe acute malnutrition in children. New England Journal of Medicine. 2016;374(5):444-53.
17. Isanaka S, Langendorf C, Berthe F, Gnegne S, Li N, Ousmane N, et al. Routine Amoxicillin for Uncomplicated Severe Acute Malnutrition in Children. The New England journal of medicine. 2016;374(5):444-53.
18. Ngari MM, Mwalekwa L, Timbwa M, Hamid F, Ali R, Iversen PO, et al. Changes in susceptibility to life-threatening infections after treatment for complicated severe malnutrition in Kenya. The American journal of clinical nutrition. 2018;107(4):626-34.
19. Bhutta ZA, Das JK, Rizvi A, Gaffey MF, Walker N, Horton S, et al. Evidence-based interventions for improvement of maternal and child nutrition: what can be done and at what cost? The lancet. 2013;382(9890):452-77.
20. WHO. Guideline: Updates on the management of severe acute malnutrition in infants and children. WHO Guidelines Approved by the Guidelines Review Committee. Geneva2013.
21. Picot J, Hartwell D, Harris P, Mendes D, Clegg AJ, Takeda A. The effectiveness of interventions to treat severe acute malnutrition in young children: a systematic review. Health Technol Assess. 2012;16(19):1-316.

For peer review only

For peer review only - http://bmjopen.bmj.com/site/about/guidelines.xhtml

22. WHO. Recommendations for Management of Common Childhood Conditions: Evidence for Technical Update of Pocket Book Recommendations: Newborn Conditions, Dysentery, Pneumonia, Oxygen Use and Delivery, Common Causes of Fever, Severe Acute Malnutrition and Supportive Care. Recommendations for Management of Common Childhood Conditions: Evidence for Technical Update of Pocket Book Recommendations: Newborn Conditions, Dysentery, Pneumonia, Oxygen Use and Delivery, Common Causes of Fever, Severe Acute Malnutrition and Supportive Care. WHO Guidelines Approved by the Guidelines Review Committee. Geneva 2012.

23. Angood C, McGrath M, Mehta S, Mwangome M, Lung'aho M, Roberfroid D, et al. Research Priorities to Improve the Management of Acute Malnutrition in Infants Aged Less Than Six Months (MAMI). PLoS medicine. 2015;12(4):e1001812.

24. Bhutta ZA, Berkley JA, Bandsma RHJ, Kerac M, Trehan I, Briend A. Severe childhood malnutrition. Nat Rev Dis Primers. 2017;3:17067.

25. Berkley J, Mwangi I, Griffiths K, Ahmed I, Mithwani S, English M, et al. Assessment of severe malnutrition among hospitalized children in rural Kenya: comparison of weight for height and mid upper arm circumference. JAMA : the journal of the American Medical Association. 2005;294(5):591-7.

26. Myatt M, Khara T, Collins S. A review of methods to detect cases of severely malnourished children in the community for their admission into community-based therapeutic care programs. Food and nutrition bulletin. 2006;27(3 Suppl):S7-23.

27. WHO. Verbal autopsy standards: the 2012 WHO verbal autopsy instrument. Geneva: WHO. 2012.

28. Hedges A. Random Number Generator / Picker 2008 [cited 2018 10/24/2018]. Available from: https://andrew.hedges.name/e/random/.

29. Horowitz GL, Altaie S, Boyd JC. Defining, establishing, and verifying reference intervals in the clinical laboratory; approved guideline: CLSI; 2010.

For peer review only

For peer review only - http://bmjopen.bmj.com/site/about/guidelines.xhtml

Collaborators

Full Name	Affiliations	Role
Berkley, JA	KEMRI/Wellcome Trust Research Programme, Kilifi, Kenya.<br><br>Center for Tropical Medicine and Global Health, University of Oxford, Oxford, United Kingdom.	Network PI
Walson, JL	Departments of Global Health, Medicine, Paediatrics and Epidemiology, University of Washington, Seattle, Washington, USA.	Network PI
Diallo, AH	Department of Public Health, Faculty of Health Sciences, University of Ouagadougou I Pr Joseph KI-ZERBO, Burkina Faso;<br><br>Department of Public Health, Centre MURAZ Research Institute, Ministry of Health, Bobo-Dioulasso, Burkina Faso.	Site PI/Co-Investigator
Shahid, ASMSB	Nutrition and Clinical Services Division (NCSD), International Centre for Diarrhoeal Disease Research, Bangladesh (icddr,b), Dhaka, Bangladesh.	Co-Investigator
Gwela, A	KEMRI/Wellcome Trust Research Programme, Kilifi, Kenya	Co-Investigator
Saleem, A	Department of Pediatrics and Child Health, The Aga Khan University, Karachi, 74800, Pakistan	Site PI/Co-Investigator
Asad, A	Department of Pediatrics and Child Health, The Aga Khan University, Karachi, 74800, Pakistan	Co-Investigator
Tigoi, CC	KEMRI/Wellcome Trust Research Programme, Kilifi, Kenya	Co-Investigator
Bourdon, C	The Hospital for Sick Children, Department of Translational Medicine, Toronto, Canada	Co-Investigator
Lancioni, CL	Department of Pediatrics, Oregon Health and Science University, Portland, OR, USA	Co-Investigator
Denno, DM	Department of Pediatrics, School of Medicine, University of Washington, Seattle, WA. USA.<br><br>Department of Global Health and Department of Health Services, School of Public Health, University of Washington, Seattle, WA. USA.	Co-Investigator
Mangale, DI	Department of Global Health, University of Washington, Seattle, WA. USA.	Co-Investigator
Mupere, E	Department of Paediatrics and Child Health, College of Health Sciences, Makerere University, Kampala, Uganda.	Site PI/Co-Investigator
Tickell, KD	Departments of Global Health, and Epidemiology, University of Washington, Seattle, Washington, USA.	Co-Investigator
Mwangome, MK	KEMRI/Wellcome Trust Research Programme, Kilifi, Kenya	Co-Investigator
Chisti, MJ	Nutrition and Clinical Services Division (NCSD), International Centre for Diarrhoeal Disease Research, Bangladesh (icddr,b), Dhaka, Bangladesh.	Site PI/Co-Investigator
Ngari, MM	KEMRI/Wellcome Trust Research Programme, Kilifi, Kenya.	Co-Investigator
Ngao, NM	KEMRI/Wellcome Trust Research Programme, Kilifi, Kenya	Co-Investigator
Sukhtankar, P	KEMRI/Wellcome Trust Research Programme, Kilifi, Kenya	Co-Investigator
Bandsma, RHJ	Centre for Global Child Health, Hospital for Sick Children, Toronto, Ontario Canada.<br><br>Division of Gastroenterology, Hepatology and Nutrition, Hospital for Sick Children, Toronto, Ontario Canada.	Site PI/Co-Investigator

For peer review only - http://bmjopen.bmj.com/site/about/guidelines.xhtml

	Department of Nutritional Sciences, Faculty of Medicine, University of Toronto, Toronto, Ontario Canada.<br><br>Department of Biomedical Sciences, College of Medicine, University of Malawi, Blantyre, Malawi.	
Molyneux, S	KEMRI Centre for Geographic Medicine Research - Coast, and Wellcome Trust Research Programme, Nairobi, Kenya.<br><br>Centre for Tropical Medicine, University of Oxford, Oxford, UK.	Co-Investigator
Ahmed, T	Nutrition and Clinical Services Division (NCSD), International Centre for Diarrhoeal Disease Research, Bangladesh (icddr,b), Dhaka, Bangladesh.	Site PI/Co-Investigator
Voskuijl, W	Department of Paediatrics and Child Health, College of Medicine, University of Malawi, Blantyre, Malawi.<br><br>Department of Biomedical Sciences, College of Medicine, University of Malawi, Blantyre, Malawi.	Site PI/Co-Investigator

For peer review only

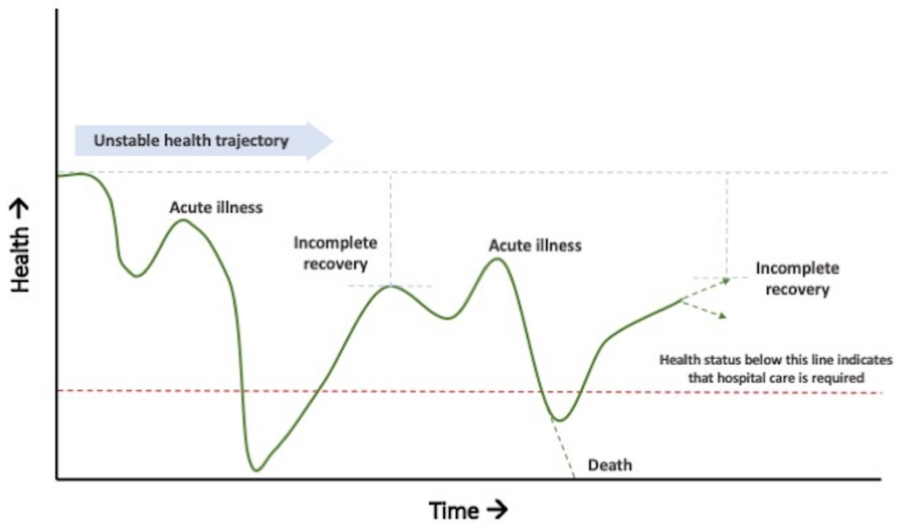

Figure 1: A model of unstable health trajectory of a child characterised by repeated illness and incomplete recovery

277x191mm (300 x 300 DPI)

CHAIN Enrolment CRF v1.63
CHAIN Number [1][0] [0][0][3] [ ][ ][ ]

Eligibility Checklist		
**Age between 2 months and before 2nd birthday**	Y	N - ineligible
**Being admitted to hospital because of acute illness**	Y	N- ineligible
**Parent or guardian able and available to consent**	Y	N- ineligible
**Able to feed orally in usual state of health**	Y	N- ineligible
**Known congenital syndrome**	Y- ineligible	N
**Cleft palate**	Y- ineligible	N
**Known congenital cardiac disease**	Y- ineligible	N
**Known terminal illness e.g. cancer**	Y- ineligible	N
**Admission for surgery, or likely to require surgery within 6m**	Y- ineligible	N
**Admission for trauma?**	Y- ineligible	N
**Sibling enrolled in study**	Y- ineligible	N
**Previously enrolled**	Y- ineligible	N

Part 1

Admission to Hospital and Study Enrolment					
**DATE arrived at the hospital**	__ __/__ __/__ __ __ __ D D / M M / Y Y Y Y	**TIME arrived at the hospital**	__ __: __ __ *24h Clock*	☐ Arrival time unknown	
**DATE of enrolment** i.e. date consented and seen by research team	__ __/__ __/__ __ __ __ D D / M M / Y Y Y Y	**TIME of enrolment**	__ __: __ __ *24h Clock*	**Sex**	☐ Male ☐ Female
**DOB**	__ __/__ __/__ __ __ __ D D / M M / Y Y Y Y	**Is the DOB:**	☐ True ☐ Estimated*	**Child's Initials**	___ ___ ___
**Brought into hospital by:** *Select all that apply*	☐ Mother	☐ Father	☐ Grandparent	☐ Aunt/Uncle	
	☐ Sibling <18	☐ Sibling >18	☐ Carer (care home)	☐ Other__________	

**if DOB is estimated, and the day is uncertain, write '15' for DD*

Presenting Complaints

☐ Fever / Hotness of body
☐ Difficulty breathing
☐ Cough<14 days
☐ Cough>14days
☐ Poor feeding/ Weight loss
☐ Rash/ skin lesion
☐ Vomiting
☐ Diarrhoea <14 days
☐ Diarrhoea >14 days
☐ Blood in stool
☐ Developmental delay
☐ Other *(only one complaint, if not covered by above options)*
____
☐ Lethargy
☐ Convulsions
☐ Altered consciousness
☐ Not feeding
☐ Body swelling / limb swelling/ Oedema

For peer review only - http://bmjopen.bmj.com/site/about/guidelines.xhtml

CHAIN Enrolment CRF v1.63

CHAIN Number [1][0] [0][0][3] [ ][ ][ ]

**Initial Observations** (to be taken at time of examination by research team)			
**Axillary temperature**	____ ____. ____ °C	**Respiratory rate** *Count for 1 minute*	____ ____ ____/minute
**Heart rate** *Count for 1 minute*	____ ____ ____/minute		
**SaO2** *To be taken from finger or toe using pulse oximeter*	____ ____ ____ % *Leave blank if unrecordable*	☐ Measured in Oxygen ☐ Measured in Room Air	☐ Unrecordable

Anthropometry					
**Weight** *to be taken using SECA scales for CHAIN study*	___ ___ . ___ ___kg		**Length** *to be taken using SECA 416 infantometer provided for CHAIN study*	Measurer 1	____ ____. ____ cm
				Measurer 2	____ ____ . ____ cm
**MUAC** *To be taken using MUAC tape for CHAIN study*	Measurer 1	____ ____ . ____ cm	**Head circumference** *To be taken using CHAIN measuring tape*	Measurer 1	____ ____. _____cm
	Measurer 2	____ ____ . ____ cm		Measurer 2	____ ____. _____cm
**Oedema**	☐ None ☐ + ☐ ++ ☐ +++		**Initials**	Measurer 1 ___ ___ ___	Measurer 2 ___ ___ ___

NB: If the child is unwell the Length and Head Circumference can be taken at a later time.

Current Health	
**Previously admitted to hospital.** *Include other hospitals / health centres. Select 1*	☐ No ☐ < 1 week ago ☐ 1 weeks-1month ago ☐ >1month ago
**Any medication last 7 days.** *Select all that apply*	☐ No medication ☐ Antibiotic ☐ Antimalarial ☐Traditional<br>☐ Deworming ☐ Vitamin ☐ Paracetamol or Ibuprofen<br>☐ Yes, but unknown ☐Other
**Urine volume in last 24hrs?** *Select 1*	☐ Not passing urine ☐ Less than normal ☐ Normal or greater ☐ Unknown

For peer review only - http://bmjopen.bmj.com/site/about/guidelines.xhtml

CHAIN Enrolment CRF v1.63

CHAIN Number [1][0] [0][0][3] [ ][ ][ ]

Examination				
Examination should be performed by CHAIN study clinician trained in clinical examination of children, and able to formulate a diagnosis based on clinical history and findings. Refer to Clinical Examination SOP				
**Airway** *(select one)*	☐ **Clear**	☐ Needs active support	☐ Obstructed/Stridor	
**Breathing** *(select all that apply)*	☐ **Normal – no concerns**, (move to circulation)			
	☐ Central cyanosis	☐ Nasal flaring	☐ Reduced air-entry	
	☐ Wheeze	☐ Acidotic Breathing	☐ Grunting	
	☐ Lower chest wall indrawing	☐ Crackles	☐ Dull to percussion<br>☐ Head nodding	
**Circulation:**				
**Cap Refill** (select one)	☐ >3s	☐ 2-3s	☐ <2s	
**Cold Peripheries**(select one)	☐ Shoulder	☐ Elbow	☐ Hand	☐ Warm peripheries
**Disability:**				
**Conscious level**(select one)	☐ **Alert**	☐ Voice	☐ Pain	☐ Unresponsive
**Fontanelle**(select one)	☐ **Normal**	☐ Bulging	☐ Sunken	☐ Not present
**Tone**(select one)	☐ **Normal**	☐ Hypertonic		☐ Hypotonic
**Posture**(select one)	☐ **Normal**	☐ Decorticate		☐ Decerebrate
**Activity**(select one)	☐ **Normal**	☐ Irritable/Agitated		☐ Lethargic
**Dehydration:**				
**Sunken eyes?**	☐ Y	☐ N		
**Skin pinch** *(select one)*	☐ >2 seconds	☐ <2 seconds	☐ Immediate	
**Drinking/Breastfeeding** *(Select one)*	☐ **Normal**	☐ Poorly	☐ Not drinking	☐ Eager / Thirsty
**Abdomen** *(select any that apply)*	☐ **Normal – no concerns**	☐ Distension	☐ Hepatomegaly	
	☐ Tenderness	☐ Splenomegaly	☐ Other abdominal mass	
**Signs of Rickets**	☐ **None**	☐ Wrist widening	☐ Rachitic rosary	☐ Swollen knees<br>☐ Bow legs<br>☐ Frontal bossing
**Jaundice** *(Select one)*	☐ **Not jaundiced**	☐ +	☐ ++	☐ +++
**ENT/Oral/Eyes** *(select any that apply)*	☐ Mouth Normal	☐ Ears Normal	☐ Eyes Normal	
	☐ Oral ulceration	☐ Pus from ear	☐ Conjunctivitis	
	☐ Oral candidiasis	☐ Tender swelling behind ear (mastoiditis)	☐ Eye discharge	
	☐ Stomatitis	☐ Lymphadenopathy	☐ Visual impairment	
**Skin** *(select any that apply)*	☐ Normal	☐ Hyperpigmentation	☐ Depigmentation	
	☐ Broken skin	☐ Dermatitis	☐ 'Flaky paint'	
	☐ Cellulitis	☐ Impetigo	☐ Pustules	
	☐ Vesicles	☐ Desquamation	☐ Macular or papular	
**Site of skin lesions.** *(select any that apply)*	☐ Not applicable (No rash)	☐ Trunk	☐ Face / scalp	☐ Legs
	☐ Palms / soles	☐ Buttocks	☐ Arms	☐ Perineum

For peer review only

For peer review only - http://bmjopen.bmj.com/site/about/guidelines.xhtml

CHAIN Enrolment CRF v1.63
CHAIN Number [1][0] [0][0][3] [ ][ ][ ]

Suspected Chronic Conditions			
Select confirmed, suspected or none for all conditions:	Confirmed *(diagnosed previously/ recorded)*	Suspected *(clinician's impression)*	None
**Cerebral palsy/neurological problem/ epilepsy**	☐	☐	☐
**Sickle Cell disease** *family history, crisis*	☐	☐	☐
**Thalassaemia**	☐	☐	☐
**Visual problem / Blindness** *Not fixing and following*	☐	☐	☐
**Losing weight or not gaining weight**	☐	☐	☐

TB Screening			
Known TB (on treatment)	Child has cough >14 days	Household contact has TB, or cough >14 days	Child has suspected extra-pulmonary TB
Y N	Y N	Y N	Y N

Feeding						
**Currently in outpatient nutrition program?** *Select one.*	☐ Supplementary *(corn soy blend, RUSF, khichuri, halwa)*	☐ Therapeutic *(RUTF, Plumpy-nut)*	☐ None			
**Has the child eaten these nutrition products in the last 3 days?**	☐ Supplementary	☐ Therapeutic	☐ None			
**Currently Breastfeeding?**	☐ Y ☐ N	**If yes is the child taking anything else (exclude medicine)?**	☐ Y ☐ N ☐ N/A			
**If NO breastfeeding at all, age stopped in months?** *(select one)*	☐ 0-3m	☐ 4-6m	☐ 7-12m	☐ >12m	☐ Unknown	☐ N/A
**What did the child receive other than breast milk in the first 3 days of life?** *Select all that apply* *Do not include medications e.g. ARV.*	☐ Sweetened/sugar water<br>☐ Fruit Juice<br>☐ Water<br>☐ Pure Honey	☐ Formula/powder milk<br>☐ Tea<br>☐ Porridge/pulp<br>☐ Glycerine	☐ Animal milk<br>☐ Other<br>☐ Gutthi / gripe water<br>☐ Nothing			

Vaccinations – *Ask carer or check book / card if available*						
**BCG scar**	☐ Yes ☐ No	**Rotavirus**	☐ Book	☐ Self report	☐ Not received	**Doses received:** 3 2 1 ☐ Unknown
**Measles**	☐ Book ☐ Self report ☐ Not received ☐ Unknown	**Pneumococcus**	☐ Book	☐ Self report	☐ Not received	**Doses received:** 3 2 1 ☐ Unknown
		**DTP/Penta**	☐ Book	☐ Self report	☐ Not received	**Doses received:** 3 2 1 ☐ Unknown
		**Polio**	☐ Book	☐ Self report	☐ Not received	☐ Unknown

For peer review only - http://bmjopen.bmj.com/site/about/guidelines.xhtml

CHAIN Enrolment CRF v1.63
CHAIN Number [1][0] [0][0][3] [ ][ ][ ]

CLINICIANS IMPRESSION OF RISK						
How likely does the clinical team think this child is to die during this admission? Select one						
☐ Almost certainly not	☐ Very unlikely	☐ Quite unlikely	☐ Unsure	☐ Quite likely	☐ Very likely	☐ Almost certainly

Immediate Clinical Investigations and HIV status						
**Malaria RDT** *circle result*	Positive		Negative		Not done	
**Blood glucose**	___ ___ . ___ mmol/L		**Time glucose measured**		___ ___:___ ___ *24h clock* ☐ Unknown	
**Urine Dipstick** *(can be done at any time during admission)* / **Urine sample stored?** Y N	Protein	Nitrites	Leucocytes	Blood	Ketones	Glucose
☐ Not done ☐ Bag ☐ Clean catch	None + ++ +++	Pos Neg	None + ++ +++	None + ++ +++	None + ++ +++	None + ++ +++

**HIV status known?**	☐ Yes, known PCR positive	☐ Yes, antibody positive, unknown PCR status	☐ Yes, known exposed, known PCR negative (children under 18m with PCR result SEEN BY RESEARCH TEAM. If not seen select below and perform HIV RDT	
	☐ No, known to be HIV exposed, but child untested		☐ No, child not tested, not known to be exposed	
**If child known HIV positive or exposed** – **On any ART?**	☐Y ☐N ☐ Unknown	**If on treatment,** ARV 1___ ARV 2___ ARV 3___	**If on prophylaxis** ☐Nevirapine prophylaxis only ☐AZT + NVP prophylaxis ☐Caregiver unsure	
**Co-trimoxazole** *select one*	☐ On prophylactic dose co-trimoxazole	☐On high dose co-trimoxazole	☐ Not on co-trimoxazole	☐Caregiver unsure
**If not known positive** – **HIV RDT now** *select one*	☐Reactive / positive	☐Non-Reactive / Negative	☐Declined	
	PCR sent: ☐Y ☐ N			

**HIV test offered to caregiver?**	☐Yes, Reactive	☐Yes, Non-reactive	☐Yes, but Declined	☐No, Caregiver is known positive	☐Missed	☐ N/A child in care home

**Did the mother have interventions or medication during delivery to prevent transmission of HIV to baby?**	☐Yes	☐No	☐Unknown

For peer review

For peer review only - http://bmjopen.bmj.com/site/about/guidelines.xhtml

CHAIN Enrolment CRF v1.63

CHAIN Number [1][0] [0][0][3] [ ][ ][ ]

INITIAL TREATMENT			
**Admitted to:** *select one*	☐ Admission to ward	☐ Admission to HDU	☐ Admission to ICU
**Date and time First antibiotics given**	__ __/ __ __/ __ __ __ __<br>*24h clock*	___ ___:___ ___	☐Not given
**Intravenous Antibiotics Given?**<br><br>☐ **Not given**	☐ Benzylpenicillin<br>☐ Co-amoxiclav/ Augmentin<br>☐ Ampicillin<br>☐ Levofloxacin<br>☐ Ceftazidime<br>☐ Other____	☐ Gentamicin<br>☐ Flu/Cloxacillin<br>☐ Amikacin<br>☐ Vancomycin<br>☐ Pivmecillinam	☐ Ceftriaxone / Cefotaxime<br>☐ Chloramphenicol<br>☐ Meropenem / Imipenem<br>☐ Metronidazole
**Oral Antibiotics Given?**<br><br>☐ **Not given**	☐ Amoxicillin<br>☐ Co-trimoxazole<br>☐ Cefalexin / cefaclor<br>☐ Penicillin<br>☐ Other _______________	☐ Erythromycin<br>☐ Metronidazole<br>☐ Co-amoxiclav / Augmentin<br>☐ Flucloxacillin	☐ Azithromycin<br>☐ Ciprofloxacin<br>☐ Nalidixic acid<br>☐ Levofloxacin

**Initial treatment given**<br>*First 6 hours.*<br>*Select any that apply.*<br>*For IV fluid bolus, and IV fluids specify type and volume in ml, and duration*		
	☐ IV Fluid Bolus	☐ IV Maintenance Fluids
	☐ Oxygen	☐ CPAP
	☐ IV Glucose ☐ Oral Glucose	☐ Warmth (heater, warmed fluids)
	☐ Blood transfusion	☐ Commercial F75
	☐ Phenobarbitone	☐ Commercial F100
	☐ Diazepam	☐ Locally prepared F75/ milk suji
	☐ Paracetamol	☐ Local prepared F100 / milk suji 100
	☐ Ibuprofen	☐ Expressed breast milk
	☐ Diclofenac	☐ Dilute F100/ dilute milk or formula
	☐ Salbutamol / atrovent / other bronchodilator	☐ Other milk/ formula/ feed<br>☐ RUTF
	☐ Prednisolone/ dexamethasone/ hydrocortisone	☐ Nasogastric tube
	☐ Adrenaline	☐ Multivitamin
	☐ Zinc	☐ Micronutrients
	☐ Folic acid	☐ Vitamin A
	☐ Antimalarial (any)	☐ Albendazole / deworming
	☐ ReSoMal	☐ Other<br>___________________________________
	☐ ORS	

For peer review only - http://bmjopen.bmj.com/site/about/guidelines.xhtml

CHAIN Enrolment CRF v1.63

CHAIN Number [1][0] [0][0][3] [ ][ ][ ]

Suspected Initial Diagnoses:

Clinical diagnosis should be based on examination and investigation findings.

Tick the three most likely diagnoses.

Respiratory	Infection	CNS
☐ LRTI/pneumonia	☐ Gastroenteritis	☐ Febrile convulsions
☐ Bronchiolitis	☐ Sepsis	☐ Epilepsy
☐ URTI	☐ Malaria	☐ Probable meningitis
☐ Pulmonary TB	☐ Extra pulmonary TB	☐ Other encephalopathy
☐ Otitis media	☐ Soft tissue infection	☐ Hydrocephalus
☐ Asthma	☐ UTI	☐ Developmental delay
**General**	☐ HIV related illness	☐ Cerebral palsy
☐ Anaemia	☐ Measles	**Other suspected diagnosis:**
☐ Sickle Cell Disease	☐ Varicella	☐ Other
☐ Thalassaemia	☐ Osteomyelitis	☐ Unknown
☐ Renal impairment	☐ Febrile illness unspecified	☐ Failed appetite test only
☐ Nephrotic syndrome	☐ Enteric fever	
☐ Nephritis		
☐ Liver dysfunction		
☐ Ileus		
☐ Congenital cardiac disease		

For peer review only

For peer review only - http://bmjopen.bmj.com/site/about/guidelines.xhtml

CHAIN Enrolment CRF v1.63
CHAIN Number [1][0] [0][0][3] [ ][ ][ ]

Admission Core Cohort Investigations and Sample Collection					
**CBC taken**	☐ Y	☐ N	**Plain Blood (serum)**	☐ Y	☐ N
**Clinical chemistry taken**	☐ Y	☐ N	**Blood spot taken**	☐ Y	☐ N
**EDTA 2ml blood taken**	☐ Y	☐ N	**Blood culture taken** *(if available at site)*	☐ Y BEFORE ABX<br>☐ Y AFTER ABX	☐ N
**EDTA 0.5ml blood taken**	☐ Y	☐ N	**Blood gas taken** *(if available at site)*	☐ Capillary<br>☐ Venous	☐ N
**Unable to take blood samples, why?**	☐ Difficult venepuncture	☐ Child uncooperative	☐ Parent refused	☐ Other	
**Rectal swabs taken**	☐ Y BEFORE ABX<br>☐ Y AFTER ABX	☐ N	Number taken ☐1 ☐2	Time taken___ ___: ___ ___	
**Stool sample**	Taken in first 24h?	☐ Y	☐ N	Time taken___ ___: ___ ___	

**Chest x-ray indicated** *(respiratory signs symptoms)*	☐ Yes, but too unwell	☐ Yes, done	☐ Indicated but not done, unclear	☐ Not indicated
**Lumbar puncture indicated** *(signs of meningitis documented)*	☐ Yes, but too unwell	☐ Yes, done		☐ Not indicated

**Blood Samples taken by (initials)**	__ __ __
**Rectal Swabs taken by (initials)**	__ __ __

**CRF Completed by (Initials) – to be signed when complete.** *Do not sign if any fields are empty*	__ __ __	Date<br>__ __/__ __/__ __ __ __<br>*D D / M M / Y Y Y Y*	Time<br>__ __: __ __

For peer review only

For peer review only - http://bmjopen.bmj.com/site/about/guidelines.xhtml

CHAIN Enrolment CRF v1.63
CHAIN Number [1][0] [0][0][3] [ ][ ][ ]

PART 2

CHAIN ADMISSION CRF: SOCIAL INFORMATION.

To be completed within 48h of admission when child is stable. This should ideally be done in a conversational and unhurried way, with the interviewer sitting with the caregiver.

Initials of person interviewing caregiver and completing part 2<br>___ ___ ___<br>☐ Doctor ☐ Clinical officer ☐ Nurse ☐ Field worker ☐ Research Assistant ☐ Other	Date<br>__ __/__ __/__ __ __ __<br>D D/M M/ Y Y Y Y<br>Time<br>___ ___:___ ___

Who is being interviewed?

☐ Primary caregiver only ☐ Care home staff ☐ Primary caregiver and one other person ☐ Primary caregiver and more than one other person ☐ One person who is not the primary caregiver ☐ More than one person who is not the primary caregiver

Care-seeking Behaviour

**Was the child in generally good health before this illness?**	☐ Y	☐ N	☐ Unknown
**If No, how long has the child had this problem of generally bad health?**	__ __ weeks	☐ N/A	
**Does the child have health insurance?**	☐ Y	☐ N	☐ Unknown

What was the main reason for bringing the child to this hospital today? *Reasons given, select one*

☐ Referred by health care worker ☐ Caregiver concern of child's condition ☐ Received money for transport to hospital (e.g. from family, neighbour, paid work)?
☐ Relative / neighbour concern of child's condition ☐ Primary caregiver returned home e.g. if working away ☐ Other

How did you travel to the hospital? *Select all that apply*

☐Car/ Taxi ☐ Ambulance ☐ Bus ☐Motorbike ☐ Tuk-tuk /CNG ☐ Cycle rickshaw ☐ Train ☐ Walking ☐ Other

How long did it take you to travel to hospital? ☐ <1h ☐ 1- < 2h ☐ 2-4h ☐ >4h ☐ > 1 day

How much did it cost the family to travel to hospital today (in local currency? *Estimate amount. If walked or free ambulance write 0* ___ ___ ___ ___

Have you sought treatment for this illness prior to coming to hospital? *Select all that apply*

☐ No treatment sought ☐ Shop ☐ Government hospital ☐ Government dispensary ☐ Traditional Healer
☐ Pharmacy ☐ Private Medical Facility/ NGO ☐ Herbalist ☐ Homeopathist ☐ Other

Received treatment from traditional healer, homeopathist or herbalist in last 4 weeks? Y N

Child's Health Status Before Admission

Before this illness, how did this child's health compare to other children of similar age in your neighbourhood? *Select one*

☐Similar ☐Better ☐Worse ☐Don't know

Before this illness, how did this child's health compare to his/her siblings at a similar age? *Select one*

☐Similar ☐Better ☐Worse ☐ Don't know ☐ N/A only child

For peer review only - http://bmjopen.bmj.com/site/about/guidelines.xhtml

CHAIN Enrolment CRF v1.63
CHAIN Number [1][0] [0][0][3] [ ][ ][ ]

Birth History					
**Source of information**	☐ Maternal/caregiver recall	☐ Book/medical records			
**Birth weight**	___ .___ ___kg	☐Unknown			
**Birth details** *Select any that apply*	☐ Premature	☐ Born small <2.5kg	☐Twin/multiple birth	☐ Born at term	☐ Unknown
**Delivery location** *Select one*	☐ Born in hospital	☐ Community facility/clinic with midwife/nurse midwife/doctor			
	☐ Home without birth attendant	☐ Home with traditional birth attendant (untrained)	☐ Home with midwife/nurse		
	☐ Other		☐ Unknown		
**Delivery details** *Select all that apply*	☐ Normal, spontaneous vaginal delivery	☐ Assisted delivery (forceps, ventouse)	☐ Caesarean section		
	☐ Admitted neonatal unit	☐ Mother admitted to hospital >48h	☐ Unknown		
**Mother's age at first pregnancy**	___ ___years ☐ unknown	**Mother's age now**	___ __years	☐ unknown	
**Participant birth order**	___ ___ of ___ ___ total live births (*e.g. if youngest of 3 children 3 of 3, if oldest of 3 children 1 of 3)*				
**Are the biological parents of this child consanguineous?** *Ask if parents have relatives in common or are related.*	☐ Yes	☐ No	☐ Unknown		

For peer review only

For peer review only - http://bmjopen.bmj.com/site/about/guidelines.xhtml

CHAIN Enrolment CRF v1.63

CHAIN Number [1][0] [0][0][3] [ ][ ][ ]

**Primary Caregiver Information**<br>***This is the person who has responsibility for day to day care of the child, but is not a substitute carer such as childminder or grandparent who cares for child whilst, for example, mother is at work.***			
**Who is the Primary Caregiver?** *Select one*	☐Biological Parent ☐Grandparent ☐Sibling ☐Aunt / Uncle / Cousin<br>☐ Stepmother / father ☐ Care home /orphanage ☐Other/ Unclear		
**Is the child's biological father alive?**	☐ Y ☐ N ☐ Unknown	**Is the child's biological mother alive?**	☐ Y ☐ N ☐ Unknown
**Primary Care Giver Age** *Select one*	☐ <18years ☐ >=18 years ☐ >50years ☐ N/A (care home or unclear)		
**Primary Care Giver Sex** *Select one*	☐ Male ☐ Female ☐ N/A	**Primary caregiver present at admission?**	☐ Y ☐ N
**Has the primary caregiver lived in the same household as the child for the last 2 months?**			☐ Y ☐ N ☐ N/A (care home)
**Marital status of primary caregiver** *Select one*	☐ Married/ monogamous ☐ Married polygamous ☐ Single ☐ Separated / divorced ☐ Widowed ☐ N/A		
**If not present at admission, where is the primary caregiver?** *Select one*			
☐ Home ☐ Work ☐ School ☐ Unknown ☐ Other_______ ☐ N/A			
**If the primary caregiver is present, caregiver anthropometry:**<br>*Use locally available adult scales and stadiometer, and adult MUAC tapes provided by CHAIN.*			
☐ **Primary caregiver not present during admission, or care home**			
**Weight** ___ ___. ___ kg	**MUAC** ___ ___ .____.cm	**Height:** ___ ___ ___cm	
**Education:** *Select highest level of education achieved*	☐ None ☐ Primary ☐ Secondary ☐ Above secondary ☐ Unknown ☐ N/A care home		
**Able to read?**	☐ Y ☐ N ☐Unknown	**Is the primary caregiver primarily responsible for financial support and providing for the child?**	☐ Y ☐ N
**Primary caregiver HIV status in last 6 months** *Select one*	☐ Tested Positive ☐ Tested Negative ☐ Not tested or unknown		

Have there been ANY changes to the child's social situation in the last 2 MONTHS? *Select any that apply,*

**Child moved to a different household**	**Y N**	**Relocation from rural to urban setting** *Select 'yes' even if this is temporary*	**Y N**
		**Relocation from urban to rural setting** *Select 'yes' even if this is temporary*	**Y N**
		**Relocation to live with different caregiver** *Select 'yes' even if this is temporary*	**Y N**
**Mother sick**	**Y N**	**Mother Died**	**Y N**
**Father sick**	**Y N**	**Father Died**	**Y N**
**Other primary caregiver sick**	**Y N N/A**	**Other primary caregiver died**	**Y N N/A**
**Primary caregiver changed**	**Y N**	**Child went into care home**	**Y N**
**Primary caregiver started employment / returned to school**	**Y N**	**Person providing for the child has lost income**	**Y N**
**Primary caregiver divorced / separated from partner**	**Y N**	**Primary caregiver in new relationship**	**Y N**
**Mother is pregnant**	**Y N**	**Mother gave birth**	**Y N**
**Other primary caregiver pregnant?**	**Y N N/A**	**Other primary caregiver gave birth**	**Y N N/A**

If primary caregiver has changed in the last 2 months, who was the child's previous primary caregiver? *Select one*

☐Biologic Mother ☐Biologic Father ☐Sibling ≥18 years old ☐Sibling <18 years old

☐Grandparent ☐Aunt/Uncle/Cousin ☐Other ☐ N/A

For peer review only - http://bmjopen.bmj.com/site/about/guidelines.xhtml

CHAIN Enrolment CRF v1.63

CHAIN Number [1][0] [0][0][3] [ ][ ][ ]

Primary caregiver earns an income now? *Ask the person accompanying the child and select one*

☐ Employed full time by someone else	☐ Employed part time by someone else
☐ Works for self	☐ No work income
☐ Works casually/irregularly for someone	☐ Don't know
If works casually, Occupation:	☐ N/A care home

**How many days worked a week?** *Select one*	☐ **<3**	☐ **3-5**	☐ **>5**	☐ N/A, does not work for income

If the primary caregiver earns, main source of income? *Select one*

☐ Farmer	☐ Business/trader	☐ Labourer	☐ Domestic work
☐ Other private sector employment	☐ Public sector employment	☐ Retired with pension income	
☐ Begging	☐ Other__________________	☐ N/A	

If the primary caregiver works (earning or non-earning), main place of work? *Select one*

☐In/around home (where child lives)	☐ Away for <4 hours per day	☐Away >4 hours but comes home daily
☐Away > 8h a day but returns home daily	☐Away >1 day, comes home weekly	☐ Away comes home, less than weekly
☐Primary caregiver lives and works away	☐ Don't know	☐ N/A

The person primarily providing financial support to this child is this child's: *Select one*

☐ Biologic Mother	☐ Biologic Father	☐ Stepfather	☐ Stepmother
☐ Grandparent	☐ Sibling ≥18 years old	☐ Sibling <18 years old	☐ Aunt/Uncle/Cousin
☐ More than one person responsible, unclear	☐ Unsupported / care home	☐ Other -specify________________	

Person responsible for providing financial support to child, place of usual residence? *Select one*

☐ Always sleeps at home	☐ Sleeps away but returns weekly
☐ Sleeps away for > two months per year	☐ Works and lives abroad, contact with child once a year or less
☐ Sleeps away but return monthly or less often	☐ Don't know
☐ Other____	☐ N/A (e.g. care home, unsupported)

What is the Father or person responsible for providing financial support to child source of income?
Select one. If the primary carer is also the person providing financial support do not complete this section.

☐ Farmer	☐ Business/trader	☐ Labourer	☐ Domestic work
☐ Other private sector employment	☐ Public sector employment	☐ Retired with pension income	
☐ Begging ☐None ☐ Unknown	☐ Other ____________________	☐ N/A	

For peer review only

For peer review only - http://bmjopen.bmj.com/site/about/guidelines.xhtml

CHAIN Enrolment CRF v1.63

CHAIN Number [1][0] [0][0][3] [ ][ ][ ]

**Substitute Care:**<br>*Who usually looks after child when primary caretaker is working or away? Select all that apply*					
☐ Not applicable, caregiver looks after child full time		☐ Not applicable, child accompanies caregiver to work			
☐ No substitute care, child left alone		☐ No substitute care / unclear		☐ Child in care home	
☐ Biological Mother	☐ Biological Father	☐ Sibling <18 years old		☐ Sibling ≥18 years old	
☐ Grandparent	☐ Aunt/Uncle/Cousin	☐ Childcare facility outside home		☐ Childminder/ day care at home	

**How many days a week is the child in day care?**	☐ N/A	☐ 1-2	☐ 3-4	☐ 5-6	☐ >6	
**How many hours per day is the child in day care?**	☐ N/A	☐ 1-4h	☐ 5-8h	☐ 9-12h	☐ >12h	
**How many children are looked after at this day care?**	☐ <3	☐ 4-6	☐ 7-10	☐ >10	☐Unknown	☐ N/A
**How many of these are under 2y?**	☐ <3	☐ 4-6	☐ 7-10	☐ >10	☐Unknown	☐ N/A
**How many adults look after these children?**	☐ 1	☐2-4	☐5-10	☐ >10	☐ N/A	
**Do you feel the day care is good?**	☐ Y	☐ N	☐ N/A			

**Who provides food for the child at day care?** *Select one*				
☐ Caregiver provides food for the child	☐ Day care provides food for the child	☐ Someone else provides food for the child	☐ Don't know	☐ N/A

**Is feeding supervised / assisted at day care?**	☐ Y	☐ N	☐ Unknown	☐ N/A

**Household Food Security**<br>(if child in care home include **children** in the care home only)			
**During the past 7 DAYS** has ANY member of the household missed a meal due to food shortage?	☐ Y	☐ N	☐ Unknown
**During the past 4 WEEKS**<br>Did you worry that your household would not have enough food?	☐ Y	☐ N	☐ Unknown
Were any of your household unable to eat the kinds of food preferred because of a lack of resources?	☐ Y	☐ N	☐ Unknown
Have any of your household had to eat a limited variety of food due to lack of resources?	☐ Y	☐ N	☐ Unknown
Have any of your household eaten some foods that you really didn't want to eat because of lack of resources?	☐ Y	☐ N	☐ Unknown
Have any of your household eaten fewer meals in a day because there was not enough food?	☐ Y	☐ N	☐ Unknown
Did household members go to sleep at night hungry because there was not enough food?	☐ Y	☐ N	☐ Unknown
Did you or your household members go a whole day and night without eating anything because there was not enough food?	☐ Y	☐ N	☐ Unknown

For peer review only - http://bmjopen.bmj.com/site/about/guidelines.xhtml

CHAIN Enrolment CRF v1.63
CHAIN Number [1][0] [0][0][3] [ ][ ][ ]

Child Dietary Diversity
What does the child eat on a typical day?<br>• *Ask this as an open question and select all that the caregiver mentions.*<br>• *Do not present the caregiver with this list.*<br>• *You may prompt the caregiver with open questions, e.g. What does your child usually eat for breakfast*
☐ **Milk and Milk Products**: Fresh/fermented milk, cheese, yogurt, or other milk products
☐ **Breast milk**
☐ **Cereals and Cereal Products**: Maize, rice, pasta, porridge, bread, biscuits, millet, sorghum, wheat, locally available grains
☐ **Fish and Sea Foods**: fresh or dried fish or shellfish
☐ **Roots and Tubers**: potatoes, sweet potatoes, yams, cassava, or foods made from roots or wild roots and tubers
☐ **Vegetables**: Cabbages, carrots, spinach, and any other locally available vegetables including wild vegetables
☐ **Fruits**: Oranges, bananas, mangoes, avocados, apples, grapes etc
☐ **Meats and Poultry**: Camel, beef, lamb, goat, rabbit, wild game, chicken or other birds, liver, kidney, heart or other organ meats or blood-based foods
☐ **Eggs**: Hen or other bird eggs
☐ **Pulses / Legumes / Nuts and Seeds**: Beans, peas, lentils, nuts, seeds or foods made from these
☐ **Fats and Oils**: Oil, fats, ghee, margarine or butter added to food or used for cooking
☐ **Sugars / Honey and Commercial Juices**: Sugar in tea, honey, sweetened soda, juices, chocolates, sweets or candies
☐ **Miscellaneous**: Spices, unsweetened beverages
☐ **UNKNOWN**

Feeding practices	
**How is food USUALLY given to the child? *Select one***	
☐ Fed by adult	☐ Child feeds self, unsupervised
☐ Child feeds self, supervised by adult	☐ Fed from common plate or bowl
☐ Child feeds self, supervised by older children	☐ Child exclusively breastfed
☐ Unknown	☐ Other

For peer review only - http://bmjopen.bmj.com/site/about/guidelines.xhtml

CHAIN Enrolment CRF v1.63

CHAIN Number [1][0] [0][0][3] [ ][ ][ ]

Assessment of household wealth (DHS 7 questionnaire. Please answer all questions, for all participants, including children in care homes)		
**What is the main source of drinking water for members of your household?** *Choose one*		
☐ Piped water to dwelling	☐ Cart with small tank	☐ from vendor
☐ Piped water to yard / plot	☐ Tanker truck	☐ Rainwater
☐ Piped to neighbour	☐ Bottled water	☐ Stream/river/lake/pond/dam
☐ Public tap/ Standpipe	☐ Protected spring	☐ Unknown
☐ Protected well / borehole	☐ Unprotected spring	
☐ Unprotected well	☐ Other	
**What is the MAIN source of water used by your household for other purposes such as cooking and handwashing? SELECT ONE ONLY**		
☐ Piped water to dwelling	☐ Cart with small tank	☐ Bought from vendor
☐ Piped water to yard / plot	☐ Tanker truck	☐ Rainwater
☐ Piped to neighbour	☐ Bottled water	☐ Stream/river/lake/pond/dam
☐ Public tap/ Standpipe	☐ Protected spring	☐ Unknown
☐ Protected well / borehole	☐ Unprotected spring	
☐ Unprotected well	☐ Other	

**How long does it take to get DRINKING water and come back? (State 0 if water supplied within home or compound)**	__ __ __minutes		☐ Don't know
**In the past 2 weeks was the water from this source not available for at least one full day?**	☐ Y	☐ N	☐ Unknown

**Do you usually do anything to the water to make it safer to drink?** *Select all that apply*			
☐ None	☐ Bleach/ chlorine	☐ Strain through a cloth	☐ Let it stand and settle
☐ Use water filter (ceramic/sand/composite etc)	☐ Solar disinfection	☐ Boil	☐ Other

For peer review only

For peer review only - http://bmjopen.bmj.com/site/about/guidelines.xhtml

CHAIN Enrolment CRF v1.63
CHAIN Number [1][0] [0][0][3] [ ][ ][ ]

**What kind of toilet facility do members of your household usually use?** *Select one*		
☐ Flush or pour flush toilet to piped sewer	☐ Flush to septic tank	☐ Ventilated improved pit latrine
☐ Flush to pit latrine	☐ Flush to somewhere else	☐ Open pit / Pit latrine without slab
☐ Flush don't know where	☐ Composting toilet	☐ Bucket toilet
☐ Pit latrine with slab	☐ Hanging toilet / hanging latrine	☐ No facility / bush/ field
☐ Unknown		

**Do you share this toilet facility with other households?**	☐ Y	☐ N	☐ Unknown	
**If Yes, including your own household, how many households use this toilet facility?**	Number if <10__	☐ >10 households	☐ Unknown	☐ N/A
**Where is this toilet facility located?**	☐ In own dwelling	☐ In own yard / plot	☐ Elsewhere	
**How many rooms are there in the household for SLEEPING?**	☐ **1**	☐ **2**	☐ **>2**	

**What is the MAIN FLOOR material of the rooms in this household?** *Select one only*		
☐ Cement	☐ Earth/sand	☐ Wood
☐ Dung	☐ Lives on boat	☐ Tiles
☐ Carpet	☐ Other (specify)____________________	☐ Unknown

**What is the MAIN WALL material of the rooms in this household?** *Select one only*			
☐ Grass/straw/makuti	☐ Stone	☐ Wood	☐ Unknown
☐ Corrugated iron sheet/ Tin	☐ Mud/wood	☐ Brick/block	
☐ Planks/shingles	☐ No wall	☐ Other (specify) __________________	

**What is the MAIN ROOF material of the house in this household?** *Select one only*		
☐ Grass/Thatch	☐ Tiles/Asbestos sheets	☐ Corrugated iron/ Tins
☐ Mud	☐ Nylon papers/clothes	☐ Concrete
☐ Other (specify) __________________________		☐ Unknown

**What is the MAIN cooking fuel used in this household?** *Select one only*		
☐ Electricity	☐ LPG /Natural gas/Biogas	☐ Paraffin
☐ Coal / Lignite	☐ Charcoal	☐ Firewood
☐ Straw/shrubs/grass	☐ Agricultural crop	☐ Animal Dung
☐ No food cooked in household	☐ Other (specify)_____________________	☐ Unknown

**Do you have a separate room which is used as a kitchen?**	☐ Y	☐ N	☐ Unknown

**Where is this household's cooking area located?**				
☐ In the house	☐ Outdoors	☐ In a separate building	☐ Other ______________	☐ Unknown

For peer review only - http://bmjopen.bmj.com/site/about/guidelines.xhtml

CHAIN Enrolment CRF v1.63

CHAIN Number [1][0] [0][0][3] [ ][ ][ ]

**Does this household own any livestock, herds, other farm animals or poultry**	☐ Y	☐ N	☐ Unknown
**If yes, how many of the following animals does this household own?**			
Cows/bulls__ __ Sheep__ __<br>Horses/Donkeys/Mules__ __ Goats__ __<br>Chickens or Ducks__ __ Other _____________ number ___ ___			☐ N/A
**Does any member of this this household own land?**	☐ Y	☐ N	☐ Unknown
**If "Yes" How many acres of land does this household own?**	_____Acres	☐ Unknown	☐ N/A
**Does this household have a bank account?**	☐ Y	☐ N	☐ Unknown
**Does this household have electricity**	☐ Y	☐ N	☐ Unknown
**Does this household own a radio?**	☐ Y	☐ N	☐ Unknown
**Does this household own a television?**	☐ Y	☐ N	☐ Unknown
**Does this household own a computer?**	☐ Y	☐ N	☐ Unknown
**Does this household own a refrigerator?**	☐ Y	☐ N	☐ Unknown
**Does any member of this household own:**			
**A watch**	☐ Y	☐ N	☐ Unknown
**A mobile phone?**	☐ Y Standard phone / ☐ Y smartphone	☐ N	☐ Unknown
**An animal-drawn cart?**	☐ Y	☐ N	☐ Unknown
**A bicycle?**	☐ Y	☐ N	☐ Unknown
**A motorcycle / scooter?**	☐ Y	☐ N	☐ Unknown
**A car or truck?**	☐ Y	☐ N	☐ Unknown
**A boat with a motor?**	☐ Y	☐ N	☐ Unknown

**CRF Completed by (Initials) – to be signed when complete.**<br>*Do not sign if any fields are empty*	__ __ __	Date<br>__ __/ __ __/ __ __ __ __<br>*D D / M M / Y Y Y Y*	Time<br>__ __: __ __

END

For peer review only - http://bmjopen.bmj.com/site/about/guidelines.xhtml

STROBE (Strengthening The Reporting of OBservational Studies in Epidemiology) Checklist

A checklist of items that should be included in reports of observational studies. You must report the page number in your manuscript where you consider each of the items listed in this checklist. If you have not included this information, either revise your manuscript accordingly before submitting or note N/A.

Note: An Explanation and Elaboration article discusses each checklist item and gives methodological background and published examples of transparent reporting. The STROBE checklist is best used in conjunction with this article (freely available on the Web sites of PLoS Medicine at http://www.plosmedicine.org/, Annals of Internal Medicine at http://www.annals.org/, and Epidemiology at http://www.epidem.com/). Information on the STROBE Initiative is available at www.strobe-statement.org.

Section and Item	Item No.	Recommendation	Reported on Page No.
**Title and Abstract**	1	(*a*) Indicate the study's design with a commonly used term in the title or the abstract	1
		(*b*) Provide in the abstract an informative and balanced summary of what was done and what was found	1
**Introduction**			
Background/Rationale	2	Explain the scientific background and rationale for the investigation being reported	
Objectives	3	State specific objectives, including any prespecified hypotheses	
**Methods**			
Study Design	4	Present key elements of study design early in the paper	4
Setting	5	Describe the setting, locations, and relevant dates, including periods of recruitment, exposure, follow-up, and data collection	5
Participants	6	(*a*) *Cohort study*—Give the eligibility criteria, and the sources and methods of selection of participants. Describe methods of follow-up	4-6
		Case-control study—Give the eligibility criteria, and the sources and methods of case ascertainment and control selection. Give the rationale for the choice of cases and controls	NA
		Cross-sectional study—Give the eligibility criteria, and the sources and methods of selection of participants	NA
		(*b*) *Cohort study*—For matched studies, give matching criteria and number of exposed and unexposed	NA
		Case-control study—For matched studies, give matching criteria and the number of controls per case	NA
Variables	7	Clearly define all outcomes, exposures, predictors, potential confounders, and effect modifiers. Give diagnostic criteria, if applicable	6-8

For peer review only

Section and Item	Item No.	Recommendation	Reported on Page No.
Data Sources/ Measurement	8*	For each variable of interest, give sources of data and details of methods of assessment (measurement). Describe comparability of assessment methods if there is more than one group	6-9
Bias	9	Describe any efforts to address potential sources of bias	NA
Study Size	10	Explain how the study size was arrived at	11
Quantitative Variables	11	Explain how quantitative variables were handled in the analyses. If applicable, describe which groupings were chosen and why	10-11
Statistical Methods	12	(*a*) Describe all statistical methods, including those used to control for confounding	10-11
		(*b*) Describe any methods used to examine subgroups and interactions	10-11
		(*c*) Explain how missing data were addressed	NA
		(*d*) *Cohort study*—If applicable, explain how loss to follow-up was addressed	10-11
		Case-control study—If applicable, explain how matching of cases and controls was addressed	NA
		Cross-sectional study—If applicable, describe analytical methods taking account of sampling strategy	NA
		(*e*) Describe any sensitivity analyses	NA
**Results**			
Participants	13*	(a) Report numbers of individuals at each stage of study—eg numbers potentially eligible, examined for eligibility, confirmed eligible, included in the study, completing follow-up, and analysed	NA
		(b) Give reasons for non-participation at each stage	NA
		(c) Consider use of a flow diagram	NA
Descriptive Data	14*	(a) Give characteristics of study participants (eg demographic, clinical, social) and information on exposures and potential confounders	NA
		(b) Indicate number of participants with missing data for each variable of interest	NA
		(c) *Cohort study*—Summarise follow-up time (eg, average and total amount)	NA
Outcome Data	15*	*Cohort study*—Report numbers of outcome events or summary measures over time	NA
		Case-control study—Report numbers in each exposure category, or summary measures of exposure	NA
		Cross-sectional study—Report numbers of outcome events or summary measures	NA

For peer review only

Section and Item	Item No.	Recommendation	Reported on Page No.
Main Results	16	(*a*) Give unadjusted estimates and, if applicable, confounder-adjusted estimates and their precision (eg, 95% confidence interval). Make clear which confounders were adjusted for and why they were included	NA
		(*b*) Report category boundaries when continuous variables were categorized	NA
		(*c*) If relevant, consider translating estimates of relative risk into absolute risk for a meaningful time period	NA
Other Analyses	17	Report other analyses done—eg analyses of subgroups and interactions, and sensitivity analyses	NA
**Discussion**			
Key Results	18	Summarise key results with reference to study objectives	NA
Limitations	19	Discuss limitations of the study, taking into account sources of potential bias or imprecision. Discuss both direction and magnitude of any potential bias	NA
Interpretation	20	Give a cautious overall interpretation of results considering objectives, limitations, multiplicity of analyses, results from similar studies, and other relevant evidence	NA
Generalisability	21	Discuss the generalisability (external validity) of the study results	NA
**Other Information**			
Funding	22	Give the source of funding and the role of the funders for the present study and, if applicable, for the original study on which the present article is based	13

*Give information separately for cases and controls in case-control studies and, if applicable, for exposed and unexposed groups in cohort and cross-sectional studies.

Once you have completed this checklist, please save a copy and upload it as part of your submission. DO NOT include this checklist as part of the main manuscript document. It must be uploaded as a separate file.

For peer review only

BMJ Open

The Childhood Acute Illness and Nutrition (CHAIN) Network: Protocol for A Multi-site Prospective Cohort Study to Identify Modifiable Risk Factors for Mortality Among Acutely Ill Children in Africa and Asia.

Journal:	*BMJ Open*
Manuscript ID	bmjopen-2018-028454.R2
Article Type:	Protocol
Date Submitted by the Author:	11-Mar-2019
Complete List of Authors:	The Childhood Acute Illness and Nutrition (CHAIN), Network; University of Washington System, Global Health<br>Walson, Judd; University of Washington, Department of Global Health
**Primary Subject Heading**:	Paediatrics
Secondary Subject Heading:	Global health, Epidemiology, Evidence based practice, Nutrition and metabolism, Public health
Keywords:	Acute illness, hospitalisation, post-discharge mortality, malnutrition

SCHOLARONE™
Manuscripts

**Title:**	The Childhood Acute Illness and Nutrition (CHAIN) Network: Protocol for A Multi-site Prospective Cohort Study to Identify Modifiable Risk Factors for Mortality Among Acutely Ill Children in Africa and Asia.
**Running head:**	CHAIN Cohort Study
**Authors:**	The Childhood Acute Illness and Nutrition (CHAIN) Network<br>* (authors listed in appendix as collaborators)
**Corresponding author (s)**	Judd Walson; walson@uw.edu ; +1 206 7443695<br>James A Berkley; JBerkley@kemri-wellcome.org; +254 720867011
**Affiliations:**	Centre for Tropical Medicine & Global Health, The University of Oxford, Oxford, UK; The Department of Global Health, The University of Washington, Seattle, USA; KEMRI-Wellcome Trust Research Programme, Kilifi, Kenya; The Childhood Acute Illness & Nutrition (CHAIN) Network
**Funded by:**	The Bill and Melinda Gates Foundation [OPP1131320]
**Trial registration**	Clinicaltrials.gov, NCT03208725
**Key words:**	Acute illness; malnutrition; post-discharge mortality; hospitalisation
**Word count:**	~4000

For peer review only

For peer review only - http://bmjopen.bmj.com/site/about/guidelines.xhtml

ABSTRACT

Introduction:

Children admitted to hospital in resource-poor settings remain at risk of both inpatient and post-discharge mortality. While known risk factors such as young age and nutritional status can identify children at risk, these factors do not provide clear mechanistic targets for intervention. The Childhood Acute Illness and Nutrition (CHAIN) Cohort Study aims to characterise biomedical and social risk factors for mortality in acutely ill children in hospital and after discharge to identify targeted interventions to reduce mortality.

Methods and analysis:

The CHAIN Network is undertaking a multisite, prospective, observational cohort enrolling children aged one week to two years at admission to hospitals at nine sites located in four African and two South Asian countries. CHAIN supports sites to provide care according to national and international guidelines. Enrolment is stratified by anthropometric status and children are followed throughout hospitalisation and for six months after discharge. Detailed clinical, demographic, anthropometric, laboratory and social exposures are assessed. Scheduled visits are conducted at 45, 90 and 180 days after discharge. Blood, stool and rectal swabs are collected at enrolment, hospital discharge, and follow-up. The primary outcome is inpatient or post-discharge death. Secondary outcomes include readmission to hospital and nutritional status after discharge. Cohort analysis will identify modifiable risks, children with distinct phenotypes, relationships between factors and mechanisms underlying poor outcomes that may be targets for intervention. A nested case-control study examining infectious, immunologic, metabolic, nutritional and other biological factors will be undertaken.

Ethics and dissemination:

This study protocol was reviewed and approved primarily by the Oxford Tropical Research Ethics Committee, and the institutional review boards of all partner sites. The study is being externally monitored. Results will be published in open access peer-reviewed scientific journals and presented to academic and policy stakeholders.

Trial Registration Number: NCT03208725.

Key words: Acute illness; malnutrition; post-discharge; mortality; hospitalisation; resource-poor; child survival; children; infants

For peer review only

STRENGTHS AND LIMITATIONS

- The CHAIN cohort is a multi-country study collecting comprehensive data on clinical, laboratory, social, economic and behavioural exposures at multiple sites in Africa and Asia.
- Heterogeneity across sites (geography, rural/urban, varying HIV and malaria prevalence etc). increases generalisability and may help identify context-independent and dependent interventions.
- Strict harmonisation of procedures, processes and tools allows for comparability between sites.
- This study is deliberately focused on hospitalised children and is not designed to evaluate factors associated with pre-hospital care and timing of presentation to hospital.
- Bias may be introduced by loss-to-follow-up after hospital discharge if this occurs in more vulnerable children, or if participation in the study alters outcomes.

For peer review only

For peer review only - http://bmjopen.bmj.com/site/about/guidelines.xhtml

INTRODUCTION

Despite impressive global reductions in child mortality, more than five million children under age five die each year.(1) These deaths occur disproportionately in low and middle-income countries (LMICs).(2) Factors underlying mortality risk are complex and overlapping, including undernutrition, acute and chronic co-morbidities, lack of access to care, poor social support systems and poverty.(3, 4) For many vulnerable children, these factors contribute to an unstable health trajectory, with repeated episodes of illness interspersed with short periods of incomplete recovery (Figure 1). In most areas of the world, acutely ill children may not access health care during episodes of illness.(5) Of those that do, studies have shown that children who are hospitalised have dramatically higher risk of death both during hospitalisation and in the months following discharge than their community peers, even when guidelines for treatment and clinical follow up are followed.(6-9) Thus, contact with the health system resulting in hospitalisation serves as an indicator of vulnerability and a time-point where children are readily accessible for intervention.(10-13) While many interventions may be available in hospitals, there are no clear interventions proven to reduce post-discharge mortality. Undernutrition, HIV infection young age, and a history of repeated hospitalisation have emerged as important risk factors for post-discharge death.(3, 9, 14)

Figure 1: A model of unstable health trajectory of a child characterised by repeated illness and incomplete recovery

For peer review only

For peer review only - http://bmjopen.bmj.com/site/about/guidelines.xhtml

Approximately half of all childhood mortality is associated with one or more forms of undernutrition, typically identified using simple anthropometry.(2) Mortality rates among children with severe acute malnutrition (SAM) are markedly elevated compared to the general population,(15) but with effective treatment in the community without infectious complications, case fatality may be as low as 1-2%.(16, 17) However, when children with SAM are acutely ill, inpatient case fatality is often 15-20% in resource-poor countries, with many additional deaths occurring after discharge, even among children who appear to be recovering anthropometrically.(18) Although case fatality rates for children with moderate acute malnutrition (MAM) or stunting in hospital and after discharge have not been widely reported, MAM and stunting are more prevalent than SAM and may contribute to a greater number of child deaths.(2, 19)

Current guidelines for the clinical management of acutely ill children with SAM, MAM or stunting are not supported by strong evidence.(20-23) In addition, while measures of mid-upper arm circumference, weight-for-length and length-for-age can identify children as malnourished, anthropometry does not identify the mechanisms that link undernutrition to mortality nor its underlying causes. In typical settings outside humanitarian emergencies, it is unclear to what extent malnutrition is driven by factors other than food intake, including recurrent or chronic infection, intestinal or systemic inflammation, malabsorption, disability, social vulnerability, parental ill-health, maternal education and agency, lack of financial resources, poor social support and limited access to health care.(24) Identifying markers of vulnerability and the underlying mechanisms that drive mortality may offer opportunity to target interventions to improve outcomes in many low-resource settings. In addition, understanding relationships between biological and social risk factors may allow optimisation of intervention packages within specific groups to achieve the largest mortality reductions.

The Childhood Acute Illness and Nutrition Network (CHAIN) is a collaborative group of investigators working across nine sites in four African and two South Asian countries committed to improving child survival and optimising growth and development outcomes in low-resource settings. CHAIN is conducting a prospective cohort study with the objective of determining phenotypes of groups of children at highest risk, defining the mechanisms underlying such risk and identifying modifiable targets for intervention.

METHODS

Objectives

The CHAIN Network is conducting a prospective cohort study of approximately 4500 children aged one week to two years of age presenting to health care facilities with acute illness deemed severe enough to require admission to hospital at nine sites in six low- or middle-income countries. Children are followed throughout hospitalisation, discharge and for six months post-discharge. The general objective of the study is to characterise acutely ill children and their outcomes in hospital and after discharge in order to identify modifiable pathways leading to death. Using these data, the CHAIN network will prioritise interventions based on deliverability and potential impact.

Specific Objectives

1) To determine factors associated with i) mortality in hospital; ii) mortality after discharge; iii) readmission to hospital; and iv) poor nutritional recovery.

For peer review only - http://bmjopen.bmj.com/site/about/guidelines.xhtml

2) To determine differences in clinical, social, and pathophysiologic (e.g., metabolic, infectious, and immune) phenotypes between children of different nutritional status at admission, discharge and follow-up.
3) To identify modifiable risk factors and potential interventions to reduce mortality amongst vulnerable children.

The goal of the CHAIN Network is to identify interventions that can reduce child mortality beyond the current recommendations for standard of care. Hence, prior to participant enrolment, all study sites underwent a formal assessment of their capacity and adherence to national and international guidelines, including a survey of human resources, materials, equipment, pharmaceutical stock and laboratory capacity. All sites were provided with support to ensure that the minimum standard of care achieved was in line with national and international care guidelines. All sites participate in an audit and feedback cycle of guideline adherence using the individual patient data collected by CHAIN.

Locations

Participating sites include rural and urban hospitals in Bangladesh (icddr,b Dhaka Hospital and Matlab Hospital), Burkina Faso (Banfora Regional Referral Hospital), Kenya (Kilifi County Hospital, Mbagathi sub-County Hospital and Migori County Referral Hospital), Malawi (Queen Elizabeth Central Hospital), Pakistan (Civil Hospital, Karachi), and Uganda (Mulago Hospital).

Patient and Public Involvement

The investigators who form the CHAIN Network leadership have extensive experience in the design and conduct of trials focused on improving growth and survival in children in Africa and Asia. The study was designed specifically to reflect patient priorities (child survival, reductions in hospitalization, improved growth. Working closely with Community Advisory Boards at each site, as well as with a robust social science team of researchers in Kenya and Bangladesh, patient priorities, experience and preferences were carefully considered in the design and development of the protocol and data collection instruments. The research questions were also discussed with a number of internal CHAIN Network and external experts in child survival at a convening organized by the funder. Caregivers were included in the Community Advisory Boards to provide input and into the conduct of the study. At the conclusion of the study, results will be disseminated to participants via seminars and outreach events led by the site PIs and the Community Advisory Boards at each site.

Eligibility

Children aged seven days to 23 months who are admitted to hospital with an acute illness are eligible for enrolment. Prior to October 2018, the lower age limit of enrolment was 60 days.

Exclusion criteria are;

- Unable to tolerate oral feeds in the 48 hours prior to the onset of the current acute illness
- Underlying terminal illness that in the opinion of the treating physician is likely to lead to death within 6 months
- Diagnosed with a condition that in the opinion of the treating physician is likely to require surgery within 6 months
- Diagnosed chromosomal abnormality (syndromically or genetically diagnosed abnormality)
- Primary reason for admission is trauma or surgery

For peer review only - http://bmjopen.bmj.com/site/about/guidelines.xhtml

- Requiring immediate resuscitation at admission defined by on-going cardiac or pulmonary arrest or judged to be peri-arrest by the attending physician
- Previous enrolment in the study
- Sibling enrolled in the study
- Caregiver plans to move outside of the hospital catchment area within six months
- Caregiver is unwilling to attend study visits
- Lack of informed consent

Screening

Each site has nominated a day on which weekly recruitment begins. The first eligible child in each enrolment group admitted to the hospital is identified and approached for consent. This is repeated for every eligible child admitted subsequently until weekly targets are met for each enrolment stratum (see Table 1 below). Although this is a pseudo-random approach, all the procedures aim to ensure that the study population adequately represents the spectrum of nutritional states observed over the recruitment period.

Potential participants are identified by clinical staff and brought to the attention of the study team who verify eligibility. If eligible, the study is explained to caregivers in their primary language and written informed consent to participate is sought. Caregivers who are unable to write are asked to provide a witnessed thumbprint. If consent is obtained, the child is enrolled and given a unique study number is allocated. If a child is deemed eligible but is too sick for consent to be immediately sought, study staff obtain verbal assent to collect both research and clinical samples at that time to avoid multiple needle insertions. If a caregiver who gave assent then chooses not to provide full written consent, all research data and samples are destroyed. Children classified as orphans or those living in alternative care homes are eligible for enrolment if an appropriate caregiver is present to provide consent on the child's behalf are eligible for enrolment.

Enrolment

Enrolment is stratified by nutritional status, aiming at a ratio of 2:2:1 (A: B: C) with group targets of 200 group A: 200 group B: 100 group C per site (Table 1).

Table 1: Enrolment groups by age and nutritional status

Group	Target N	Age >6 months	Age 1 - 6 months	Age < 1 month
A	1800	MUAC <11.5cm or kwashiorkor	MUAC <11cm or kwashiorkor	MUAC <9.5cm or kwashiorkor
B	1800	MUAC 11.5 to <12.5cm	MUAC 11 to <12cm	MUAC 9.5-10.4cm
C	900	MUAC ≧12.5cm	MUAC ≧12cm	MUAC ≧10.5cm

Mid-upper arm circumference (MUAC) was chosen as the optimal measure for participant selection as it is strongly associated with mortality, captures stunted children, varies less with dehydration than weight-based indices and is easily measured in sick children. (25, 26)

Procedures

For peer review only - http://bmjopen.bmj.com/site/about/guidelines.xhtml

All sites completed standardised training on variable definitions, identification of clinical signs, measurement of anthropometry, case report form (CRF) completion and data entry prior to starting the study, and training is repeated regularly. An independent study monitor (WESTAT) was hired to conduct site assessments to ensure harmonisation of study procedures across and between sites, as well as to ensure compliance with regulatory standards. Results from WESTAT's monitoring visits are for internal purposes and will be made available to the principal investigators at each site, the study funder and the CHAIN Network leadership and coordination teams.

Upon satisfaction of the inclusion criteria and completion of informed consent forms, a unique study ID is allocated. Baseline data, including demographic and social information, a detailed clinical examination and measurement of vital signs, including pulse oximetry, are collected using the standardised CRF. Anthropometry is performed (head circumference, MUAC, weight, and length). At admission, biological samples, including blood (up to 5mL for research purposes), rectal swabs and faecal samples are obtained. All children are offered provider-initiated counselling and testing for HIV, and a malaria rapid diagnostic test is done. Results of investigations performed for clinical care (complete blood count (CBC), biochemistry, glucose or any other laboratory investigations collected) are abstracted and recorded for study purposes. After treatment is initiated, data on the child's diet, social circumstances and if the mother is present, maternal mental health screening is undertaken. Other data on maternal characteristics collected include maternal MUAC, height, weight and demographic data. Additional maternal variables are listed in the study's enrolment CRF which is included as a supplementary file.

During admission, hospitalised children are reviewed daily and specific clinical features indicating illness progression and treatments are recorded on a structured daily CRF that is entered into the CHAIN database. In the event of death in hospital, a standard mortality audit questionnaire is completed by a designated member of the study team.

At discharge, the same clinical assessment as at admission, including anthropometry, is conducted and blood and faecal sampling are repeated.

Follow-up procedures

A home visit is conducted within three days of discharge. The location of the household is recorded by Global Positioning System (GPS) and CRFs are used to capture information on the number of people living in the homestead, access to clean water and improved sanitation, occupation, household assets, income and food security. Parents and legal guardians are also interviewed about their home and social situation, including challenges experienced when keeping their child and family healthy.

Children are followed-up again at 45, 90 and 180 days after discharge at the study facilities, irrespective of scheduled or unscheduled outpatient visits for medical or nutritional care. A standardised questionnaire ascertaining vital status, care-seeking and re-hospitalisation history and recent dietary intake (up to seven days before contact with the hospital) is collected and anthropometry, rectal swabs, faecal specimens and blood samples are repeated at each follow-up visit. Maternal mental health is screened again at day 45.

Children judged to have significant illness at any follow up visit or at the home visit are referred to an appropriate hospital, clinic or nutrition programme. The study staff share any test result relevant to the patient's care with the clinical management team and families.

Parents and legal guardians are asked to bring their child to the study clinic if they are concerned that the child is unwell. Financial reimbursement for transport and lost earnings at standard local

For peer review only - http://bmjopen.bmj.com/site/about/guidelines.xhtml

rates is provided at the clinic visit. Study participants who are re-admitted to study hospitals undergo the standard clinical assessment delivered at enrolment. Participants who are admitted to hospitals other than the study site hospital have medical data abstracted onto standardised hospital re-admission forms. For deaths occurring outside the hospital, a verbal autopsy to evaluate the cause of death is completed within 28 days of study staff becoming aware of a death, using select questions from the WHO standard verbal autopsy tool.(27)

Community participants

To establish community norms, 125 children at each site living in the same community as hospitalised participants are recruited as community participants based on the following inclusion criteria: absence of known untreated HIV or TB; no hospital admission in the 14 days prior to contact with the study team; and no previously participation in the study. The exclusion criteria listed for hospitalised children also apply to community participants.

At every site, one in four hospital participants has a child enrolled from their community. Selection of hospitalised participants is either every fourth participant, or, in periods when enrolment in hospital was lower, for example during health care workers strikes, one community participant was retrospectively enrolled for every second hospitalised participant. Community participants are identified randomly from the hospitalised participants home; a random number x (1-4) and direction (north, south, east, west) are generated using an online tool prior to visiting the home. Random number selection was done using a web-based application, Random Number Generator/ Picker.(28) Once at the home of the hospitalised participant the research team begin by visiting the xth house in the generated direction and attempt to obtain consent to enrol a child within the eligible age range from that household. If not successful they continue in the same direction to the next x^{th} house. This is repeated until a child is enrolled. Children with severe illness requiring hospital admission identified during community screening are referred for appropriate care.

When an eligible community participant is identified, their carer is given information about the study and invited to the study clinic for assessment. Following confirmation of informed consent, a clinical examination and anthropometry are completed and documented, and blood and stool samples collected as in the hospitalised children. Household and demographic questionnaires are also administered. Children in this group requiring non-urgent medical care receive basic treatment in the study clinic and/or are referred to appropriate treatment centres after enrolment procedures are completed. These children remain eligible for inclusion as community participants. Financial reimbursement for transport and lost earnings at standard local rates is provided to each carer at the clinic visit. No follow-up is done on community participants.

Specimen collection

To ensure comparability, standard operating procedures (SOPs) are followed at each site. Blood samples are collected at enrolment, each day of the hospitalisation, in the event of clinical deterioration (defined by the onset of a new Integrated Management of Childhood Illness (IMCI) danger sign), at discharge, at all follow-up points, and at any hospital readmission. Blood is collected into a BD Vacutainer Hemogard™ K_2EDTA (spray dried) and a red top with clot activator (spray dried)) at each time point and three dried blood spots on Whatman filter paper cards are prepared. At each time point, a complete blood count and clinical biochemistry, including sodium, potassium, calcium, magnesium, urea, creatinine, albumin, bilirubin, alanine aminotransferase, inorganic phosphate and alkaline phosphate are performed. Blood glucose, HIV testing (Alere Determine or Uni-Gold HIV) and malaria rapid diagnostics tests (CareStart HRP2/pLDH) are also performed on all children at enrolment and at additional time points if clinically indicated. Caregivers

For peer review only - http://bmjopen.bmj.com/site/about/guidelines.xhtml

may refuse any sample collection during the study without being excluded from further follow-up. The schedule for blood collection is detailed in Table 2.

Stool collected directly or from child diapers (if fresh) is transferred into standardised stool collection pots by study staff, aliquoted and stored at -80˚C. Advanced pathogen detection using Taqman Array Card, sequencing, microbiome analysis, metabolomics and markers of enteric inflammation and dysfunction will be conducted on a subset of stool samples in the CHAIN nested case control study.

For peer review only

Table 2: Schedule of blood samples and tests performed at each collection time point

		ADMISSION	DISCHARGE	DAY 45	DAY 90	DAY 180	RE-ADMISSION
**Results used for care**	CBC	X	X	X	X	X	X
	Biochemistry	X					X
	Glucose	X					X
	Blood gas + lactate*	X					X
	Blood culture*	X					X
	Malaria RDT	X					X
	HIV test	X					
**Processed or stored for research assays only**	Dried blood Spot	X	X	X	X	X	X
	Serum + plasma for storage	X	X	X	X	X	X
	Whole blood for storage	X	X	X	X	X	X
	Functional immunology*	X	X	X	X	X	X
	PBMC extraction*	X	X	X	X	X	X
	Rectal swab & whole stool	X	X	X	X	X	X

* done at a subset of sites with capacity, thus these are sub-studies.

For peer review only - http://bmjopen.bmj.com/site/about/guidelines.xhtml

Data Management and Confidentiality

Data recorded on standardised CRFs are de-identified and entered in a secured central database and housed on servers in Nairobi with secure offsite backup. Prior to and after data entry, paper records are kept in a locked room with locked filing cabinet at each site, with access limited to investigators and study staff directly involved in data collection and entry. For de-identification, only participant initials are recorded and any potential identifier such as date of birth and GPS location are stored separately. Site principal investigators or their delegates conduct regular internal quality assurance on completion, data transfer and storage of the CRFs. A central data management system generates automated queries and review incoming data weekly. Any missing or implausible data is queried and site teams are given specific timeframe to resolve raised issues.

Analysis

Data from all Network sites will be combined as a single cohort. Outcomes will be classified as one or more of the following, the proportion of children with each endpoint described with 95% confidence intervals:

- Death at any time during the period of observation (primary endpoint)
- Death in hospital
- Death after discharge
- Readmitted to hospital within 180 days
- Nutritional status at 180 days
- Recovery as defined by survival without readmission or development of severe acute malnutrition during 180 days after discharge.

Primary cohort analysis - mortality

The hazard of mortality during the study will be calculated in multivariable survival models including i) clinical signs and symptoms; ii) anthropometric markers of wasting and stunting; iii) markers of organ function; iv) birth history and prior health; and v) maternal, household and social factors. The stratified design by location and nutritional category will be accounted for with multilevel fixed and random effects. Sub-group analyses will be conducted for inpatient and post-discharge mortality, including time-to-event survival analyses. Predictive algorithms will be built using methods such as classification and regression trees (CART), boosted regression trees and other machine learning methods. Mechanistic pathways will be interrogated using structured equation modelling, latent class analysis and other methods.

Secondary cohort analysis

Secondary endpoints are re-hospitalisation and the presence of SAM or MAM after 6 months. These will be examined using generalised linear models accounting for the competing risk of mortality. Growth trajectories post discharge will be compared to WHO standard growth curves and modelled in relation to risk factors and outcomes as panel data. Differences in mortality between sites will be examined in relation to case-mix and underlying risk factors such as HIV, and socio-economic factors.

For peer review only

Nested case-control study

A nested case control design will be utilised to investigate mechanisms with advanced laboratory testing on stored samples for efficiency. Cases will be defined as i) children who died during follow up and ii) children who are readmitted to hospital. Controls will be selected from children who survive to 6 months without readmission ("pure controls"), matched by site and nutritional strata to reflect the study design.

The primary analyses will estimate the association between the exposures of interest and the odds of poor outcomes during follow-up. The exposures of interest in these analyses will include plasma and faecal markers of intestinal dysfunction, systemic inflammation, systemic and enteric pathogens, 'maturity' and diversity of the enteric microbiome, proteomic and metabolomic markers of energy metabolism, and macro- and micronutrient status.

If mortality in the study is lower than expected (500 observed deaths), the primary case definition will be expanded to include children who were enrolled but were re-hospitalised post-discharge. Samples from these children may also be used in a sensitivity analysis to determine if associations with re-admission with severe illness ("near-miss") differ from those for mortality.

Sample size

Sample size calculations are based on expected events (deaths) in the cohort. We anticipate based on prior information from the sites that recruiting approximately 4,500 children in the specified strata will result in 500 deaths in this population. The sample size estimation is based on detecting differences in the proportion who die post-discharge between moderately malnourished and non-malnourished groups. Among non-malnourished children, we assumed an inpatient case fatality proportion of 5.0%, a cumulative post-discharge mortality of 2.5% compared to 7.5% inpatient case fatality among moderately malnourished children and allowing for 10% loss-to follow-up. A two-sided hypothesis that Ha: $p2 \neq p1$, with an alpha of 0.05, a power of 80% is attained for a post-discharge case fatality of 4.8% or above.

For laboratory analyses on stored samples, a nested case control approach will compare cases (children who die) and controls (children with good recovery) in a 1:2 ratio matched by site and nutritional strata. We expect approximately 500 deaths will occur, with 1000 controls. For a two-sided hypothesis that Ha: $p2 \neq p1$ at 80% power and a significance level of 0.05, this allows determination of an odds ratio of 1.8 for risk factors with a prevalence of 5% among controls, and of 1.4 for risk factors with a prevalence of 25%. Further analyses using a combined secondary endpoint of death and/or re-hospitalisation will ensure adequate power to detect risk factors with lower prevalence.

At each site, inclusion of 125 community participants will permit the calculation of descriptive percentages as integer values and estimates of community norms of continuous variables. (29) Across the whole study, 1,025 community participants will be recruited. For a two-sided hypothesis that Ha: $p2 \neq p1$, at 80% power and a significance level of 0.05 and assuming clustering by site, this sample size allows determination of a prevalence ratio of 1.5 for risk factors with a prevalence of 5% among community participants, and of 1.25 for risk factors with a prevalence of 25%.

Study timeline

The CHAIN Cohort began enrolling on 20th November 2016 and participant recruitment and follow up is expected to occur through August 2019.

For peer review only - http://bmjopen.bmj.com/site/about/guidelines.xhtml

Potential challenges and limitations

The study is designed and powered to detect associations with mortality, the primary outcome. Prior data from other studies conducted at the CHAIN sites suggest that sufficient numbers of events will occur to achieve adequate power. However, an interim analysis will be conducted in 2019 to confirm that the cohort study is adequately powered to detect effects of covariates of interest. In the event that mortality or enrolment rates are not sufficient, we will include re-hospitalisations as a combined primary endpoint. This cohort study is being conducted in low-resource environments where the risk of civil, political or military disruption, and industrial action affecting hospitals are significant. The inclusion of multiple sites across a wide geographic range allows for some sites to strategically increase enrolment if other sites are unable to achieve planned targets.

Bias may occur if children lost to follow up are not representative of the study population, it is anticipated that non-attenders may be more vulnerable. There is also the risk of the Hawthorne effect where involvement in the study alters outcomes.

Ethics and dissemination of results

This study protocol was reviewed and approved by the Oxford Tropical Research Ethics Committee, UK; the Kenya Medical Research Institute, Kenya; the University of Washington and Oregon Health and Science University, USA; Makerere University School of Biomedical Sciences Research Ethics Committee and The Uganda National Council for Science and Technology, Uganda; Aga Khan University, Pakistan; the International Centre for Diarrheal Disease Research, Bangladesh; The University of Malawi; The University of Ouagadougou and Centre Muraz, Burkina Faso; the Hospital for Sick Children, Canada; and University of Amsterdam, The Netherlands. This study is registered with clinicaltrials.gov (NCT03208725).

Prior to project inception, key stakeholders at each site were engaged including those from relevant Ministries of Health, local academic institutions, hospitals hosting the study and the community engaged in the research. Community Advisory Boards have been assembled at each site. Study progress and results are shared with these Key Stakeholders as well as disseminated through workshops and written materials. The CHAIN Network has both ethics and policy advisory boards to providence guidance on how to tailor research activities and help disseminate key findings with enough reach and power to influence high level policy decisions.

Acknowledgments

The CHAIN Network thanks all the patients and their families for participating in this study. We acknowledge all leadership and staff at CHAIN hospital sites, and especially the clinical staff for sharing their resources and time to both care for CHAIN patients and collect pertinent data for the study.

Author Contributions

Judd Walson and the CHAIN Network participated in proposal and protocol development.

Funding Statement

For peer review only

This work was supported by the Bill and Melinda Gates Foundation, grant number OPP1131320.

Data Statement

At the end of the study, a technical appendix, statistical code, and restricted dataset will available on the CHAIN Network website: http://chainnetwork.org/.

Competing Interests Statement

None of the authors or study co-investigators have any competing interests to declare.

For peer review only

REFERENCES

1. WHO. Children: reducing mortality World Health Organization: WHO; 2018 [updated 9/18/2018; cited 2018 10/24/2018]. Available from: http://www.who.int/news-room/fact-sheets/detail/children-reducing-mortality.
2. Black RE, Victora CG, Walker SP, Bhutta ZA, Christian P, de Onis M, et al. Maternal and child undernutrition and overweight in low-income and middle-income countries. Lancet. 2013;382(9890):427-51.
3. Wiens MO, Pawluk S, Kissoon N, Kumbakumba E, Ansermino JM, Singer J, et al. Pediatric post-discharge mortality in resource poor countries: a systematic review. PloS one. 2013;8(6):e66698.
4. Man WD, Weber M, Palmer A, Schneider G, Wadda R, Jaffar S, et al. Nutritional status of children admitted to hospital with different diseases and its relationship to outcome in The Gambia, West Africa. Tropical medicine & international health : TM & IH. 1998;3(8):678-86.
5. CHERG. Underlying Causes of Child Death CHERG [cited 2018 10/24/2018]. Available from: http://cherg.org/projects/underlying_causes.html.
6. Kerac M, Bunn J, Seal A, Thindwa M, Tomkins A, Sadler K, et al. Probiotics and prebiotics for severe acute malnutrition (PRONUT study): a double-blind efficacy randomised controlled trial in Malawi. Lancet. 2009;374(9684):136-44.
7. Maitland K, Berkley JA, Shebbe M, Peshu N, English M, Newton CR. Children with severe malnutrition: can those at highest risk of death be identified with the WHO protocol? PLoS medicine. 2006;3(12):e500.
8. Talbert A, Thuo N, Karisa J, Chesaro C, Ohuma E, Ignas J, et al. Diarrhoea complicating severe acute malnutrition in Kenyan children: a prospective descriptive study of risk factors and outcome. PloS one. 2012;7(6):e38321.
9. Fergusson P, Tomkins A. HIV prevalence and mortality among children undergoing treatment for severe acute malnutrition in sub-Saharan Africa: a systematic review and meta-analysis. Trans R Soc Trop Med Hyg. 2009;103(6):541-8.
10. Kerac M, Bunn J, Chagaluka G, Bahwere P, Tomkins A, Collins S, et al. Follow-up of post-discharge growth and mortality after treatment for severe acute malnutrition (FuSAM study): a prospective cohort study. PloS one. 2014;9(6):e96030.
11. Moisi JC, Gatakaa H, Berkley JA, Maitland K, Mturi N, Newton CR, et al. Excess child mortality after discharge from hospital in Kilifi, Kenya: a retrospective cohort analysis. Bulletin of the World Health Organization. 2011;89(10):725-32, 32A.
12. Chisti MJ, Graham SM, Duke T, Ahmed T, Faruque AS, Ashraf H, et al. Post-discharge mortality in children with severe malnutrition and pneumonia in Bangladesh. PloS one. 2014;9(9):e107663.
13. Chang CY, Trehan I, Wang RJ, Thakwalakwa C, Maleta K, Deitchler M, et al. Children successfully treated for moderate acute malnutrition remain at risk for malnutrition and death in the subsequent year after recovery. J Nutr. 2013;143(2):215-20.
14. Heikens GT, Bunn J, Amadi B, Manary M, Chhagan M, Berkley JA, et al. Case management of HIV-infected severely malnourished children: challenges in the area of highest prevalence. Lancet. 2008;371(9620):1305-7.
15. Chen LC, Chowdhury AA, Huffman SL. Anthropometric assessment of energy-protein malnutrition and subsequent risk of mortality among preschool aged children. The American journal of clinical nutrition. 1980;33(8):1836-45.
16. Isanaka S, Langendorf C, Berthé F, Gnegne S, Li N, Ousmane N, et al. Routine amoxicillin for uncomplicated severe acute malnutrition in children. New England Journal of Medicine. 2016;374(5):444-53.
17. Isanaka S, Langendorf C, Berthe F, Gnegne S, Li N, Ousmane N, et al. Routine Amoxicillin for Uncomplicated Severe Acute Malnutrition in Children. The New England journal of medicine. 2016;374(5):444-53.
18. Ngari MM, Mwalekwa L, Timbwa M, Hamid F, Ali R, Iversen PO, et al. Changes in susceptibility to life-threatening infections after treatment for complicated severe malnutrition in Kenya. The American journal of clinical nutrition. 2018;107(4):626-34.
19. Bhutta ZA, Das JK, Rizvi A, Gaffey MF, Walker N, Horton S, et al. Evidence-based interventions for improvement of maternal and child nutrition: what can be done and at what cost? The lancet. 2013;382(9890):452-77.
20. WHO. Guideline: Updates on the management of severe acute malnutrition in infants and children. WHO Guidelines Approved by the Guidelines Review Committee. Geneva2013.
21. Picot J, Hartwell D, Harris P, Mendes D, Clegg AJ, Takeda A. The effectiveness of interventions to treat severe acute malnutrition in young children: a systematic review. Health Technol Assess. 2012;16(19):1-316.

For peer review only - http://bmjopen.bmj.com/site/about/guidelines.xhtml

22. WHO. Recommendations for Management of Common Childhood Conditions: Evidence for Technical Update of Pocket Book Recommendations: Newborn Conditions, Dysentery, Pneumonia, Oxygen Use and Delivery, Common Causes of Fever, Severe Acute Malnutrition and Supportive Care. Recommendations for Management of Common Childhood Conditions: Evidence for Technical Update of Pocket Book Recommendations: Newborn Conditions, Dysentery, Pneumonia, Oxygen Use and Delivery, Common Causes of Fever, Severe Acute Malnutrition and Supportive Care. WHO Guidelines Approved by the Guidelines Review Committee. Geneva 2012.
23. Angood C, McGrath M, Mehta S, Mwangome M, Lung'aho M, Roberfroid D, et al. Research Priorities to Improve the Management of Acute Malnutrition in Infants Aged Less Than Six Months (MAMI). PLoS medicine. 2015;12(4):e1001812.
24. Bhutta ZA, Berkley JA, Bandsma RHJ, Kerac M, Trehan I, Briend A. Severe childhood malnutrition. Nat Rev Dis Primers. 2017;3:17067.
25. Berkley J, Mwangi I, Griffiths K, Ahmed I, Mithwani S, English M, et al. Assessment of severe malnutrition among hospitalized children in rural Kenya: comparison of weight for height and mid upper arm circumference. JAMA : the journal of the American Medical Association. 2005;294(5):591-7.
26. Myatt M, Khara T, Collins S. A review of methods to detect cases of severely malnourished children in the community for their admission into community-based therapeutic care programs. Food and nutrition bulletin. 2006;27(3 Suppl):S7-23.
27. WHO. Verbal autopsy standards: the 2012 WHO verbal autopsy instrument. Geneva: WHO. 2012.
28. Hedges A. Random Number Generator / Picker 2008 [cited 2018 10/24/2018]. Available from: https://andrew.hedges.name/e/random/.
29. Horowitz GL, Altaie S, Boyd JC. Defining, establishing, and verifying reference intervals in the clinical laboratory; approved guideline: CLSI; 2010.

For peer review only

For peer review only - http://bmjopen.bmj.com/site/about/guidelines.xhtml

Collaborators

Full Name	Affiliations	Role
Berkley, JA	KEMRI/Wellcome Trust Research Programme, Kilifi, Kenya.<br><br>Center for Tropical Medicine and Global Health, University of Oxford, Oxford, United Kingdom.	Network PI
Walson, JL	Departments of Global Health, Medicine, Paediatrics and Epidemiology, University of Washington, Seattle, Washington, USA.	Network PI
Diallo, AH	Department of Public Health, Faculty of Health Sciences, University of Ouagadougou I Pr Joseph KI-ZERBO, Burkina Faso;<br><br>Department of Public Health, Centre MURAZ Research Institute, Ministry of Health, Bobo-Dioulasso, Burkina Faso.	Site PI/Co-Investigator
Shahid, ASMSB	Nutrition and Clinical Services Division (NCSD), International Centre for Diarrhoeal Disease Research, Bangladesh (icddr,b), Dhaka, Bangladesh.	Co-Investigator
Gwela, A	KEMRI/Wellcome Trust Research Programme, Kilifi, Kenya	Co-Investigator
Saleem, A	Department of Pediatrics and Child Health, The Aga Khan University, Karachi, 74800, Pakistan	Site PI/Co-Investigator
Asad, A	Department of Pediatrics and Child Health, The Aga Khan University, Karachi, 74800, Pakistan	Co-Investigator
Tigoi, CC	KEMRI/Wellcome Trust Research Programme, Kilifi, Kenya	Co-Investigator
Bourdon, C	The Hospital for Sick Children, Department of Translational Medicine, Toronto, Canada	Co-Investigator
Lancioni, CL	Department of Pediatrics, Oregon Health and Science University, Portland, OR, USA	Co-Investigator
Denno, DM	Department of Pediatrics, School of Medicine, University of Washington, Seattle, WA. USA.<br><br>Department of Global Health and Department of Health Services, School of Public Health, University of Washington, Seattle, WA. USA.	Co-Investigator
Mangale, DI	Department of Global Health, University of Washington, Seattle, WA. USA.	Co-Investigator
Mupere, E	Department of Paediatrics and Child Health, College of Health Sciences, Makerere University, Kampala, Uganda.	Site PI/Co-Investigator
Tickell, KD	Departments of Global Health, and Epidemiology, University of Washington, Seattle, Washington, USA.	Co-Investigator
Mwangome, MK	KEMRI/Wellcome Trust Research Programme, Kilifi, Kenya	Co-Investigator
Chisti, MJ	Nutrition and Clinical Services Division (NCSD), International Centre for Diarrhoeal Disease Research, Bangladesh (icddr,b), Dhaka, Bangladesh.	Site PI/Co-Investigator
Ngari, MM	KEMRI/Wellcome Trust Research Programme, Kilifi, Kenya.	Co-Investigator
Ngao, NM	KEMRI/Wellcome Trust Research Programme, Kilifi, Kenya	Co-Investigator
Sukhtankar, P	KEMRI/Wellcome Trust Research Programme, Kilifi, Kenya	Co-Investigator
Bandsma, RHJ	Centre for Global Child Health, Hospital for Sick Children, Toronto, Ontario Canada.<br><br>Division of Gastroenterology, Hepatology and Nutrition, Hospital for Sick Children, Toronto, Ontario Canada.	Site PI/Co-Investigator

For peer review only

	Department of Nutritional Sciences, Faculty of Medicine, University of Toronto, Toronto, Ontario Canada.<br><br>Department of Biomedical Sciences, College of Medicine, University of Malawi, Blantyre, Malawi.	
Molyneux, S	KEMRI Centre for Geographic Medicine Research - Coast, and Wellcome Trust Research Programme, Nairobi, Kenya.<br><br>Centre for Tropical Medicine, University of Oxford, Oxford, UK.	Co-Investigator
Ahmed, T	Nutrition and Clinical Services Division (NCSD), International Centre for Diarrhoeal Disease Research, Bangladesh (icddr,b), Dhaka, Bangladesh.	Site PI/Co-Investigator
Voskuijl, W	Department of Paediatrics and Child Health, College of Medicine, University of Malawi, Blantyre, Malawi.<br><br>Department of Biomedical Sciences, College of Medicine, University of Malawi, Blantyre, Malawi.	Site PI/Co-Investigator

For peer review only

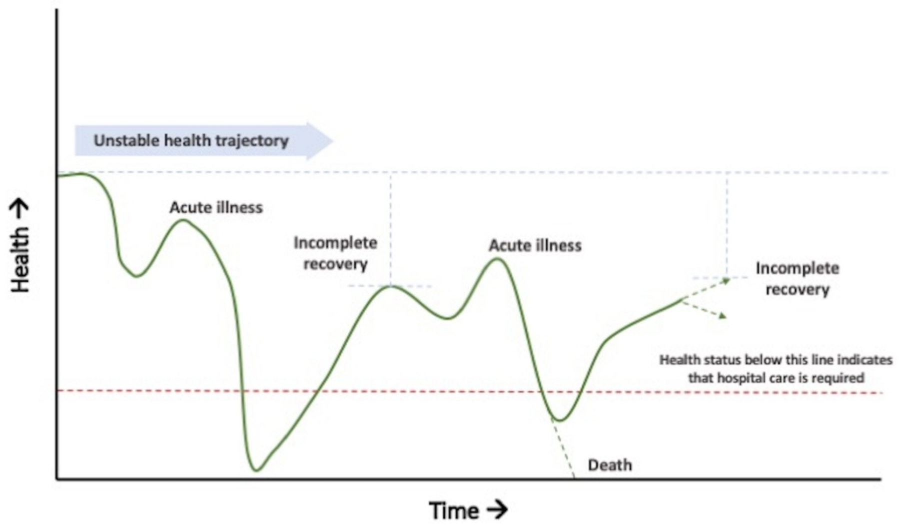

Figure 1: A model of unstable health trajectory of a child characterised by repeated illness and incomplete recovery

277x191mm (300 x 300 DPI)

CHAIN Enrolment CRF v1.63

CHAIN Number [1][0] [0][0][3] [ ][ ][ ]

Eligibility Checklist		
**Age between 2 months and before 2nd birthday**	Y	N - ineligible
**Being admitted to hospital because of acute illness**	Y	N- ineligible
**Parent or guardian able and available to consent**	Y	N- ineligible
**Able to feed orally in usual state of health**	Y	N- ineligible
**Known congenital syndrome**	Y- ineligible	N
**Cleft palate**	Y- ineligible	N
**Known congenital cardiac disease**	Y- ineligible	N
**Known terminal illness e.g. cancer**	Y- ineligible	N
**Admission for surgery, or likely to require surgery within 6m**	Y- ineligible	N
**Admission for trauma?**	Y- ineligible	N
**Sibling enrolled in study**	Y- ineligible	N
**Previously enrolled**	Y- ineligible	N

Part 1

Admission to Hospital and Study Enrolment					
**DATE arrived at the hospital**	__ __/__ __/__ __ __ __ D D / M M / Y Y Y Y	**TIME arrived at the hospital**	__ __: __ __ *24h Clock*	☐ Arrival time unknown	
**DATE of enrolment** i.e. date consented and seen by research team	__ __/__ __/__ __ __ __ D D / M M / Y Y Y Y	**TIME of enrolment**	__ __: __ __ *24h Clock*	**Sex**	☐ Male ☐ Female
**DOB**	__ __/__ __/__ __ __ __ D D / M M / Y Y Y Y	**Is the DOB:**	☐ True ☐ Estimated*	**Child's Initials**	___ ___ ___
**Brought into hospital by:** *Select all that apply*	☐ Mother	☐ Father	☐ Grandparent	☐ Aunt/Uncle	
	☐ Sibling <18	☐ Sibling >18	☐ Carer (care home)	☐ Other__________	

**if DOB is estimated, and the day is uncertain, write '15' for DD*

Presenting Complaints

☐ Fever / Hotness of body	☐ Vomiting	☐ Lethargy
☐ Difficulty breathing	☐ Diarrhoea <14 days	☐ Convulsions
☐ Cough<14 days	☐ Diarrhoea >14 days	☐ Altered consciousness
☐ Cough>14days	☐ Blood in stool	☐ Not feeding
☐ Poor feeding/ Weight loss	☐ Developmental delay	☐ Body swelling / limb swelling/ Oedema
☐ Rash/ skin lesion	☐ Other *(only one complaint, if not covered by above options)* ______________________________________	

For peer review only

For peer review only - http://bmjopen.bmj.com/site/about/guidelines.xhtml

CHAIN Enrolment CRF v1.63

CHAIN Number [1][0] [0][0][3] [ ][ ][ ]

**Initial Observations** (to be taken at time of examination by research team)			
**Axillary temperature**	____ ____. ____ °C	**Respiratory rate** *Count for 1 minute*	____ ____ ____/minute
**Heart rate** *Count for 1 minute*	____ ____ ____/minute		
**SaO2** *To be taken from finger or toe using pulse oximeter*	____ ____ ____ % *Leave blank if unrecordable*	☐ Measured in Oxygen ☐ Measured in Room Air	☐ Unrecordable

**Anthropometry**					
**Weight** *to be taken using SECA scales for CHAIN study*	___ ___ . ___ ___kg	**Length** *to be taken using SECA 416 infantometer provided for CHAIN study*	Measurer 1	____ ____. ____ cm	
			Measurer 2	____ ____ . ____ cm	
**MUAC** *To be taken using MUAC tape for CHAIN study*	Measurer 1	____ ____ . ____ cm	**Head circumference** *To be taken using CHAIN measuring tape*	Measurer 1	____ ____. _____cm
	Measurer 2	____ ____ . ____ cm		Measurer 2	____ ____. _____cm
**Oedema**	☐ None ☐ + ☐ ++ ☐ +++		**Initials**	Measurer 1 ___ ___ ___	Measurer 2 ___ ___ ___

NB: If the child is unwell the Length and Head Circumference can be taken at a later time.

**Current Health**				
**Previously admitted to hospital.** *Include other hospitals / health centres. Select 1*	☐ No	☐ < 1 week ago	☐ 1 weeks-1month ago	☐ >1month ago
**Any medication last 7 days.** *Select all that apply*	☐ No medication	☐ Antibiotic	☐ Antimalarial	☐Traditional
	☐ Deworming	☐ Vitamin	☐ Paracetamol or Ibuprofen	
	☐ Yes, but unknown		☐Other	
**Urine volume in last 24hrs?** *Select 1*	☐ Not passing urine	☐ Less than normal	☐ Normal or greater	☐ Unknown

For peer review only - http://bmjopen.bmj.com/site/about/guidelines.xhtml

CHAIN Enrolment CRF v1.63
CHAIN Number [1][0] [0][0][3] [ ][ ][ ]

Examination

Examination should be performed by CHAIN study clinician trained in clinical examination of children, and able to formulate a diagnosis based on clinical history and findings. Refer to Clinical Examination SOP

**Airway** *(select one)*	☐ **Clear**	☐ Needs active support		☐ Obstructed/Stridor	
**Breathing** *(select all that apply)*	☐ **Normal – no concerns**, (move to circulation)				
	☐ Central cyanosis	☐ Nasal flaring		☐ Reduced air-entry	
	☐ Wheeze	☐ Acidotic Breathing		☐ Grunting	
	☐ Lower chest wall indrawing	☐ Crackles		☐ Dull to percussion<br>☐ Head nodding	
**Circulation:**					
**Cap Refill** (select one)	☐ >3s	☐ 2-3s	☐ <2s		
**Cold Peripheries**(select one)	☐ Shoulder	☐ Elbow	☐ Hand	☐ Warm peripheries	
**Disability:**					
**Conscious level**(select one)	☐ **Alert**	☐ Voice	☐ Pain	☐ Unresponsive	
**Fontanelle**(select one)	☐ **Normal**	☐ Bulging	☐ Sunken	☐ Not present	
**Tone**(select one)	☐ **Normal**	☐ Hypertonic		☐ Hypotonic	
**Posture**(select one)	☐ **Normal**	☐ Decorticate		☐ Decerebrate	
**Activity**(select one)	☐ **Normal**	☐ Irritable/Agitated		☐ Lethargic	
**Dehydration:**					
**Sunken eyes?**	☐ Y	☐ N			
**Skin pinch** *(select one)*	☐ >2 seconds	☐ <2 seconds	☐ Immediate		
**Drinking/Breastfeeding** *(Select one)*	☐ **Normal**	☐ Poorly	☐ Not drinking	☐ Eager / Thirsty	
**Abdomen** *(select any that apply)*	☐ **Normal – no concerns**	☐ Distension	☐ Hepatomegaly		
	☐ Tenderness	☐ Splenomegaly	☐ Other abdominal mass		
**Signs of Rickets**	☐ **None**	☐ Wrist widening	☐ Rachitic rosary	☐ Swollen knees	☐ Bow legs	☐ Frontal bossing
**Jaundice** *(Select one)*	☐ **Not jaundiced**	☐ +	☐ ++	☐ +++	
**ENT/Oral/Eyes** *(select any that apply)*	☐ Mouth Normal	☐ Ears Normal		☐ Eyes Normal	
	☐ Oral ulceration	☐ Pus from ear		☐ Conjunctivitis	
	☐ Oral candidiasis	☐ Tender swelling behind ear (mastoiditis)		☐ Eye discharge	
	☐ Stomatitis	☐ Lymphadenopathy		☐ Visual impairment	
**Skin** *(select any that apply)*	☐ Normal	☐ Hyperpigmentation		☐ Depigmentation	
	☐ Broken skin	☐ Dermatitis		☐ 'Flaky paint'	
	☐ Cellulitis	☐ Impetigo		☐ Pustules	
	☐ Vesicles	☐ Desquamation		☐ Macular or papular	
**Site of skin lesions.** *(select any that apply)*	☐ Not applicable (No rash)	☐ Trunk	☐ Face / scalp	☐ Legs	
	☐ Palms / soles	☐ Buttocks	☐ Arms	☐ Perineum	

For peer review only - http://bmjopen.bmj.com/site/about/guidelines.xhtml

CHAIN Enrolment CRF v1.63
CHAIN Number [1][0] [0][0][3] [ ][ ][ ]

Suspected Chronic Conditions

Select confirmed, suspected or none for all conditions:	Confirmed *(diagnosed previously/ recorded)*	Suspected *(clinician's impression)*	None
**Cerebral palsy/neurological problem/ epilepsy**	☐	☐	☐
**Sickle Cell disease** *family history, crisis*	☐	☐	☐
**Thalassaemia**	☐	☐	☐
**Visual problem / Blindness** *Not fixing and following*	☐	☐	☐
**Losing weight or not gaining weight**	☐	☐	☐

TB Screening

Known TB (on treatment)	Child has cough >14 days	Household contact has TB, or cough >14 days	Child has suspected extra-pulmonary TB
Y N	Y N	Y N	Y N

Feeding

**Currently in outpatient nutrition program?** *Select one.*	☐ Supplementary *(corn soy blend, RUSF, khichuri, halwa)*	☐ Therapeutic *(RUTF, Plumpy-nut)*	☐ None
**Has the child eaten these nutrition products in the last 3 days?**	☐ Supplementary	☐ Therapeutic	☐ None
**Currently Breastfeeding?**	☐ Y ☐ N	**If yes is the child taking anything else (exclude medicine)?**	☐ Y ☐ N ☐ N/A
**If NO breastfeeding at all, age stopped in months?** *(select one)*	☐ 0-3m ☐ 4-6m ☐ 7-12m	☐ >12m ☐ Unknown	☐ N/A
**What did the child receive other than breast milk in the first 3 days of life?** *Select all that apply* *Do not include medications e.g. ARV.*	☐ Sweetened/sugar water<br>☐ Fruit Juice<br>☐ Water<br>☐ Pure Honey	☐ Formula/powder milk<br>☐ Tea<br>☐ Porridge/pulp<br>☐ Glycerine	☐ Animal milk<br>☐ Other<br>☐ Gutthi / gripe water<br>☐ Nothing

Vaccinations – *Ask carer or check book / card if available*

**BCG scar**	☐ Yes ☐ No	**Rotavirus**	☐ Book	☐ Self report	☐ Not received	**Doses received:**	3 2 1 ☐ Unknown
**Measles**	☐ Book ☐ Self report ☐ Not received ☐ Unknown	**Pneumococcus**	☐ Book	☐ Self report	☐ Not received	**Doses received:**	3 2 1 ☐ Unknown
		**DTP/Penta**	☐ Book	☐ Self report	☐ Not received	**Doses received:**	3 2 1 ☐ Unknown
		**Polio**	☐ Book	☐ Self report	☐ Not received		☐ Unknown

For peer review only - http://bmjopen.bmj.com/site/about/guidelines.xhtml

CHAIN Enrolment CRF v1.63

CHAIN Number [1][0] [0][0][3] [ ][ ][ ]

CLINICIANS IMPRESSION OF RISK						
How likely does the clinical team think this child is to die during this admission? Select one						
☐ Almost certainly not	☐ Very unlikely	☐ Quite unlikely	☐ Unsure	☐ Quite likely	☐ Very likely	☐ Almost certainly

Immediate Clinical Investigations and HIV status						
**Malaria RDT** *circle result*	Positive		Negative		Not done	
**Blood glucose**	___ ___ . ___ mmol/L		**Time glucose measured**		___ ___:___ ___ *24h clock* ☐ Unknown	
**Urine Dipstick** *(can be done at any time during admission)*<br>**Urine sample stored?** Y N	Protein	Nitrites	Leucocytes	Blood	Ketones	Glucose
☐ Not done ☐ Bag ☐ Clean catch	None<br>+ ++ +++	Pos Neg	None<br>+ ++ +++	None<br>+ ++ +++	None<br>+ ++ +++	None<br>+ ++ +++

**HIV status known?**		☐ Yes, known PCR positive<br>☐ Yes, antibody positive, unknown PCR status<br>☐ Yes, known exposed, known PCR negative (children under 18m with PCR result SEEN BY RESEARCH TEAM. If not seen select below and perform HIV RDT<br>☐ No, known to be HIV exposed, but child untested<br>☐ No, child not tested, not known to be exposed		
**If child known HIV positive or exposed**	**On any ART?**	☐ Y ☐ N ☐ Unknown	**If on treatment,**<br>ARV 1___________________<br>ARV 2___________________<br>ARV 3___________________	**If on prophylaxis**<br>☐ Nevirapine prophylaxis only<br>☐ AZT + NVP prophylaxis<br>☐ Caregiver unsure
	**Co-trimoxazole** *select one*	☐ On prophylactic dose co-trimoxazole<br>☐ On high dose co-trimoxazole	☐ Not on co-trimoxazole	☐ Caregiver unsure
**If not known positive**	**HIV RDT now** *select one*	☐ Reactive / positive<br>PCR sent: ☐ Y ☐ N	☐ Non-Reactive / Negative	☐ Declined
**HIV test offered to caregiver?**		☐ Yes, Reactive ☐ Yes, Non-reactive ☐ Yes, but Declined	☐ No, Caregiver is known positive	☐ Missed ☐ N/A child in care home
**Did the mother have interventions or medication during delivery to prevent transmission of HIV to baby?**		☐ Yes	☐ No	☐ Unknown

For peer review only - http://bmjopen.bmj.com/site/about/guidelines.xhtml

CHAIN Enrolment CRF v1.63

CHAIN Number [1][0] [0][0][3] [ ][ ][ ]

INITIAL TREATMENT			
**Admitted to:** *select one*	☐ Admission to ward	☐ Admission to HDU	☐ Admission to ICU
**Date and time First antibiotics given**	__ __/__ __/__ __ __ __ *24h clock*	___ ___:___ ___	☐Not given
**Intravenous Antibiotics Given?** ☐ **Not given**	☐ Benzylpenicillin	☐ Gentamicin	☐ Ceftriaxone / Cefotaxime
	☐ Co-amoxiclav/ Augmentin	☐ Flu/Cloxacillin	☐ Chloramphenicol
	☐ Ampicillin	☐ Amikacin	☐ Meropenem / Imipenem
	☐ Levofloxacin	☐ Vancomycin	☐ Metronidazole
	☐ Ceftazidime	☐ Pivmecillinam	
	☐ Other________________________		
**Oral Antibiotics Given?** ☐ **Not given**	☐ Amoxicillin	☐ Erythromycin	☐ Azithromycin
	☐ Co-trimoxazole	☐ Metronidazole	☐ Ciprofloxacin
	☐ Cefalexin / cefaclor	☐ Co-amoxiclav / Augmentin	☐ Nalidixic acid
	☐ Penicillin	☐ Flucloxacillin	☐ Levofloxacin
	☐ Other _______________		

**Initial treatment <u>given</u>** *First 6 hours. Select any that apply. For IV fluid bolus, and IV fluids specify type and volume in ml, and duration*	☐ IV Fluid Bolus	☐ IV Maintenance Fluids
	☐ Oxygen	☐ CPAP
	☐ IV Glucose ☐ Oral Glucose	☐ Warmth (heater, warmed fluids)
	☐ Blood transfusion	☐ Commercial F75
	☐ Phenobarbitone	☐ Commercial F100
	☐ Diazepam	☐ Locally prepared F75/ milk suji
	☐ Paracetamol	☐ Local prepared F100 / milk suji 100
	☐ Ibuprofen	☐ Expressed breast milk
	☐ Diclofenac	☐ Dilute F100/ dilute milk or formula
	☐ Salbutamol / atrovent / other bronchodilator	☐ Other milk/ formula/ feed<br>☐ RUTF
	☐ Prednisolone/ dexamethasone/ hydrocortisone	☐ Nasogastric tube
	☐ Adrenaline	☐ Multivitamin
	☐ Zinc	☐ Micronutrients
	☐ Folic acid	☐ Vitamin A
	☐ Antimalarial (any)	☐ Albendazole / deworming
	☐ ReSoMal	☐ Other ____________________
	☐ ORS	

For peer review only - http://bmjopen.bmj.com/site/about/guidelines.xhtml

CHAIN Enrolment CRF v1.63

CHAIN Number [1][0] [0][0][3] [ ][ ][ ]

Suspected Initial Diagnoses:

Clinical diagnosis should be based on examination and investigation findings.

Tick the three most likely diagnoses.

**Respiratory**	**Infection**	**CNS**
☐ LRTI/pneumonia	☐ Gastroenteritis	☐ Febrile convulsions
☐ Bronchiolitis	☐ Sepsis	☐ Epilepsy
☐ URTI	☐ Malaria	☐ Probable meningitis
☐ Pulmonary TB	☐ Extra pulmonary TB	☐ Other encephalopathy
☐ Otitis media	☐ Soft tissue infection	☐ Hydrocephalus
☐ Asthma	☐ UTI	☐ Developmental delay
**General**	☐ HIV related illness	☐ Cerebral palsy
☐ Anaemia	☐ Measles	**Other suspected diagnosis:**
☐ Sickle Cell Disease	☐ Varicella	☐ Other
☐ Thalassaemia	☐ Osteomyelitis	☐ Unknown
☐ Renal impairment	☐ Febrile illness unspecified	☐ Failed appetite test only
☐ Nephrotic syndrome	☐ Enteric fever	
☐ Nephritis		
☐ Liver dysfunction		
☐ Ileus		
☐ Congenital cardiac disease		

For peer review only

For peer review only - http://bmjopen.bmj.com/site/about/guidelines.xhtml

CHAIN Enrolment CRF v1.63
CHAIN Number [1][0] [0][0][3] [ ][ ][ ]

Admission Core Cohort Investigations and Sample Collection					
**CBC taken**	☐ Y	☐ N	**Plain Blood (serum)**	☐ Y	☐ N
**Clinical chemistry taken**	☐ Y	☐ N	**Blood spot taken**	☐ Y	☐ N
**EDTA 2ml blood taken**	☐ Y	☐ N	**Blood culture taken** *(if available at site)*	☐ Y BEFORE ABX ☐ Y AFTER ABX	☐ N
**EDTA 0.5ml blood taken**	☐ Y	☐ N	**Blood gas taken** *(if available at site)*	☐ Capillary ☐ Venous	☐ N
**Unable to take blood samples, why?**	☐ Difficult venepuncture	☐ Child uncooperative	☐ Parent refused	☐ Other	
**Rectal swabs taken**	☐ Y BEFORE ABX ☐ Y AFTER ABX	☐ N	Number taken ☐1 ☐2	Time taken___ ___: ___ ___	
**Stool sample**	Taken in first 24h?	☐ Y	☐ N	Time taken___ ___: ___ ___	

**Chest x-ray indicated** *(respiratory signs symptoms)*	☐ Yes, but too unwell	☐ Yes, done	☐ Indicated but not done, unclear	☐ Not indicated
**Lumbar puncture indicated** *(signs of meningitis documented)*	☐ Yes, but too unwell	☐ Yes, done		☐ Not indicated

**Blood Samples taken by (initials)**	__ __ __
**Rectal Swabs taken by (initials)**	__ __ __

**CRF Completed by (Initials) – to be signed when complete.** *Do not sign if any fields are empty*	__ __ __	Date __ __/__ __/__ __ __ __ *D D / M M / Y Y Y Y*	Time ___ ___: ___ ___

For peer review only - http://bmjopen.bmj.com/site/about/guidelines.xhtml

CHAIN Enrolment CRF v1.63

CHAIN Number [1][0] [0][0][3] [ ][ ][ ]

PART 2

CHAIN ADMISSION CRF: SOCIAL INFORMATION.

To be completed within 48h of admission when child is stable. This should ideally be done in a conversational and unhurried way, with the interviewer sitting with the caregiver.

Initials of person interviewing caregiver and completing part 2	Date
___ ___ ___	__ __/ __ __/ __ __ __ __<br>D D/ M M/ Y Y Y Y
☐ Doctor ☐ Clinical officer ☐ Nurse ☐ Field worker ☐ Research Assistant ☐ Other	Time<br>___ ___: ___ ___

Who is being interviewed?

☐ Primary caregiver only ☐ Care home staff ☐ Primary caregiver and one other person ☐ Primary caregiver and more than one other person ☐ One person who is not the primary caregiver ☐ More than one person who is not the primary caregiver

Care-seeking Behaviour

**Was the child in generally good health before this illness?**	☐ Y	☐ N	☐ Unknown
**If No, how long has the child had this problem of generally bad health?**	__ __ weeks	☐ N/A	
**Does the child have health insurance?**	☐ Y	☐ N	☐ Unknown

What was the main reason for bringing the child to this hospital today? *Reasons given, select one*

☐ Referred by health care worker ☐ Caregiver concern of child's condition ☐ Received money for transport to hospital (e.g. from family, neighbour, paid work)?

☐ Relative / neighbour concern of child's condition ☐ Primary caregiver returned home e.g. if working away ☐ Other

How did you travel to the hospital? *Select all that apply*

☐Car/ Taxi ☐ Ambulance ☐ Bus ☐Motorbike ☐ Tuk-tuk /CNG ☐ Cycle rickshaw ☐ Train ☐ Walking ☐ Other

How long did it take you to travel to hospital? ☐ <1h ☐ 1- < 2h ☐ 2-4h ☐ >4h ☐ > 1 day

How much did it cost the family to travel to hospital today (in local currency? *Estimate amount. If walked or free ambulance write 0* ___ ___ ___ ___

Have you sought treatment for this illness prior to coming to hospital? *Select all that apply*

☐ No treatment sought ☐ Shop ☐ Government hospital ☐ Government dispensary ☐ Traditional Healer

☐ Pharmacy ☐ Private Medical Facility/ NGO ☐ Herbalist ☐ Homeopathist ☐ Other

Received treatment from traditional healer, homeopathist or herbalist in last 4 weeks? Y N

Child's Health Status Before Admission

Before this illness, how did this child's health compare to other children of similar age in your neighbourhood? *Select one*

☐Similar ☐Better ☐Worse ☐Don't know

Before this illness, how did this child's health compare to his/her siblings at a similar age? *Select one*

☐Similar ☐Better ☐Worse ☐ Don't know ☐ N/A only child

For peer review only - http://bmjopen.bmj.com/site/about/guidelines.xhtml

CHAIN Enrolment CRF v1.63
CHAIN Number [1][0] [0][0][3] [ ][ ][ ]

Birth History						
**Source of information**	☐ Maternal/caregiver recall		☐ Book/medical records			
**Birth weight**	___ .___ ___kg		☐Unknown			
**Birth details** *Select any that apply*	☐ Premature	☐ Born small <2.5kg	☐Twin/multiple birth	☐ Born at term	☐ Unknown	
**Delivery location** *Select one*	☐ Born in hospital	☐ Community facility/clinic with midwife/nurse midwife/doctor				
	☐ Home without birth attendant	☐ Home with traditional birth attendant (untrained)	☐ Home with midwife/nurse			
	☐ Other		☐ Unknown			
**Delivery details** *Select all that apply*	☐ Normal, spontaneous vaginal delivery	☐ Assisted delivery (forceps, ventouse)	☐ Caesarean section			
	☐ Admitted neonatal unit	☐ Mother admitted to hospital >48h	☐ Unknown			
**Mother's age at first pregnancy**	___ ___years	☐ unknown	**Mother's age now**	___ __years	☐ unknown	
**Participant birth order**	___ ___ of ___ ___ total live births (*e.g. if youngest of 3 children 3 of 3, if oldest of 3 children 1 of 3)*					
**Are the biological parents of this child consanguineous?** *Ask if parents have relatives in common or are related.*	☐ Yes	☐ No	☐ Unknown			

For peer review only - http://bmjopen.bmj.com/site/about/guidelines.xhtml

CHAIN Enrolment CRF v1.63
CHAIN Number [1][0] [0][0][3] [ ][ ][ ]

Primary Caregiver Information<br>*This is the person who has responsibility for day to day care of the child, but is not a substitute carer such as childminder or grandparent who cares for child whilst, for example, mother is at work.*			
**Who is the Primary Caregiver?** *Select one*	☐Biological Parent ☐Grandparent ☐Sibling ☐Aunt / Uncle / Cousin<br>☐ Stepmother / father ☐ Care home /orphanage ☐Other/ Unclear		
**Is the child's biological father alive?**	☐ Y ☐ N ☐ Unknown	**Is the child's biological mother alive?**	☐ Y ☐ N ☐ Unknown
**Primary Care Giver Age** *Select one*	☐ <18years ☐ >=18 years ☐ >50years ☐ N/A (care home or unclear)		
**Primary Care Giver Sex** *Select one*	☐ Male ☐ Female ☐ N/A	**Primary caregiver present at admission?**	☐ Y ☐ N
**Has the primary caregiver lived in the same household as the child for the last 2 months?**			☐ Y ☐ N ☐ N/A (care home)
**Marital status of primary caregiver** *Select one*	☐ Married/ monogamous ☐ Married polygamous ☐ Single ☐ Separated / divorced ☐ Widowed ☐ N/A		
**If not present at admission, where is the primary caregiver?** *Select one*			
☐ Home ☐ Work ☐ School ☐ Unknown ☐ Other_______ ☐ N/A			
**If the primary caregiver is present, caregiver anthropometry:**<br>*Use locally available adult scales and stadiometer, and adult MUAC tapes provided by CHAIN.*			
☐ **Primary caregiver not present during admission, or care home**			
**Weight** ___ ___. ___ kg	**MUAC** ___ ___ .____.cm	**Height:**___ ___ ___cm	
**Education:** *Select highest level of education achieved*	☐ None ☐ Primary ☐ Secondary ☐ Above secondary ☐ Unknown ☐ N/A care home		
**Able to read?**	☐ Y ☐ N ☐Unknow n	**Is the primary caregiver primarily responsible for financial support and providing for the child?**	☐ Y ☐ N
**Primary caregiver HIV status in last 6 months** *Select one*	☐ Tested Positive ☐ Tested Negative ☐ Not tested or unknown		

Have there been ANY changes to the child's social situation in the last 2 MONTHS? *Select any that apply,*

**Child moved to a different household**	Y N	**Relocation from rural to urban setting** *Select 'yes' even if this is temporary*	Y N
		**Relocation from urban to rural setting** *Select 'yes' even if this is temporary*	Y N
		**Relocation to live with different caregiver** *Select 'yes' even if this is temporary*	Y N
**Mother sick**	Y N	**Mother Died**	Y N
**Father sick**	Y N	**Father Died**	Y N
**Other primary caregiver sick**	Y N N/A	**Other primary caregiver died**	Y N N/A
**Primary caregiver changed**	Y N	**Child went into care home**	Y N
**Primary caregiver started employment / returned to school**	Y N	**Person providing for the child has lost income**	Y N
**Primary caregiver divorced / separated from partner**	Y N	**Primary caregiver in new relationship**	Y N
**Mother is pregnant**	Y N	**Mother gave birth**	Y N
**Other primary caregiver pregnant?**	Y N N/A	**Other primary caregiver gave birth**	Y N N/A

If primary caregiver has changed in the last 2 months, who was the child's previous primary caregiver? *Select one*

☐Biologic Mother	☐Biologic Father	☐Sibling ≥18 years old	☐Sibling <18 years old
☐Grandparent	☐Aunt/Uncle/Cousin	☐Other	☐ N/A

For peer review only - http://bmjopen.bmj.com/site/about/guidelines.xhtml

CHAIN Enrolment CRF v1.63
CHAIN Number [1][0] [0][0][3] [ ][ ][ ]

**Primary caregiver earns an income now?** *Ask the person accompanying the child and select one*	
☐ Employed full time by someone else	☐ Employed part time by someone else
☐ Works for self	☐ No work income
☐ Works casually/irregularly for someone	☐ Don't know
If works casually, Occupation:	☐ N/A care home

**How many days worked a week?** *Select one*	☐ **<3**	☐ **3-5**	☐ **>5**	☐ N/A, does not work for income

**If the primary caregiver earns, main source of income?** *Select one*			
☐ Farmer	☐ Business/trader	☐ Labourer	☐ Domestic work
☐ Other private sector employment	☐ Public sector employment	☐ Retired with pension income	
☐ Begging	☐ Other__________________	☐ N/A	

**If the primary caregiver works (earning or non-earning), main place of work?** *Select one*		
☐In/around home (where child lives)	☐ Away for <4 hours per day	☐Away >4 hours but comes home daily
☐Away > 8h a day but returns home daily	☐Away >1 day, comes home weekly	☐ Away comes home, less than weekly
☐Primary caregiver lives and works away	☐ Don't know	☐ N/A

**The person primarily providing financial support to this child is this child's:** *Select one*			
☐ Biologic Mother	☐ Biologic Father	☐ Stepfather	☐ Stepmother
☐ Grandparent	☐ Sibling ≥18 years old	☐ Sibling <18 years old	☐ Aunt/Uncle/Cousin
☐ More than one person responsible, unclear	☐ Unsupported / care home	☐ Other -specify_________________	

**Person responsible for providing financial support to child, place of usual residence**? *Select one*	
☐ Always sleeps at home	☐ Sleeps away but returns weekly
☐ Sleeps away for > two months per year	☐ Works and lives abroad, contact with child once a year or less
☐ Sleeps away but return monthly or less often	☐ Don't know
☐ Other____	☐ N/A (e.g. care home, unsupported)

**What is the Father or person responsible for providing financial support to child source of income**? *Select one. If the primary carer is also the person providing financial support do not complete this section.*			
☐ Farmer	☐ Business/trader	☐ Labourer	☐ Domestic work
☐ Other private sector employment	☐ Public sector employment	☐ Retired with pension income	
☐ Begging ☐None ☐ Unknown	☐ Other ______________________	☐ N/A	

For peer review only - http://bmjopen.bmj.com/site/about/guidelines.xhtml

CHAIN Enrolment CRF v1.63
CHAIN Number [1][0] [0][0][3] [ ][ ][ ]

**Substitute Care:** *Who usually looks after child when primary caretaker is working or away? Select all that apply*			
☐ Not applicable, caregiver looks after child full time		☐ Not applicable, child accompanies caregiver to work	
☐ No substitute care, child left alone		☐ No substitute care / unclear	☐ Child in care home
☐ Biological Mother	☐ Biological Father	☐ Sibling <18 years old	☐ Sibling ≥18 years old
☐ Grandparent	☐ Aunt/Uncle/Cousin	☐ Childcare facility outside home	☐ Childminder/ day care at home

**How many days a week is the child in day care?**	☐ N/A	☐ 1-2	☐ 3-4	☐ 5-6	☐ >6	
**How many hours per day is the child in day care?**	☐ N/A	☐ 1-4h	☐ 5-8h	☐ 9-12h	☐ >12h	
**How many children are looked after at this day care?**	☐ <3	☐ 4-6	☐ 7-10	☐ >10	☐Unknown	☐ N/A
**How many of these are under 2y?**	☐ <3	☐ 4-6	☐ 7-10	☐ >10	☐Unknown	☐ N/A
**How many adults look after these children?**	☐ 1	☐2-4	☐5-10	☐ >10	☐ N/A	
**Do you feel the day care is good?**	☐ Y	☐ N	☐ N/A			

**Who provides food for the child at day care?** *Select one*				
☐ Caregiver provides food for the child	☐ Day care provides food for the child	☐ Someone else provides food for the child	☐ Don't know	☐ N/A

**Is feeding supervised / assisted at day care?**	☐ Y	☐ N	☐ Unknown	☐ N/A

**Household Food Security** (if child in care home include **children** in the care home only)			
**During the past 7 DAYS** has ANY member of the household missed a meal due to food shortage?	☐ Y	☐ N	☐ Unknown
**During the past 4 WEEKS**			
Did you worry that your household would not have enough food?	☐ Y	☐ N	☐ Unknown
Were any of your household unable to eat the kinds of food preferred because of a lack of resources?	☐ Y	☐ N	☐ Unknown
Have any of your household had to eat a limited variety of food due to lack of resources?	☐ Y	☐ N	☐ Unknown
Have any of your household eaten some foods that you really didn't want to eat because of lack of resources?	☐ Y	☐ N	☐ Unknown
Have any of your household eaten fewer meals in a day because there was not enough food?	☐ Y	☐ N	☐ Unknown
Did household members go to sleep at night hungry because there was not enough food?	☐ Y	☐ N	☐ Unknown
Did you or your household members go a whole day and night without eating anything because there was not enough food?	☐ Y	☐ N	☐ Unknown

For peer review only - http://bmjopen.bmj.com/site/about/guidelines.xhtml

CHAIN Enrolment CRF v1.63

CHAIN Number [1][0] [0][0][3] [ ][ ][ ]

Child Dietary Diversity
What does the child eat on a typical day? • *Ask this as an open question and select all that the caregiver mentions.* • *Do not present the caregiver with this list.* • *You may prompt the caregiver with open questions, e.g. What does your child usually eat for breakfast*
☐ **Milk and Milk Products**: Fresh/fermented milk, cheese, yogurt, or other milk products
☐ **Breast milk**
☐ **Cereals and Cereal Products**: Maize, rice, pasta, porridge, bread, biscuits, millet, sorghum, wheat, locally available grains
☐ **Fish and Sea Foods**: fresh or dried fish or shellfish
☐ **Roots and Tubers**: potatoes, sweet potatoes, yams, cassava, or foods made from roots or wild roots and tubers
☐ **Vegetables**: Cabbages, carrots, spinach, and any other locally available vegetables including wild vegetables
☐ **Fruits**: Oranges, bananas, mangoes, avocados, apples, grapes etc
☐ **Meats and Poultry**: Camel, beef, lamb, goat, rabbit, wild game, chicken or other birds, liver, kidney, heart or other organ meats or blood-based foods
☐ **Eggs**: Hen or other bird eggs
☐ **Pulses / Legumes / Nuts and Seeds**: Beans, peas, lentils, nuts, seeds or foods made from these
☐ **Fats and Oils**: Oil, fats, ghee, margarine or butter added to food or used for cooking
☐ **Sugars / Honey and Commercial Juices**: Sugar in tea, honey, sweetened soda, juices, chocolates, sweets or candies
☐ **Miscellaneous**: Spices, unsweetened beverages
☐ **UNKNOWN**

Feeding practices	
**How is food USUALLY given to the child? *Select one***	
☐ Fed by adult	☐ Child feeds self, unsupervised
☐ Child feeds self, supervised by adult	☐ Fed from common plate or bowl
☐ Child feeds self, supervised by older children	☐ Child exclusively breastfed
☐ Unknown	☐ Other

For peer review only - http://bmjopen.bmj.com/site/about/guidelines.xhtml

CHAIN Enrolment CRF v1.63

CHAIN Number [1][0] [0][0][3] [ ][ ][ ]

Assessment of household wealth<br>(DHS 7 questionnaire. Please answer all questions, for all participants, including children in care homes)		
**What is the main source of drinking water for members of your household?** *Choose one*		
☐ Piped water to dwelling	☐ Cart with small tank	☐ from vendor
☐ Piped water to yard / plot	☐ Tanker truck	☐ Rainwater
☐ Piped to neighbour	☐ Bottled water	☐ Stream/river/lake/pond/dam
☐ Public tap/ Standpipe	☐ Protected spring	☐ Unknown
☐ Protected well / borehole	☐ Unprotected spring	
☐ Unprotected well	☐ Other	
**What is the MAIN source of water used by your household for other purposes such as cooking and handwashing? SELECT ONE ONLY**		
☐ Piped water to dwelling	☐ Cart with small tank	☐ Bought from vendor
☐ Piped water to yard / plot	☐ Tanker truck	☐ Rainwater
☐ Piped to neighbour	☐ Bottled water	☐ Stream/river/lake/pond/dam
☐ Public tap/ Standpipe	☐ Protected spring	☐ Unknown
☐ Protected well / borehole	☐ Unprotected spring	
☐ Unprotected well	☐ Other	

**How long does it take to get DRINKING water and come back? (State 0 if water supplied within home or compound)**	__ __ __minutes	☐ Don't know
**In the past 2 weeks was the water from this source not available for at least one full day?**	☐ Y ☐ N	☐ Unknown

**Do you usually do anything to the water to make it safer to drink?** *Select all that apply*			
☐ None	☐ Bleach/ chlorine	☐ Strain through a cloth	☐ Let it stand and settle
☐ Use water filter (ceramic/sand/composite etc)	☐ Solar disinfection	☐ Boil	☐ Other

For peer review only

For peer review only - http://bmjopen.bmj.com/site/about/guidelines.xhtml

CHAIN Enrolment CRF v1.63

CHAIN Number [1][0] [0][0][3] [ ][ ][ ]

**What kind of toilet facility do members of your household usually use?** *Select one*		
☐ Flush or pour flush toilet to piped sewer	☐ Flush to septic tank	☐ Ventilated improved pit latrine
☐ Flush to pit latrine	☐ Flush to somewhere else	☐ Open pit / Pit latrine without slab
☐ Flush don't know where	☐ Composting toilet	☐ Bucket toilet
☐ Pit latrine with slab	☐ Hanging toilet / hanging latrine	☐ No facility / bush/ field
☐ Unknown		

**Do you share this toilet facility with other households?**	☐ Y	☐ N	☐ Unknown	
**If Yes, including your own household, how many households use this toilet facility?**	Number if <10__	☐ >10 households	☐ Unknown	☐ N/A
**Where is this toilet facility located?**	☐ In own dwelling	☐ In own yard / plot	☐ Elsewhere	
**How many rooms are there in the household for SLEEPING?**	☐ **1**	☐ **2**	☐ **>2**	

**What is the MAIN FLOOR material of the rooms in this household?** *Select one only*		
☐ Cement	☐ Earth/sand	☐ Wood
☐ Dung	☐ Lives on boat	☐ Tiles
☐ Carpet	☐ Other (specify)____________________	☐ Unknown

**What is the MAIN WALL material of the rooms in this household?** *Select one only*			
☐ Grass/straw/makuti	☐ Stone	☐ Wood	☐ Unknown
☐ Corrugated iron sheet/ Tin	☐ Mud/wood	☐ Brick/block	
☐ Planks/shingles	☐ No wall	☐ Other (specify) __________________	

**What is the MAIN ROOF material of the house in this household?** *Select one only*		
☐ Grass/Thatch	☐ Tiles/Asbestos sheets	☐ Corrugated iron/ Tins
☐ Mud	☐ Nylon papers/clothes	☐ Concrete
☐ Other (specify) _________________________		☐ Unknown

**What is the MAIN cooking fuel used in this household?** *Select one only*		
☐ Electricity	☐ LPG /Natural gas/Biogas	☐ Paraffin
☐ Coal / Lignite	☐ Charcoal	☐ Firewood
☐ Straw/shrubs/grass	☐ Agricultural crop	☐ Animal Dung
☐ No food cooked in household	☐ Other (specify)_____________________	☐ Unknown

**Do you have a separate room which is used as a kitchen?**	☐ Y	☐ N	☐ Unknown

**Where is this household's cooking area located?**				
☐ In the house	☐ Outdoors	☐ In a separate building	☐ Other ______________	☐ Unknown

For peer review only

For peer review only - http://bmjopen.bmj.com/site/about/guidelines.xhtml

CHAIN Enrolment CRF v1.63
CHAIN Number [1][0] [0][0][3] [ ][ ][ ]

**Does this household own any livestock, herds, other farm animals or poultry**	☐ Y	☐ N	☐ Unknown
**If yes, how many of the following animals does this household own?**			
Cows/bulls__ __ Sheep__ __<br>Horses/Donkeys/Mules__ __ Goats__ __<br>Chickens or Ducks__ __ Other ______________ number ___ ___			☐ N/A
**Does any member of this this household own land?**	☐ Y	☐ N	☐ Unknown
**If "Yes" How many acres of land does this household own?**	_____Acres	☐ Unknown	☐ N/A
**Does this household have a bank account?**	☐ Y	☐ N	☐ Unknown
**Does this household have electricity**	☐ Y	☐ N	☐ Unknown
**Does this household own a radio?**	☐ Y	☐ N	☐ Unknown
**Does this household own a television?**	☐ Y	☐ N	☐ Unknown
**Does this household own a computer?**	☐ Y	☐ N	☐ Unknown
**Does this household own a refrigerator?**	☐ Y	☐ N	☐ Unknown
**Does any member of this household own:**			
**A watch**	☐ Y	☐ N	☐ Unknown
**A mobile phone?**	☐ Y Standard phone / ☐ Y smartphone	☐ N	☐ Unknown
**An animal-drawn cart?**	☐ Y	☐ N	☐ Unknown
**A bicycle?**	☐ Y	☐ N	☐ Unknown
**A motorcycle / scooter?**	☐ Y	☐ N	☐ Unknown
**A car or truck?**	☐ Y	☐ N	☐ Unknown
**A boat with a motor?**	☐ Y	☐ N	☐ Unknown

**CRF Completed by (Initials) – to be signed when complete.**<br>*Do not sign if any fields are empty*	__ __ __	Date<br>__ __/__ __/__ __ __ __<br>*D D / M M / Y Y Y Y*	Time<br>__ __:__ __

END

For peer review only - http://bmjopen.bmj.com/site/about/guidelines.xhtml

STROBE (Strengthening The Reporting of OBservational Studies in Epidemiology) Checklist

A checklist of items that should be included in reports of observational studies. You must report the page number in your manuscript where you consider each of the items listed in this checklist. If you have not included this information, either revise your manuscript accordingly before submitting or note N/A.

Note: An Explanation and Elaboration article discusses each checklist item and gives methodological background and published examples of transparent reporting. The STROBE checklist is best used in conjunction with this article (freely available on the Web sites of PLoS Medicine at http://www.plosmedicine.org/, Annals of Internal Medicine at http://www.annals.org/, and Epidemiology at http://www.epidem.com/). Information on the STROBE Initiative is available at www.strobe-statement.org.

Section and Item	Item No.	Recommendation	Reported on Page No.
**Title and Abstract**	1	(*a*) Indicate the study's design with a commonly used term in the title or the abstract	1
		(*b*) Provide in the abstract an informative and balanced summary of what was done and what was found	1
**Introduction**			
Background/Rationale	2	Explain the scientific background and rationale for the investigation being reported	
Objectives	3	State specific objectives, including any prespecified hypotheses	
**Methods**			
Study Design	4	Present key elements of study design early in the paper	4
Setting	5	Describe the setting, locations, and relevant dates, including periods of recruitment, exposure, follow-up, and data collection	5
Participants	6	(*a*) *Cohort study*—Give the eligibility criteria, and the sources and methods of selection of participants. Describe methods of follow-up	4-6
		Case-control study—Give the eligibility criteria, and the sources and methods of case ascertainment and control selection. Give the rationale for the choice of cases and controls	NA
		Cross-sectional study—Give the eligibility criteria, and the sources and methods of selection of participants	NA
		(*b*) *Cohort study*—For matched studies, give matching criteria and number of exposed and unexposed	NA
		Case-control study—For matched studies, give matching criteria and the number of controls per case	NA
Variables	7	Clearly define all outcomes, exposures, predictors, potential confounders, and effect modifiers. Give diagnostic criteria, if applicable	6-8

For peer review only

Section and Item	Item No.	Recommendation	Reported on Page No.
Data Sources/ Measurement	8*	For each variable of interest, give sources of data and details of methods of assessment (measurement). Describe comparability of assessment methods if there is more than one group	6-9
Bias	9	Describe any efforts to address potential sources of bias	NA
Study Size	10	Explain how the study size was arrived at	11
Quantitative Variables	11	Explain how quantitative variables were handled in the analyses. If applicable, describe which groupings were chosen and why	10-11
Statistical Methods	12	(*a*) Describe all statistical methods, including those used to control for confounding	10-11
		(*b*) Describe any methods used to examine subgroups and interactions	10-11
		(*c*) Explain how missing data were addressed	NA
		(*d*) *Cohort study*—If applicable, explain how loss to follow-up was addressed	10-11
		Case-control study—If applicable, explain how matching of cases and controls was addressed	NA
		Cross-sectional study—If applicable, describe analytical methods taking account of sampling strategy	NA
		(*e*) Describe any sensitivity analyses	NA
**Results**			
Participants	13*	(a) Report numbers of individuals at each stage of study—eg numbers potentially eligible, examined for eligibility, confirmed eligible, included in the study, completing follow-up, and analysed	NA
		(b) Give reasons for non-participation at each stage	NA
		(c) Consider use of a flow diagram	NA
Descriptive Data	14*	(a) Give characteristics of study participants (eg demographic, clinical, social) and information on exposures and potential confounders	NA
		(b) Indicate number of participants with missing data for each variable of interest	NA
		(c) *Cohort study*—Summarise follow-up time (eg, average and total amount)	NA
Outcome Data	15*	*Cohort study*—Report numbers of outcome events or summary measures over time	NA
		Case-control study—Report numbers in each exposure category, or summary measures of exposure	NA
		Cross-sectional study—Report numbers of outcome events or summary measures	NA

For peer review only

Section and Item	Item No.	Recommendation	Reported on Page No.
Main Results	16	(*a*) Give unadjusted estimates and, if applicable, confounder-adjusted estimates and their precision (eg, 95% confidence interval). Make clear which confounders were adjusted for and why they were included	NA
		(*b*) Report category boundaries when continuous variables were categorized	NA
		(*c*) If relevant, consider translating estimates of relative risk into absolute risk for a meaningful time period	NA
Other Analyses	17	Report other analyses done—eg analyses of subgroups and interactions, and sensitivity analyses	NA
**Discussion**			
Key Results	18	Summarise key results with reference to study objectives	NA
Limitations	19	Discuss limitations of the study, taking into account sources of potential bias or imprecision. Discuss both direction and magnitude of any potential bias	NA
Interpretation	20	Give a cautious overall interpretation of results considering objectives, limitations, multiplicity of analyses, results from similar studies, and other relevant evidence	NA
Generalisability	21	Discuss the generalisability (external validity) of the study results	NA
**Other Information**			
Funding	22	Give the source of funding and the role of the funders for the present study and, if applicable, for the original study on which the present article is based	13

*Give information separately for cases and controls in case-control studies and, if applicable, for exposed and unexposed groups in cohort and cross-sectional studies.

Once you have completed this checklist, please save a copy and upload it as part of your submission. DO NOT include this checklist as part of the main manuscript document. It must be uploaded as a separate file.

For peer review only
